# Supplementary figures and images for: Novel autophagy inducers by accelerating lysosomal clustering against Parkinson’s disease
Source: eLife. 2024 Jul 3;13:e98649. doi: 10.7554/eLife.98649 (PMC11221835; doi:10.7554/eLife.98649)

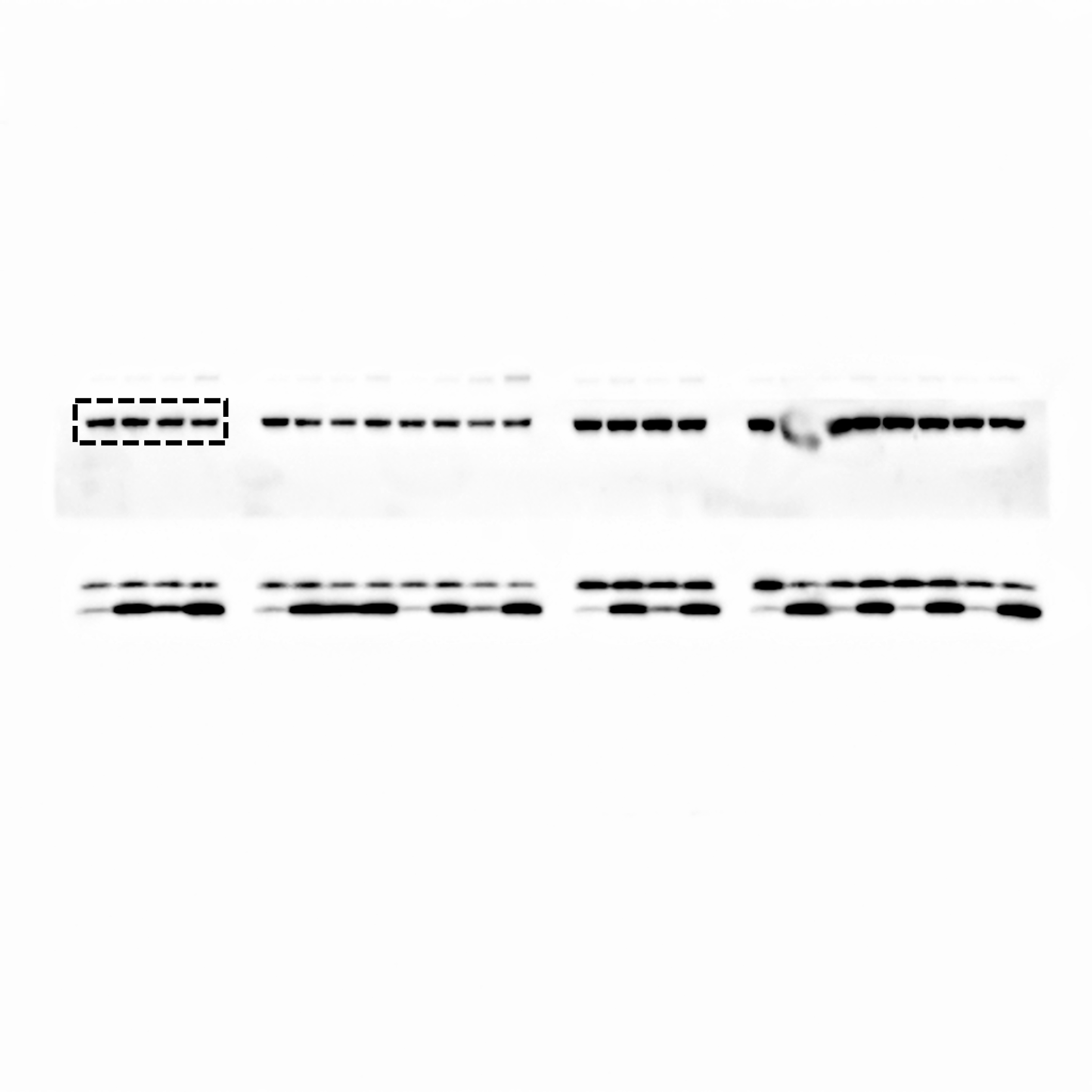

Supplement: Figure 2—source data 1. [file elife-98649-fig2-data1.zip › Figure 2-source data1/Figure 2C_actin_Albendazole_annotated.tif]

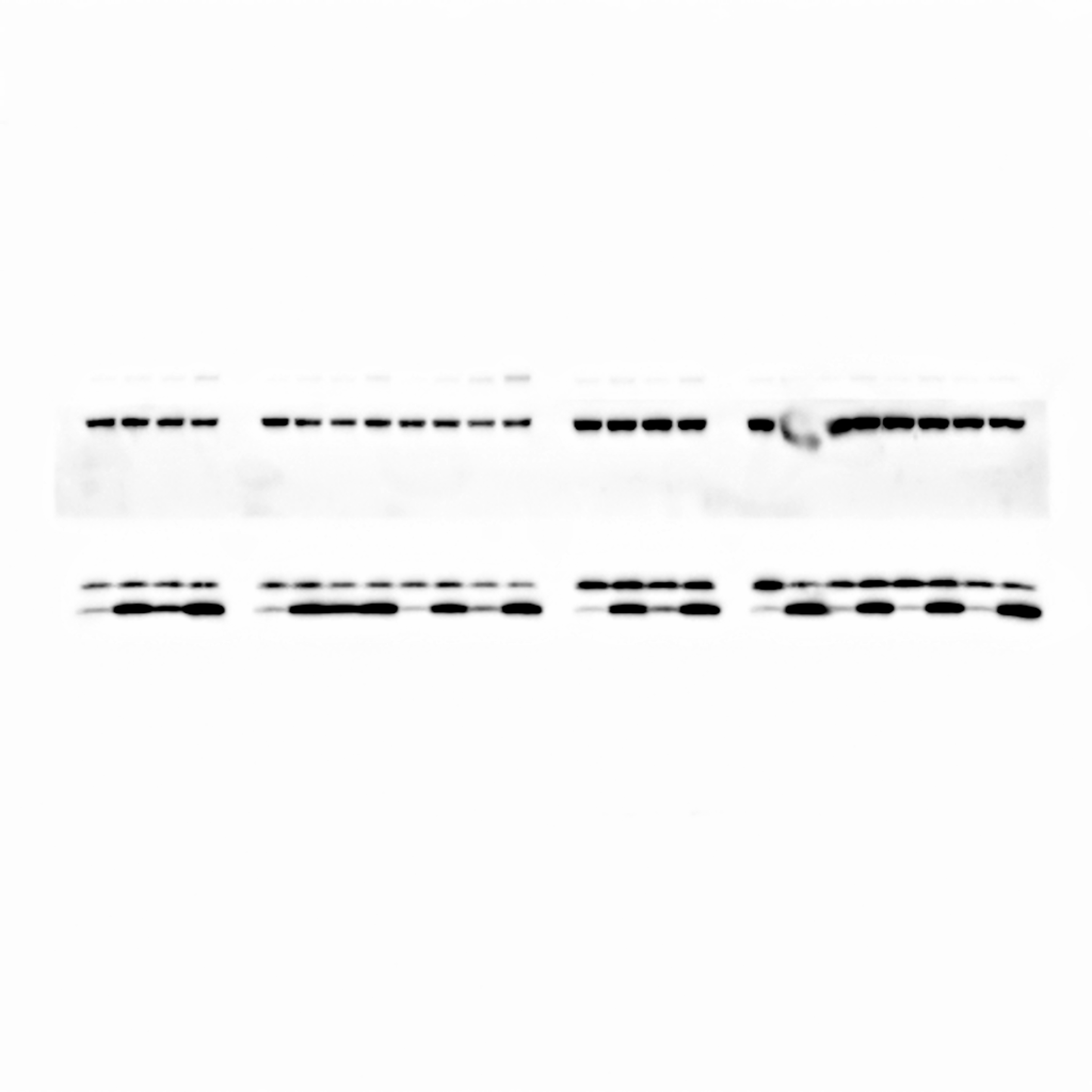

Supplement: Figure 2—source data 1. [file elife-98649-fig2-data1.zip › Figure 2-source data1/Figure 2C_actin_Albendazole_raw.tif]

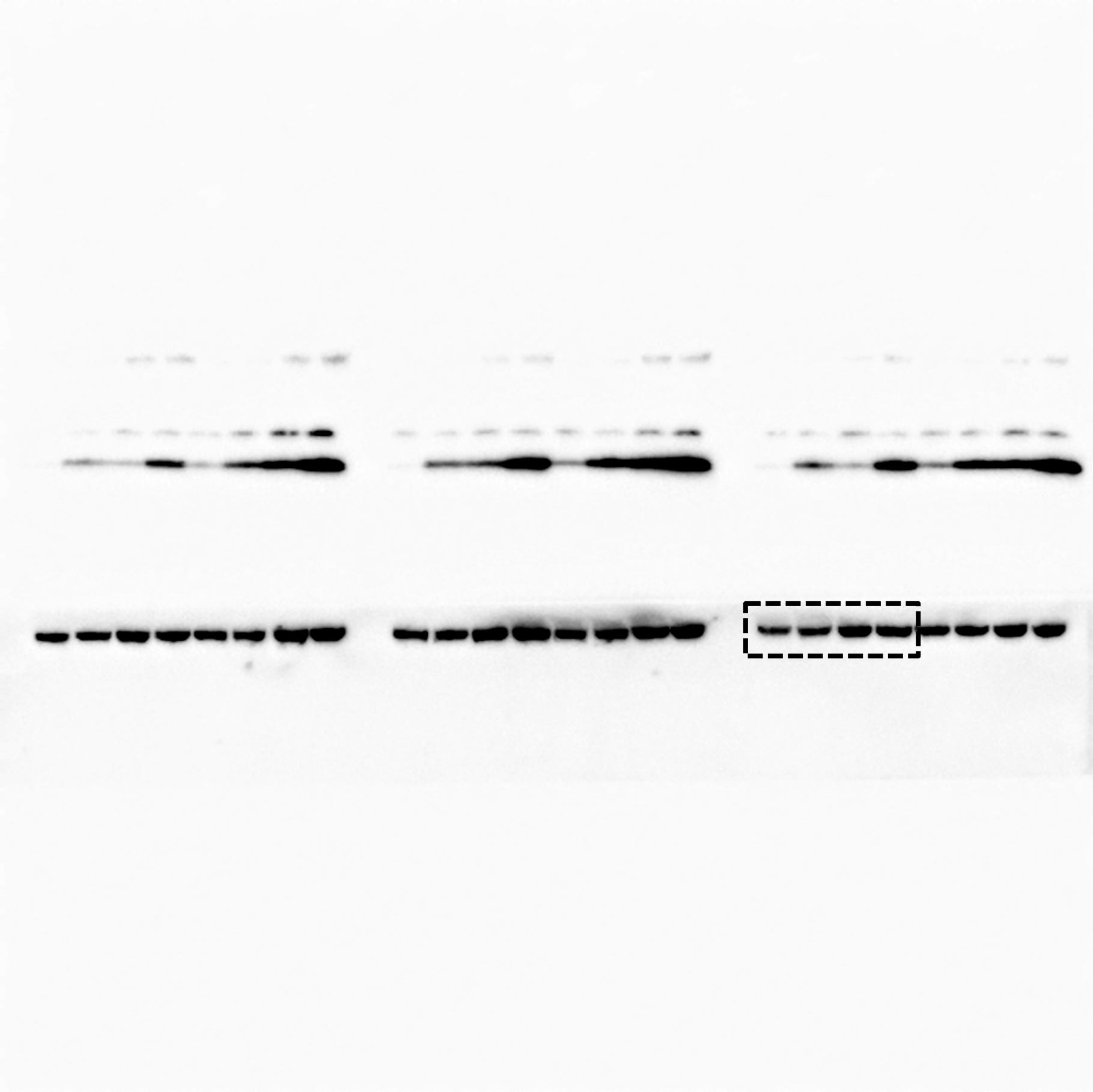

Supplement: Figure 2—source data 1. [file elife-98649-fig2-data1.zip › Figure 2-source data1/Figure 2C_actin_Etoposide_anotated.tif]

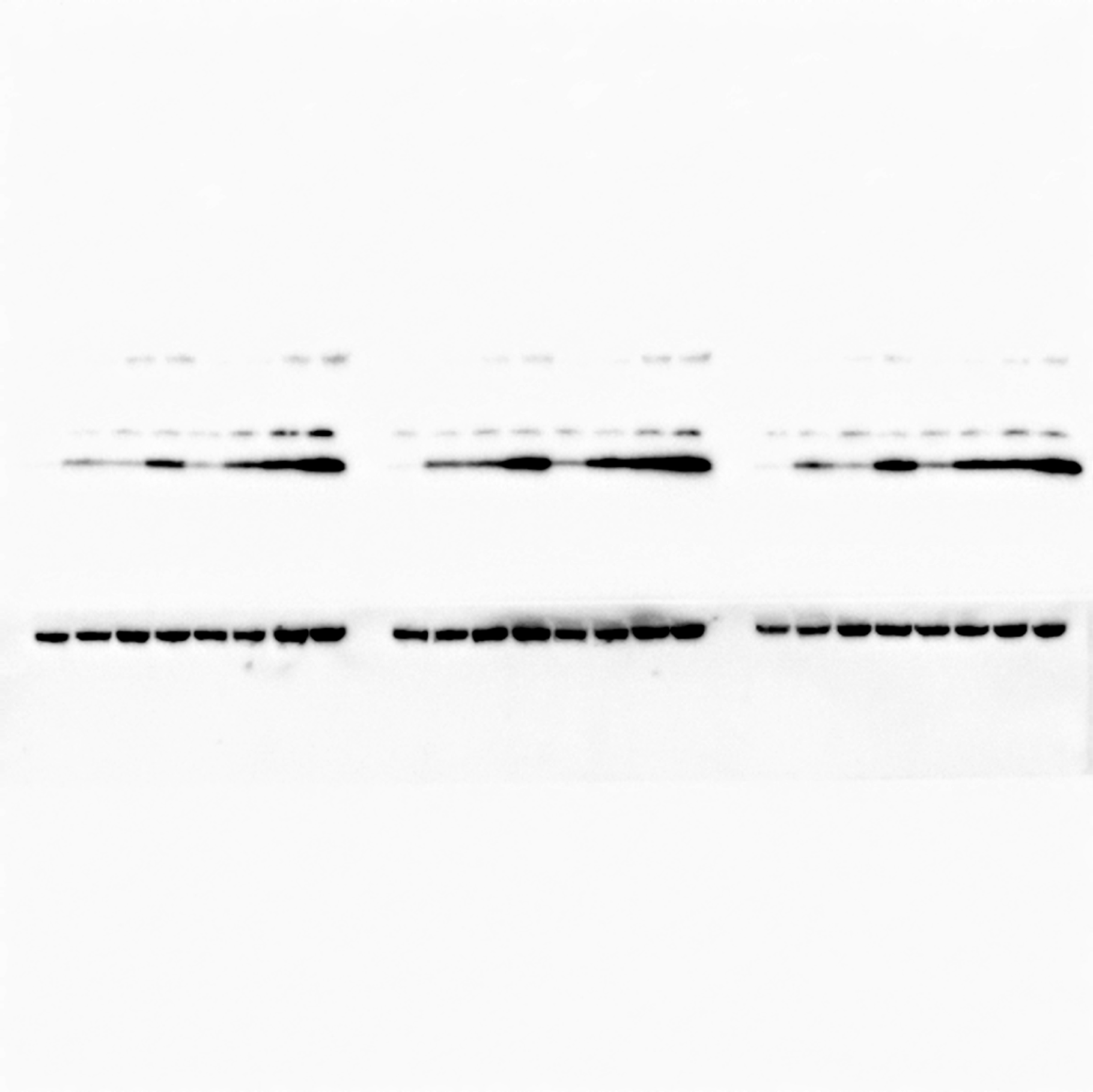

Supplement: Figure 2—source data 1. [file elife-98649-fig2-data1.zip › Figure 2-source data1/Figure 2C_actin_Etoposide_raw.tif]

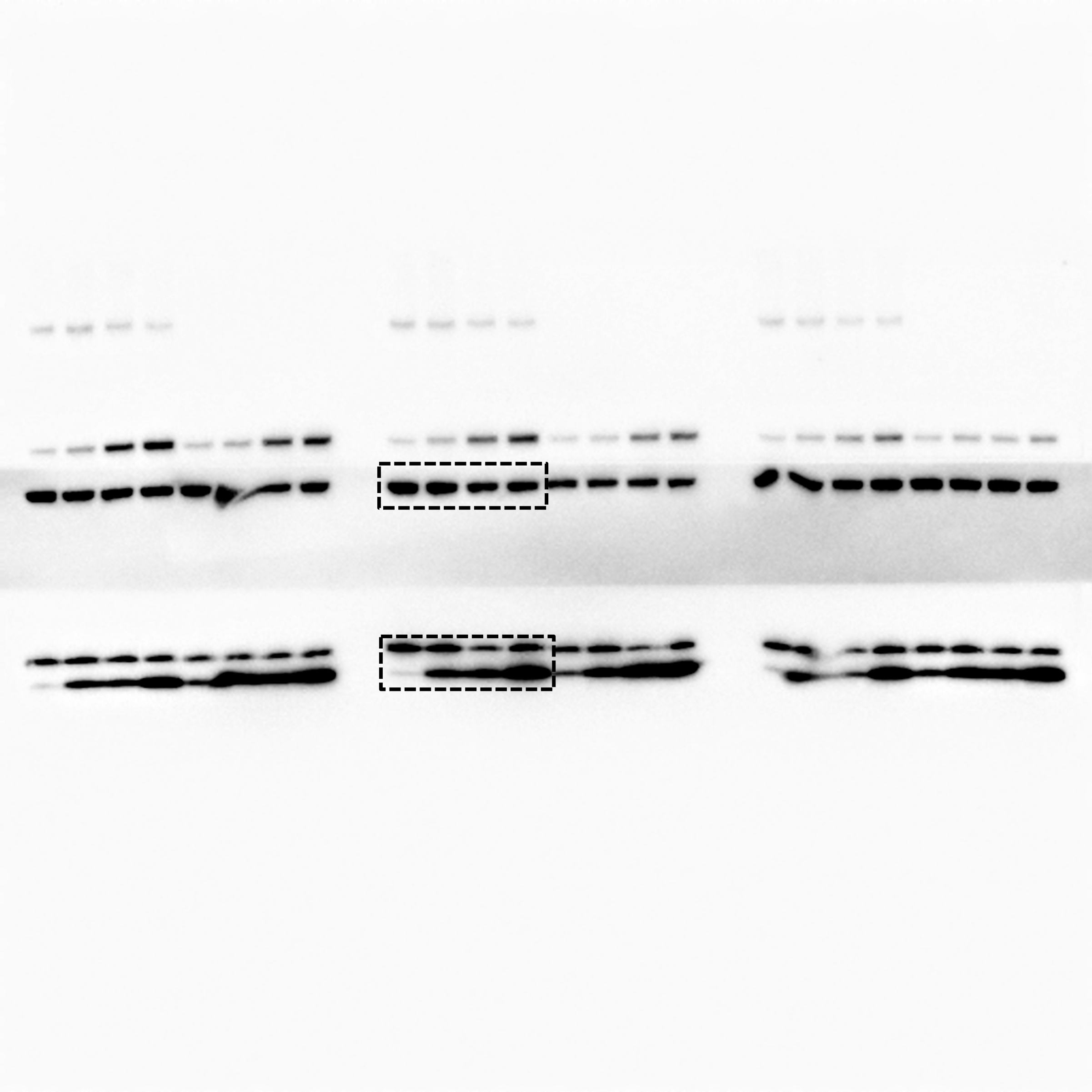

Supplement: Figure 2—source data 1. [file elife-98649-fig2-data1.zip › Figure 2-source data1/Figure 2C_LC3B_actin_Amsacrine_annotated.tif]

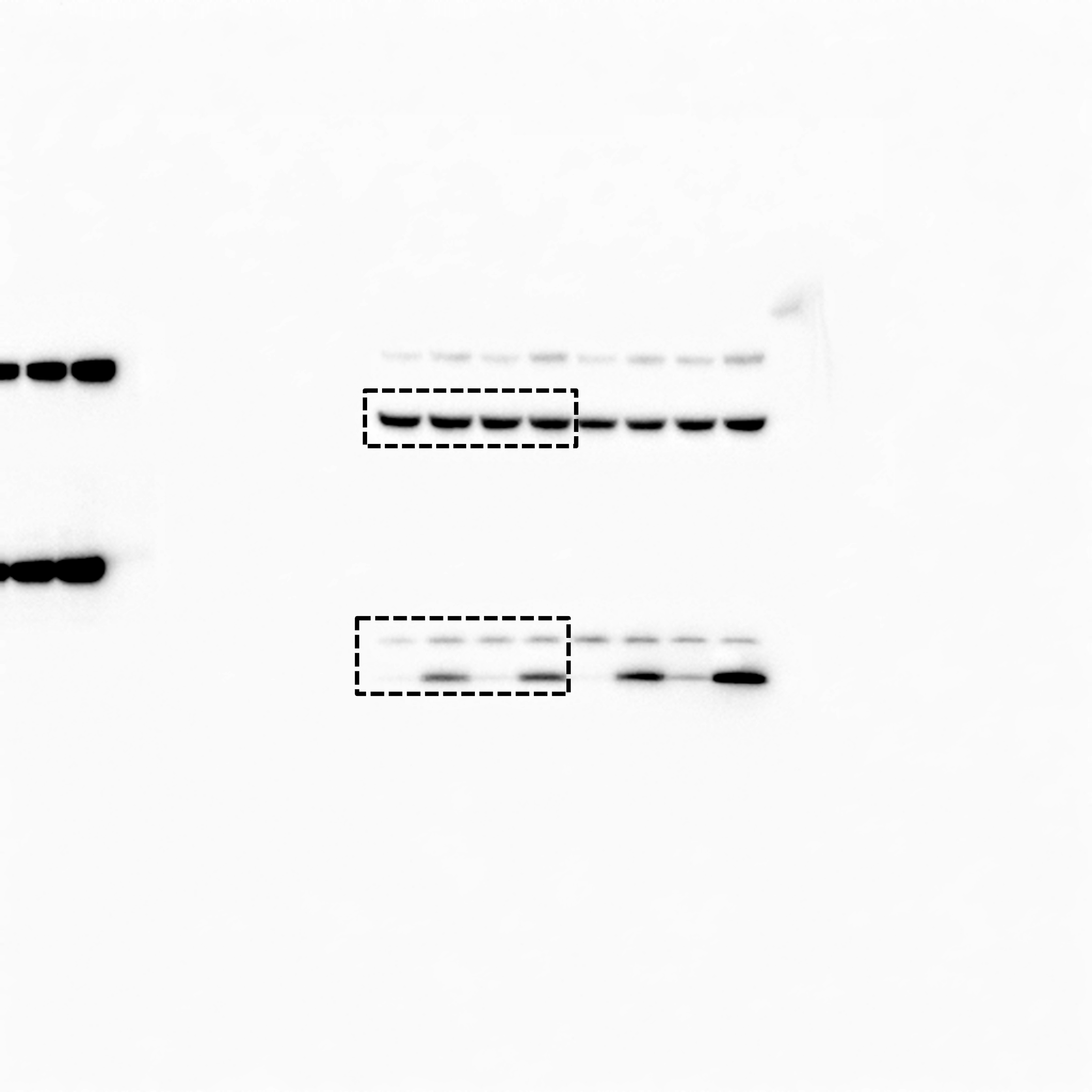

Supplement: Figure 2—source data 1. [file elife-98649-fig2-data1.zip › Figure 2-source data1/Figure 2C_LC3B_actin_Mebendazole_annotated.tif]

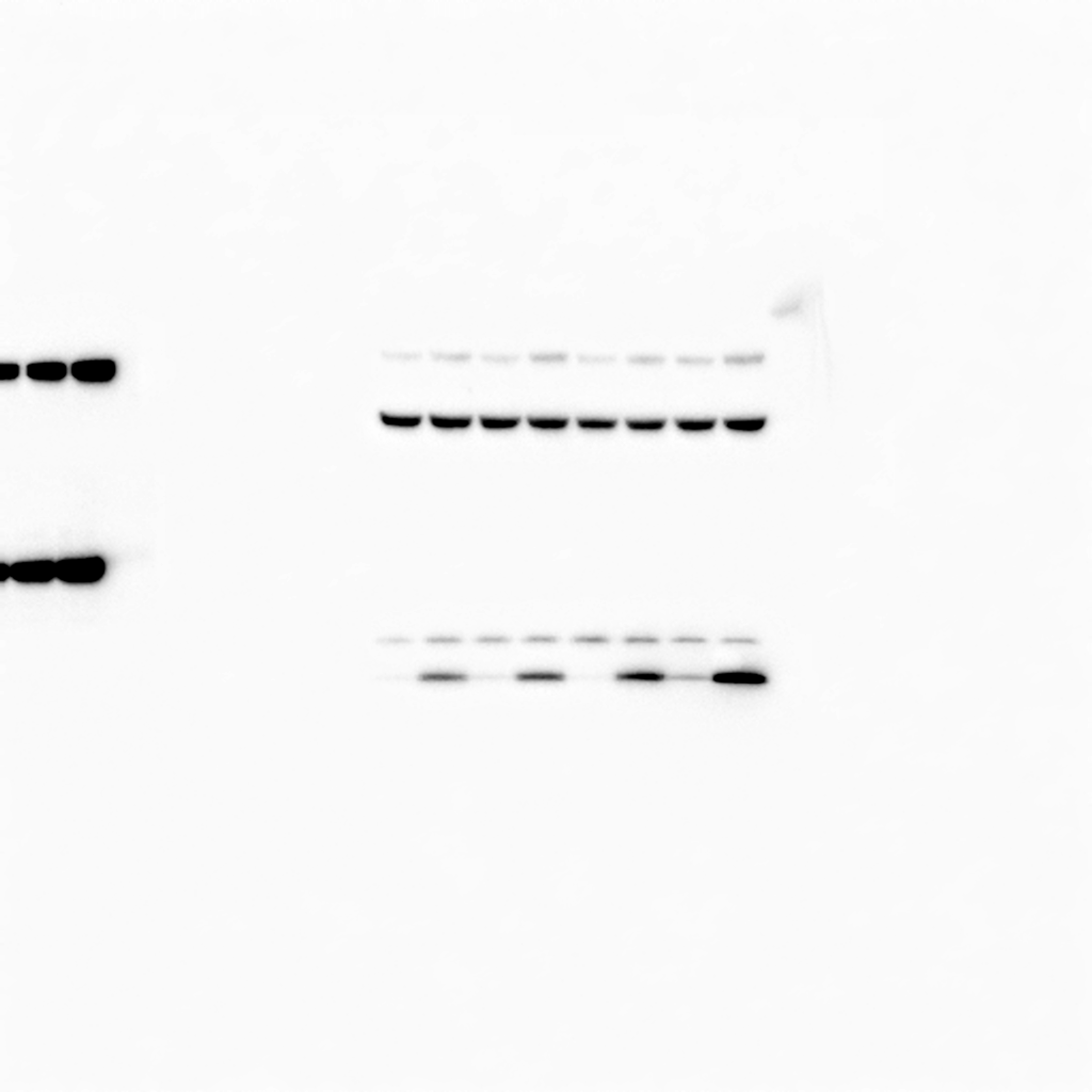

Supplement: Figure 2—source data 1. [file elife-98649-fig2-data1.zip › Figure 2-source data1/Figure 2C_LC3B_actin_Mebendazole_raw.tif]

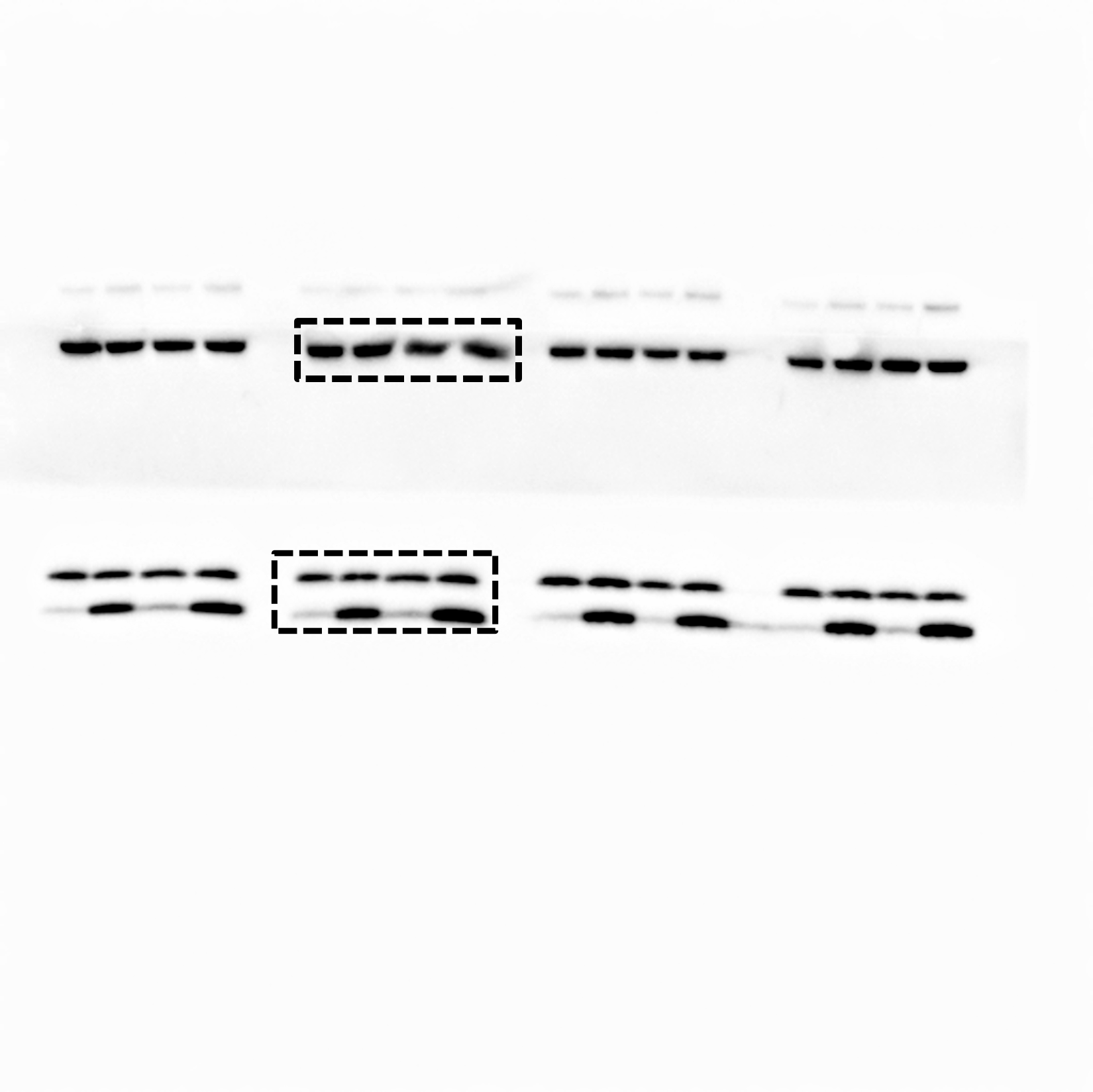

Supplement: Figure 2—source data 1. [file elife-98649-fig2-data1.zip › Figure 2-source data1/Figure 2C_LC3B_actin_Oxibendazole_annotated.tif]

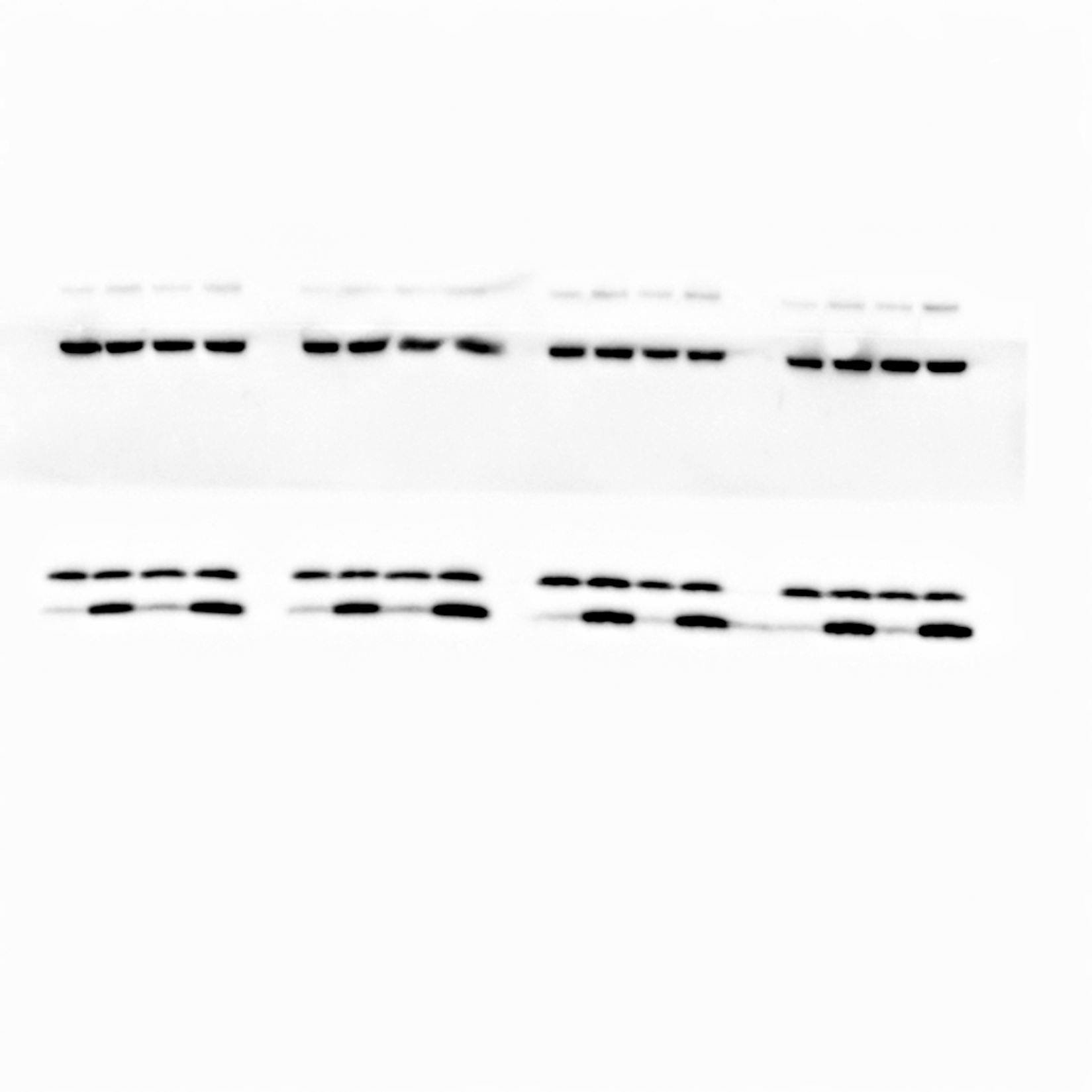

Supplement: Figure 2—source data 1. [file elife-98649-fig2-data1.zip › Figure 2-source data1/Figure 2C_LC3B_actin_Oxibendazole_raw.tif]

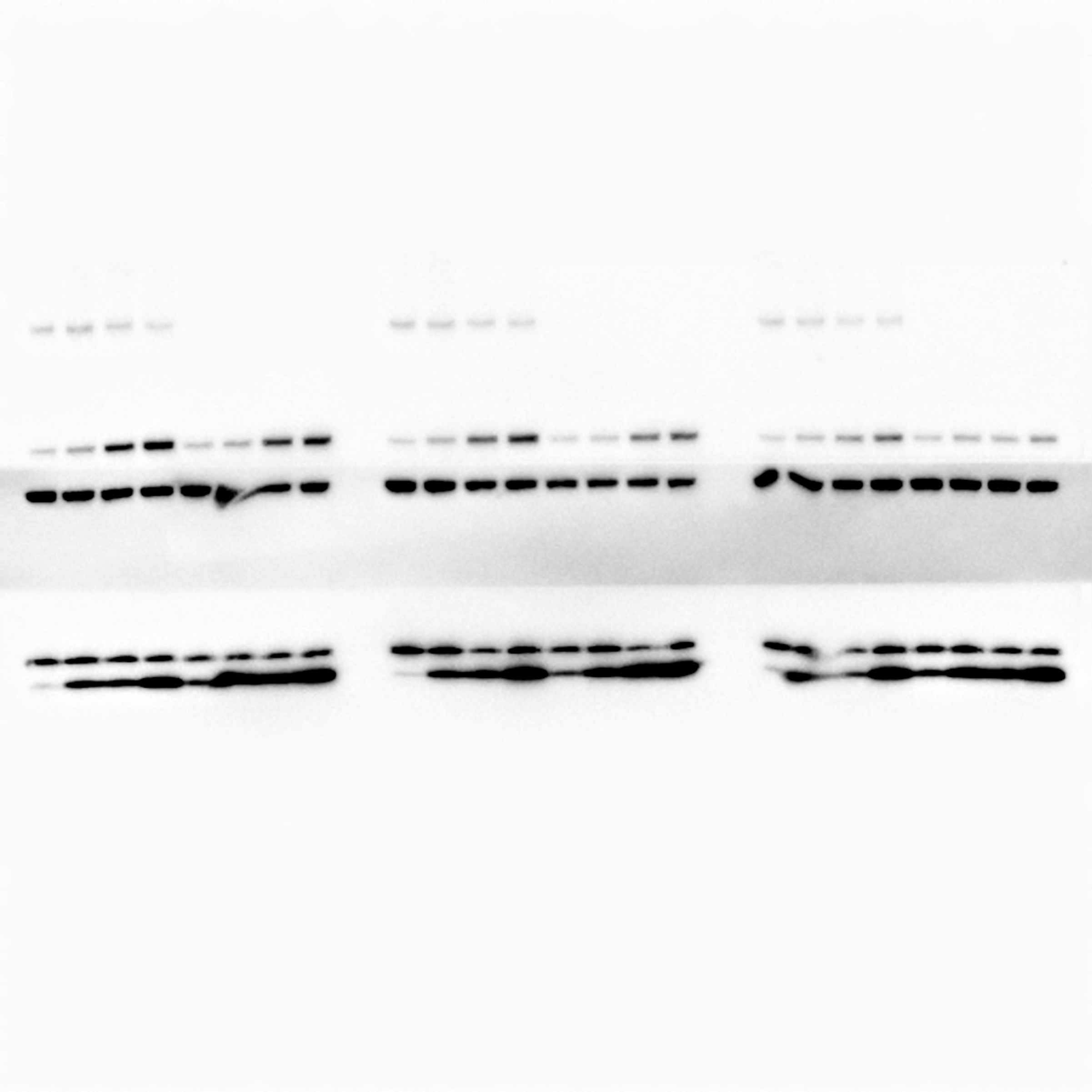

Supplement: Figure 2—source data 1. [file elife-98649-fig2-data1.zip › Figure 2-source data1/Figure 2C_LC3B_actin_Teniposide_Amsacrine_raw.tif]

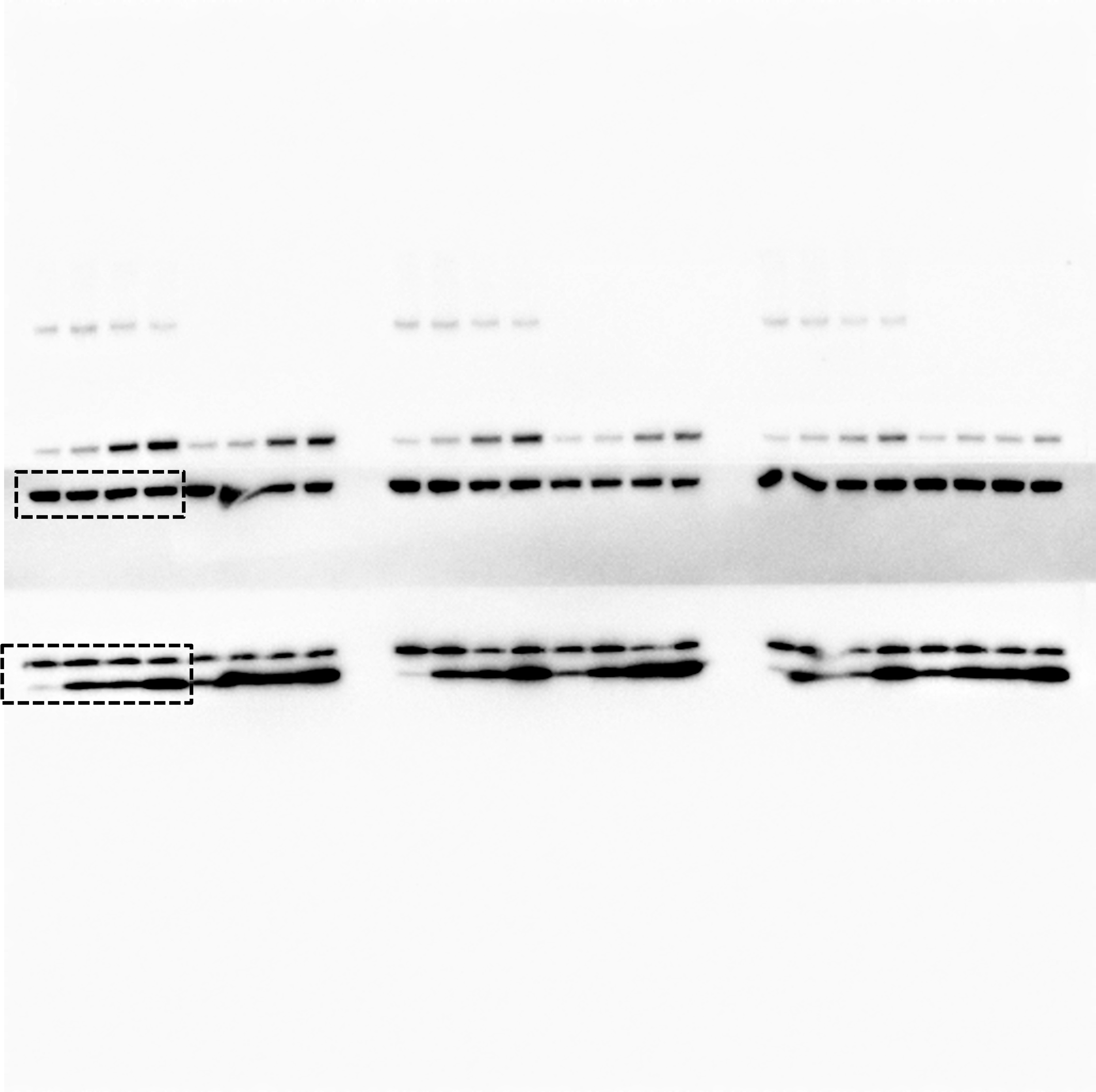

Supplement: Figure 2—source data 1. [file elife-98649-fig2-data1.zip › Figure 2-source data1/Figure 2C_LC3B_actin_Teniposide_annotated.tif]

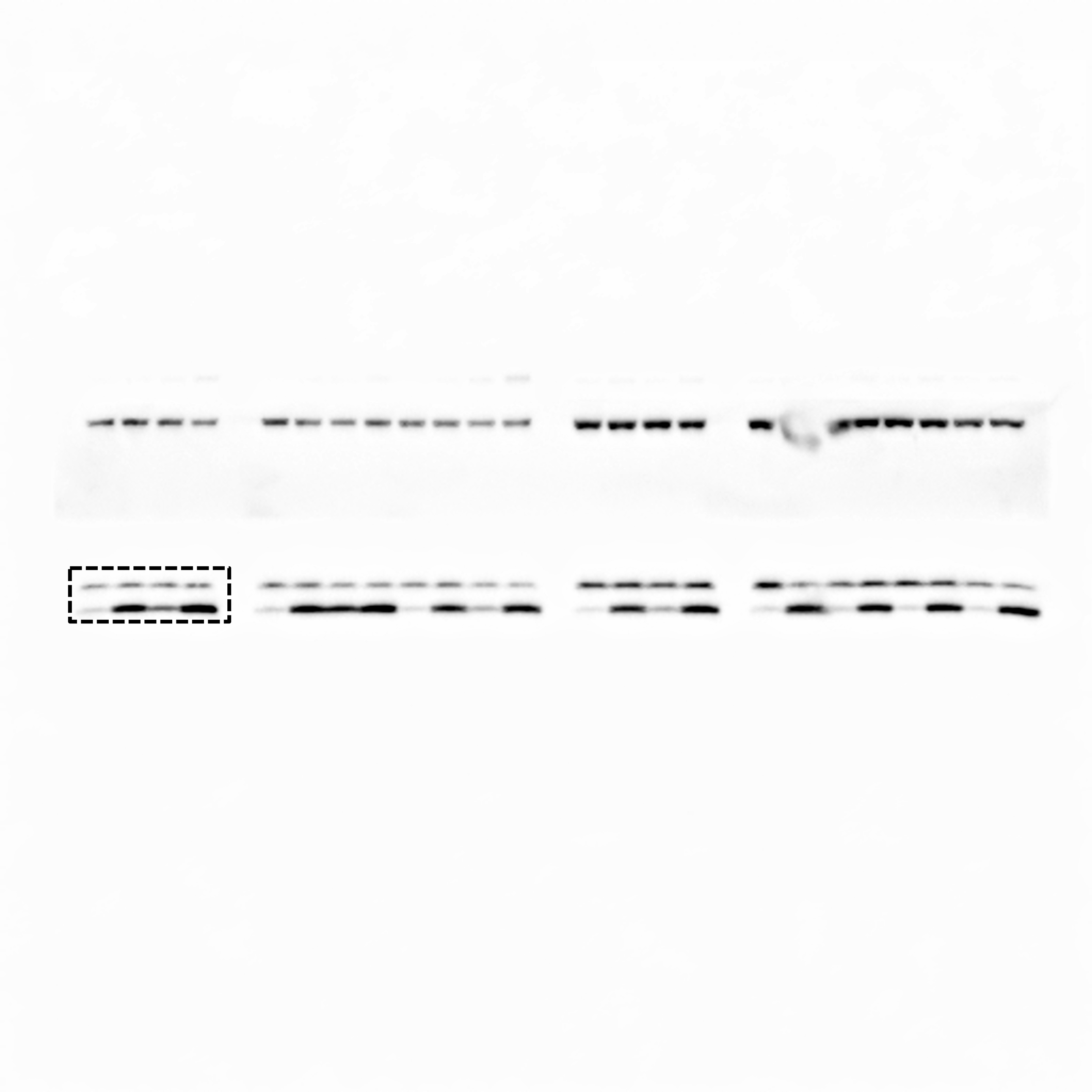

Supplement: Figure 2—source data 1. [file elife-98649-fig2-data1.zip › Figure 2-source data1/Figure 2C_LC3B_Albendazole_annotated.tif]

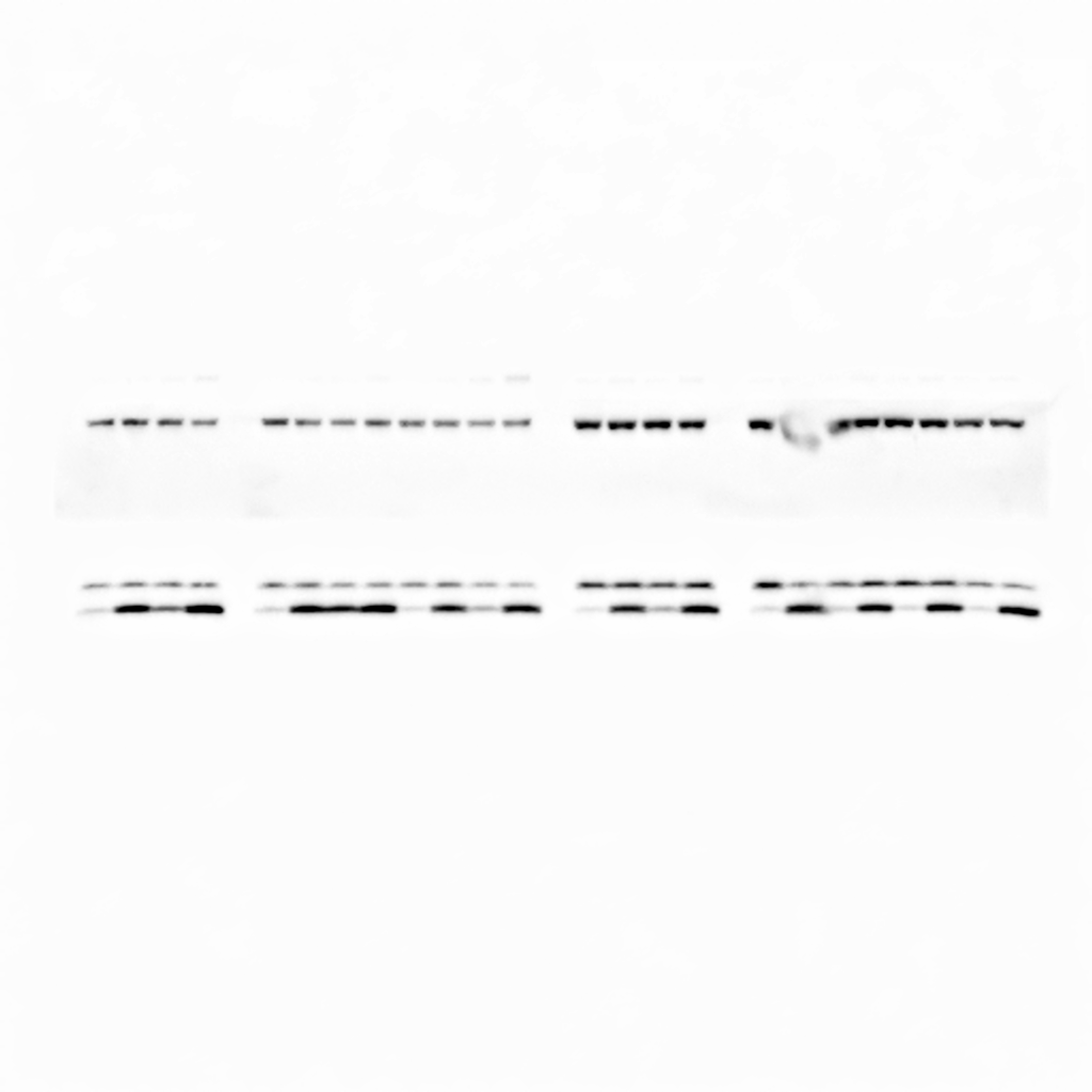

Supplement: Figure 2—source data 1. [file elife-98649-fig2-data1.zip › Figure 2-source data1/Figure 2C_LC3B_Albendazole_raw.tif]

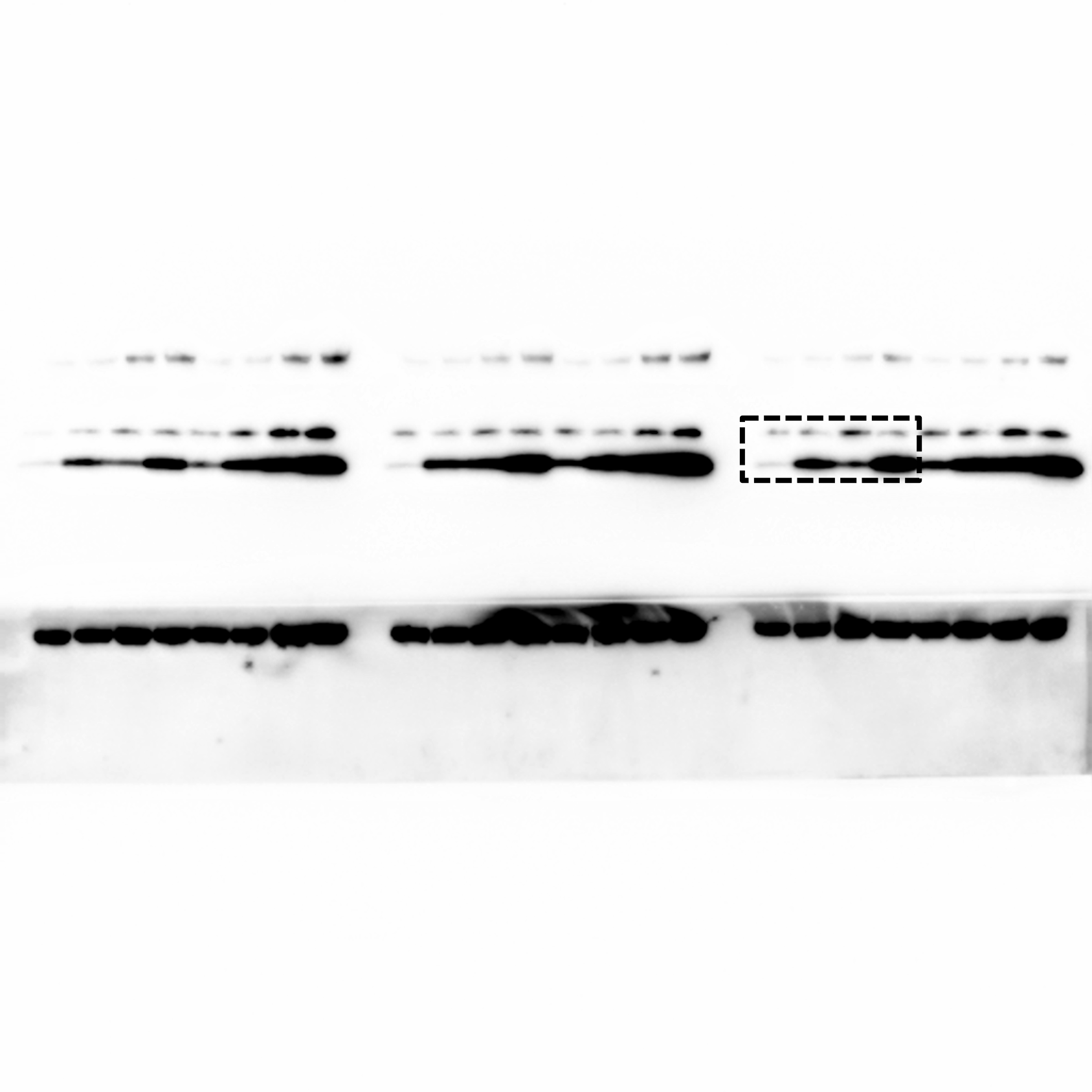

Supplement: Figure 2—source data 1. [file elife-98649-fig2-data1.zip › Figure 2-source data1/Figure 2C_LC3B_Etoposide_annotated.tif]

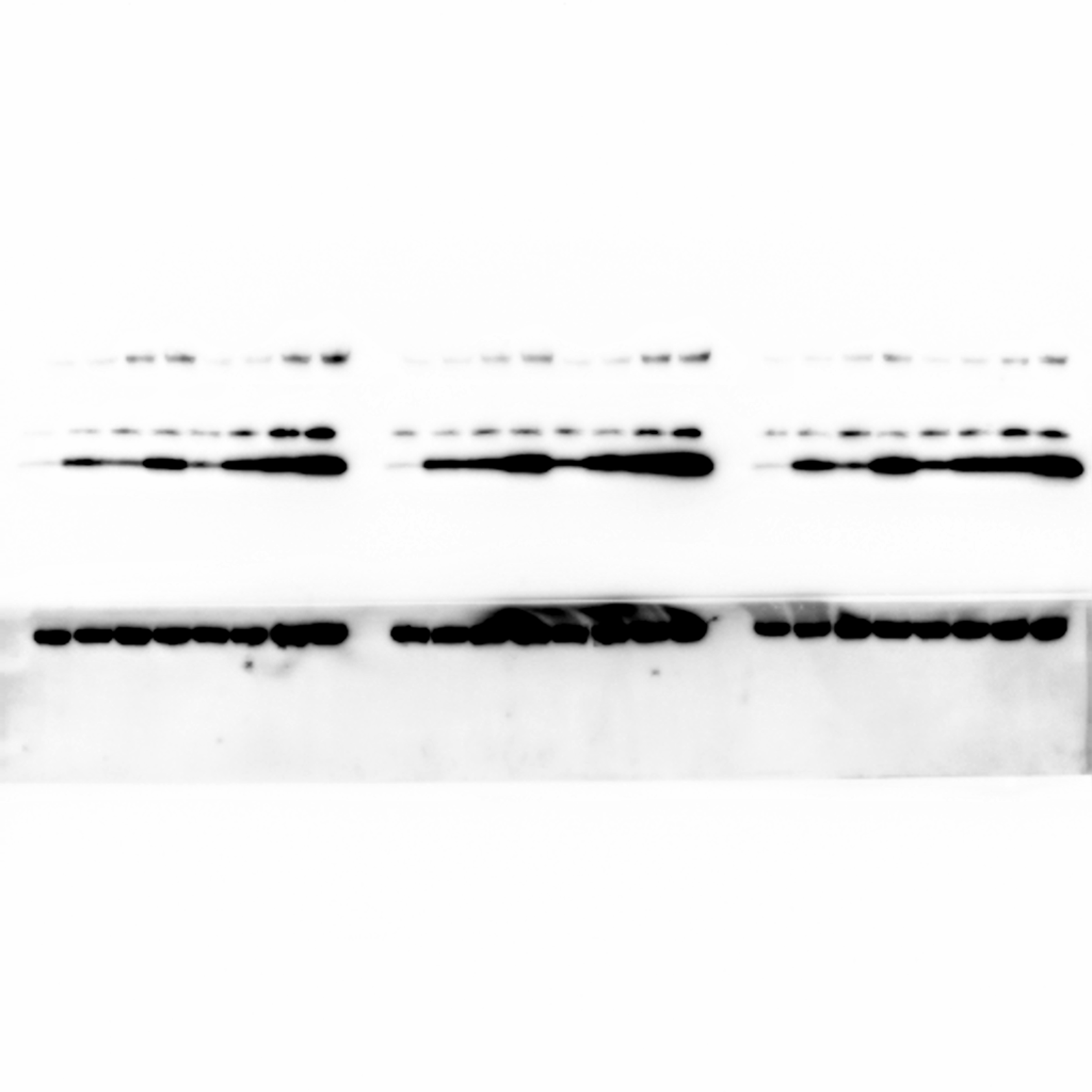

Supplement: Figure 2—source data 1. [file elife-98649-fig2-data1.zip › Figure 2-source data1/Figure 2C_LC3B_Etoposide_raw.tif]

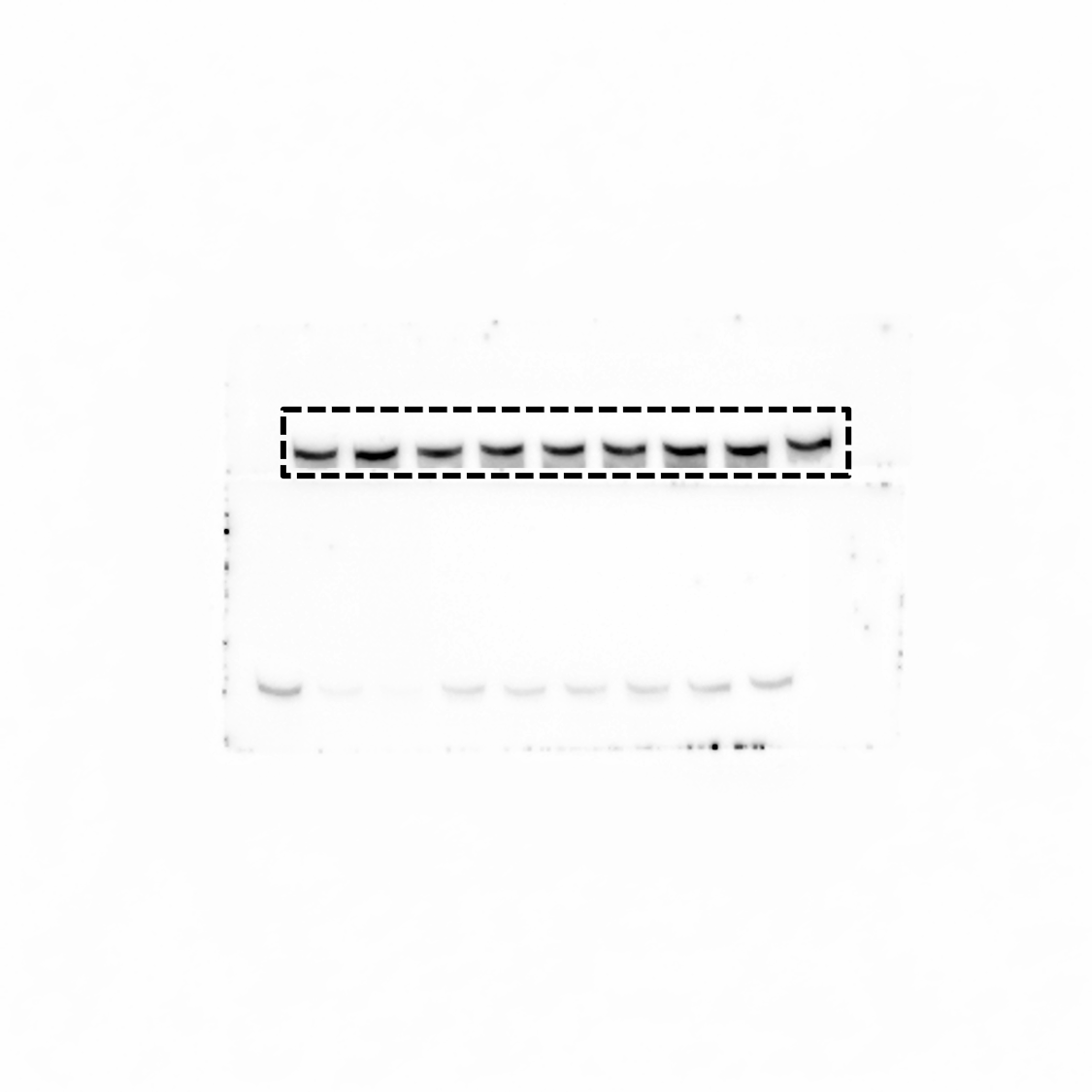

Supplement: Figure 3—source data 1. [file elife-98649-fig3-data1.zip › Figure 3-source data1/Figure 3A_mTOR_annotated.Tif]

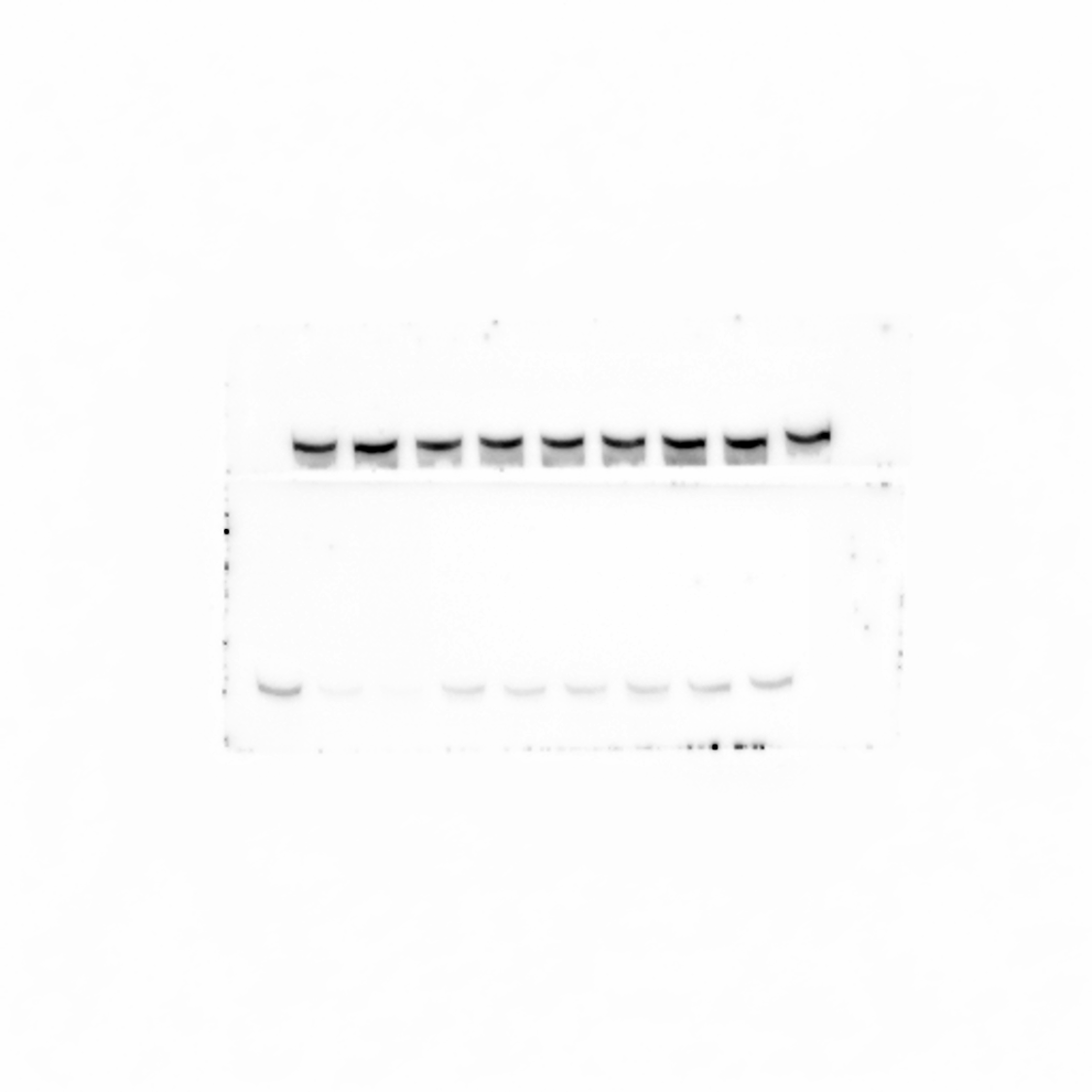

Supplement: Figure 3—source data 1. [file elife-98649-fig3-data1.zip › Figure 3-source data1/Figure 3A_mTOR_raw.Tif]

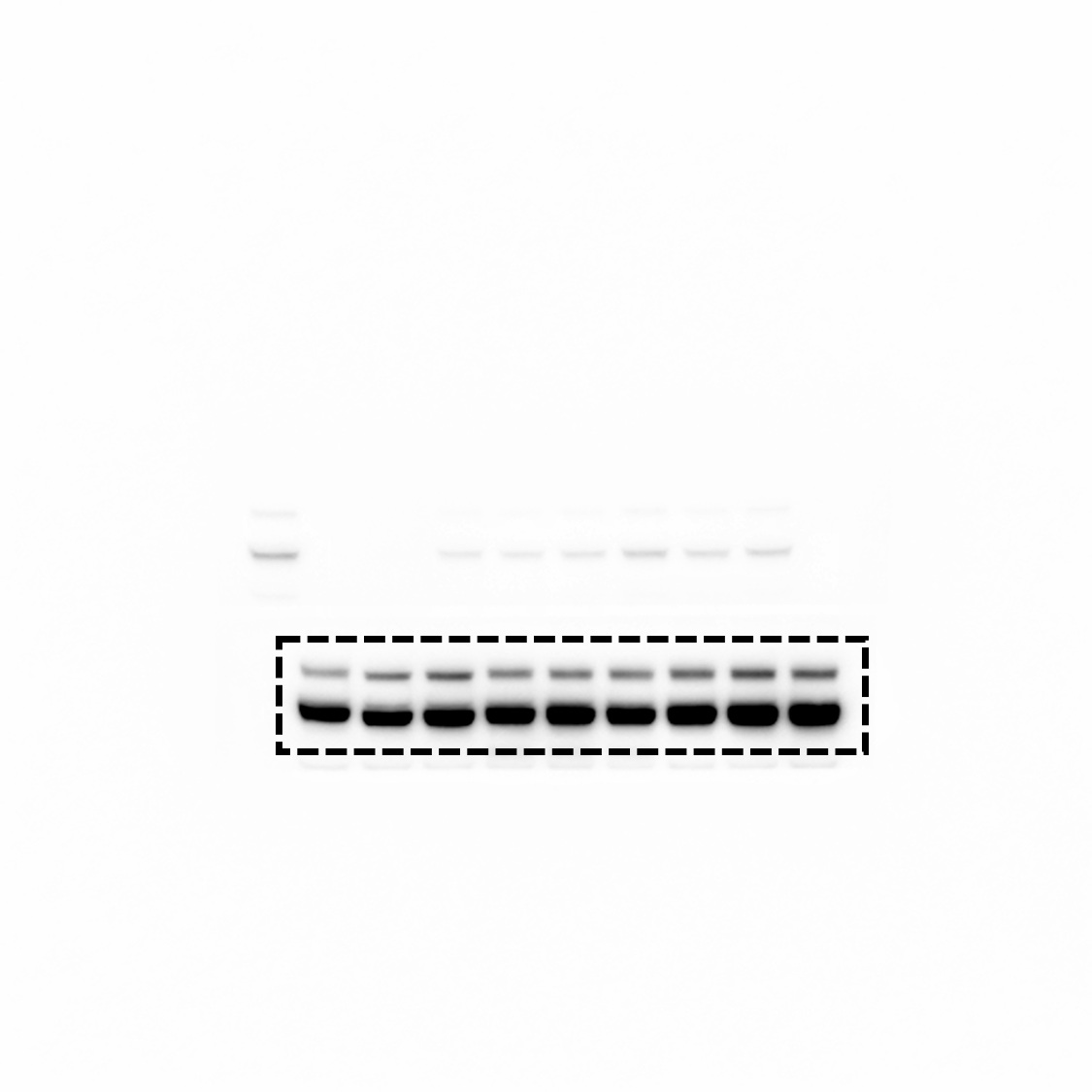

Supplement: Figure 3—source data 1. [file elife-98649-fig3-data1.zip › Figure 3-source data1/Figure 3A_p70S6K_annotated.Tif]

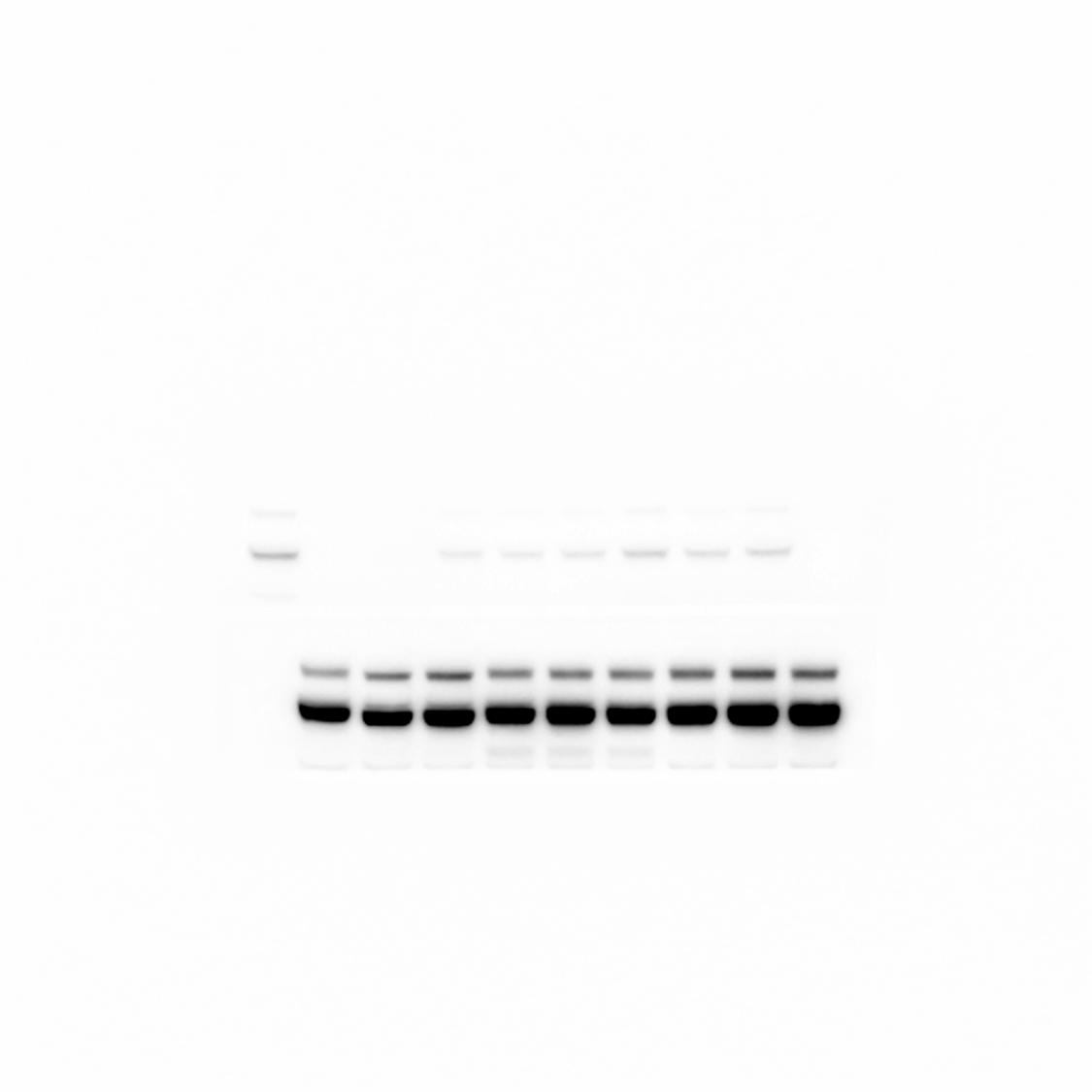

Supplement: Figure 3—source data 1. [file elife-98649-fig3-data1.zip › Figure 3-source data1/Figure 3A_p70S6K_raw.Tif]

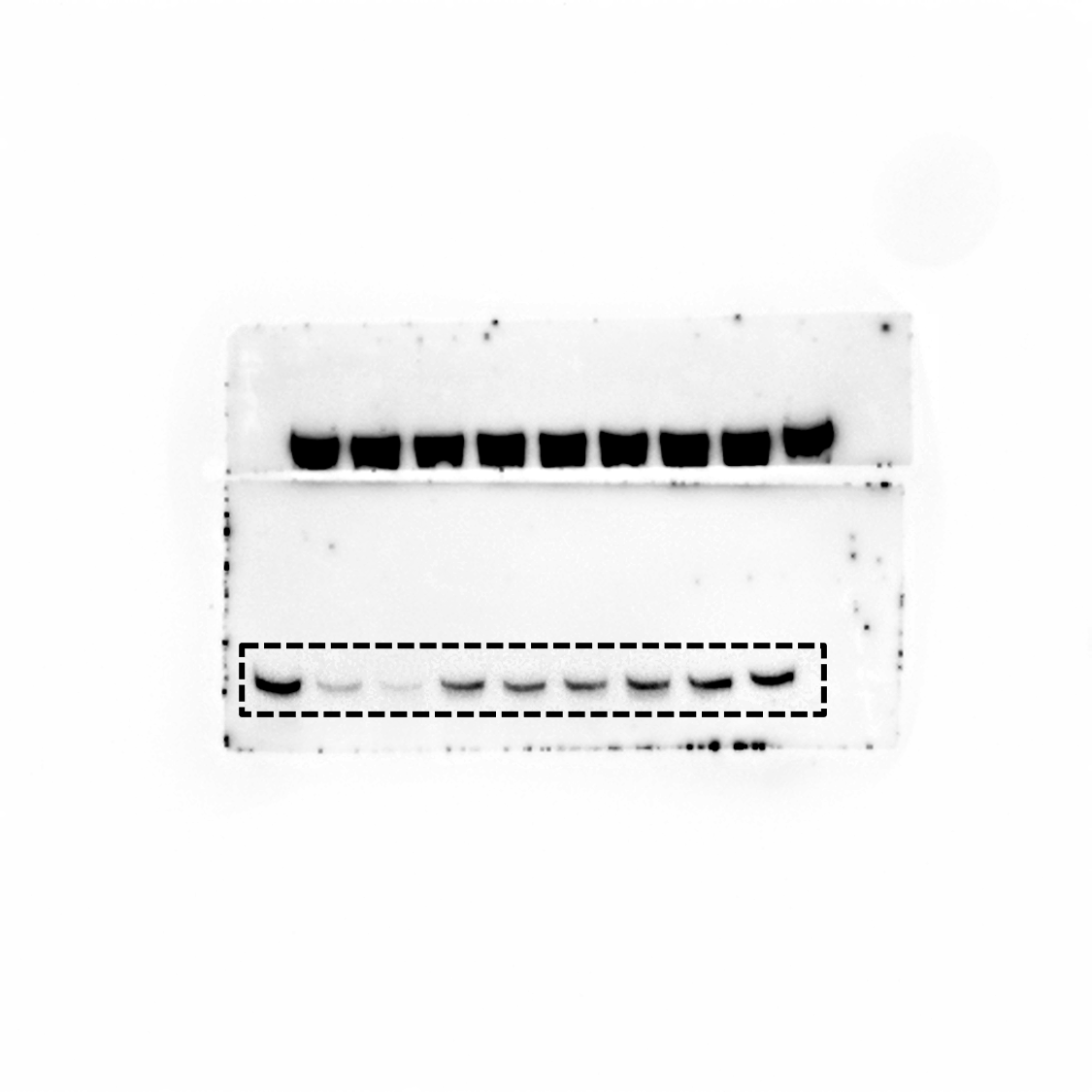

Supplement: Figure 3—source data 1. [file elife-98649-fig3-data1.zip › Figure 3-source data1/Figure 3A_p-mTOR_annotated.Tif]

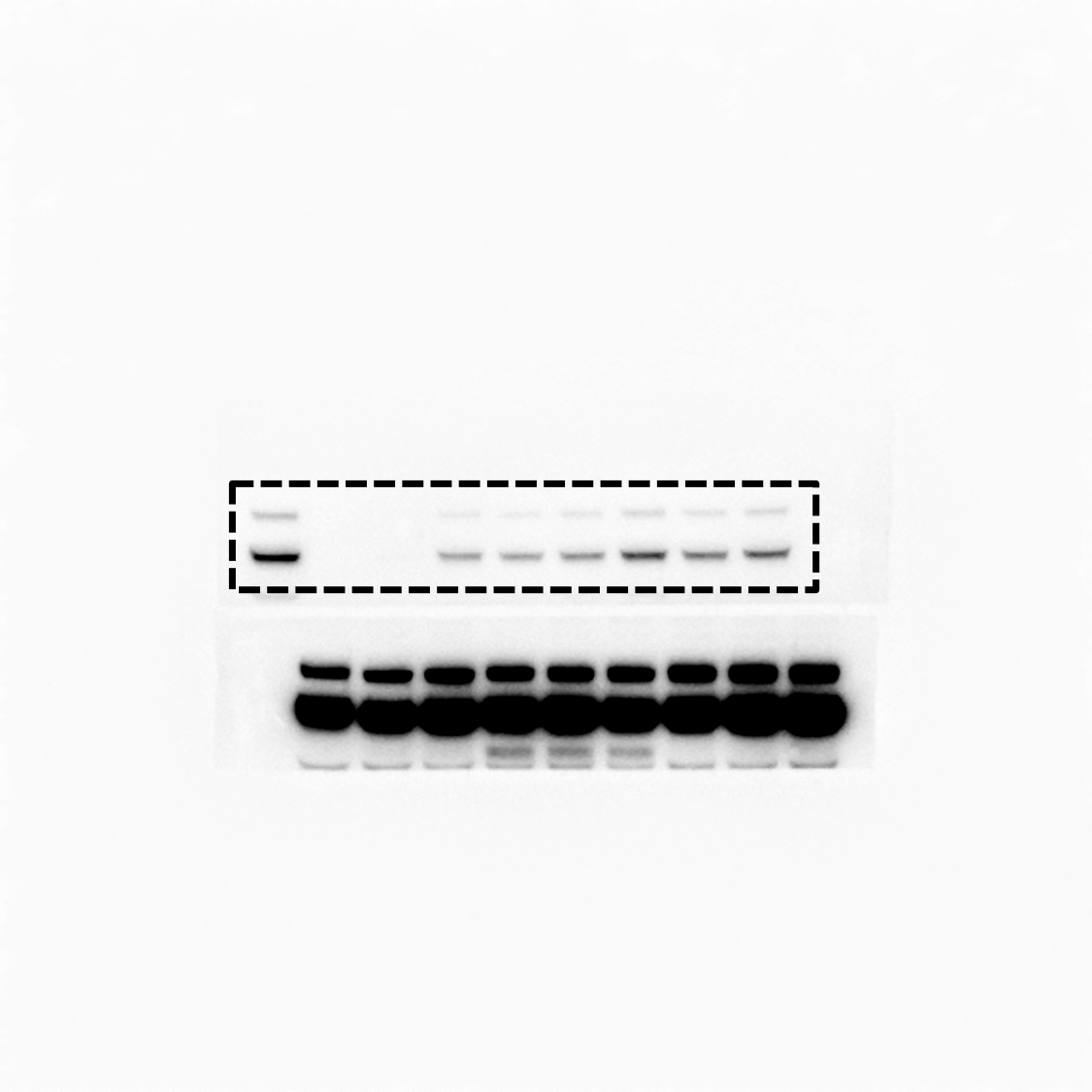

Supplement: Figure 3—source data 1. [file elife-98649-fig3-data1.zip › Figure 3-source data1/Figure 3A_p-p70S6K_annotated.Tif]

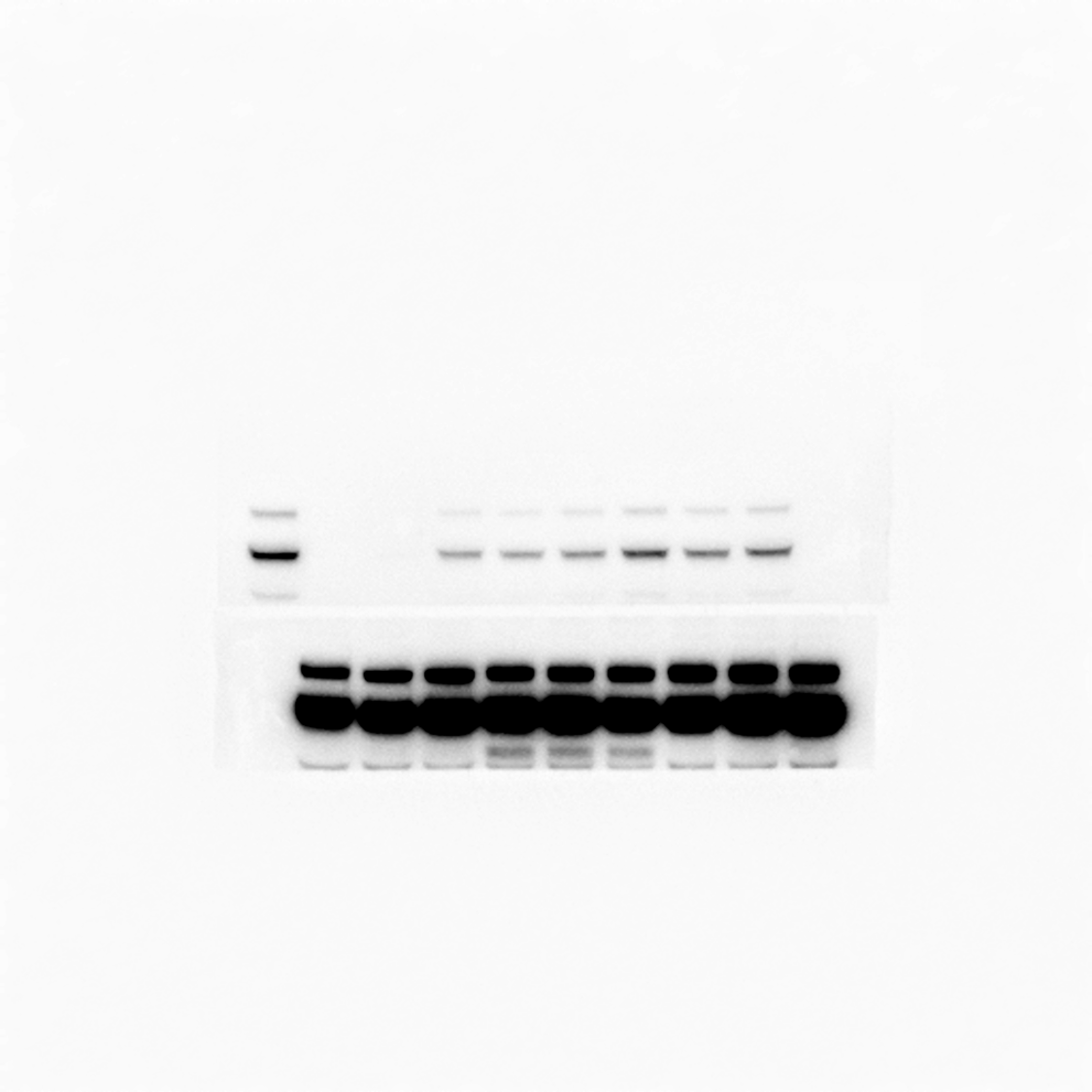

Supplement: Figure 3—source data 1. [file elife-98649-fig3-data1.zip › Figure 3-source data1/Figure 3A_p-p70S6K_raw.Tif]

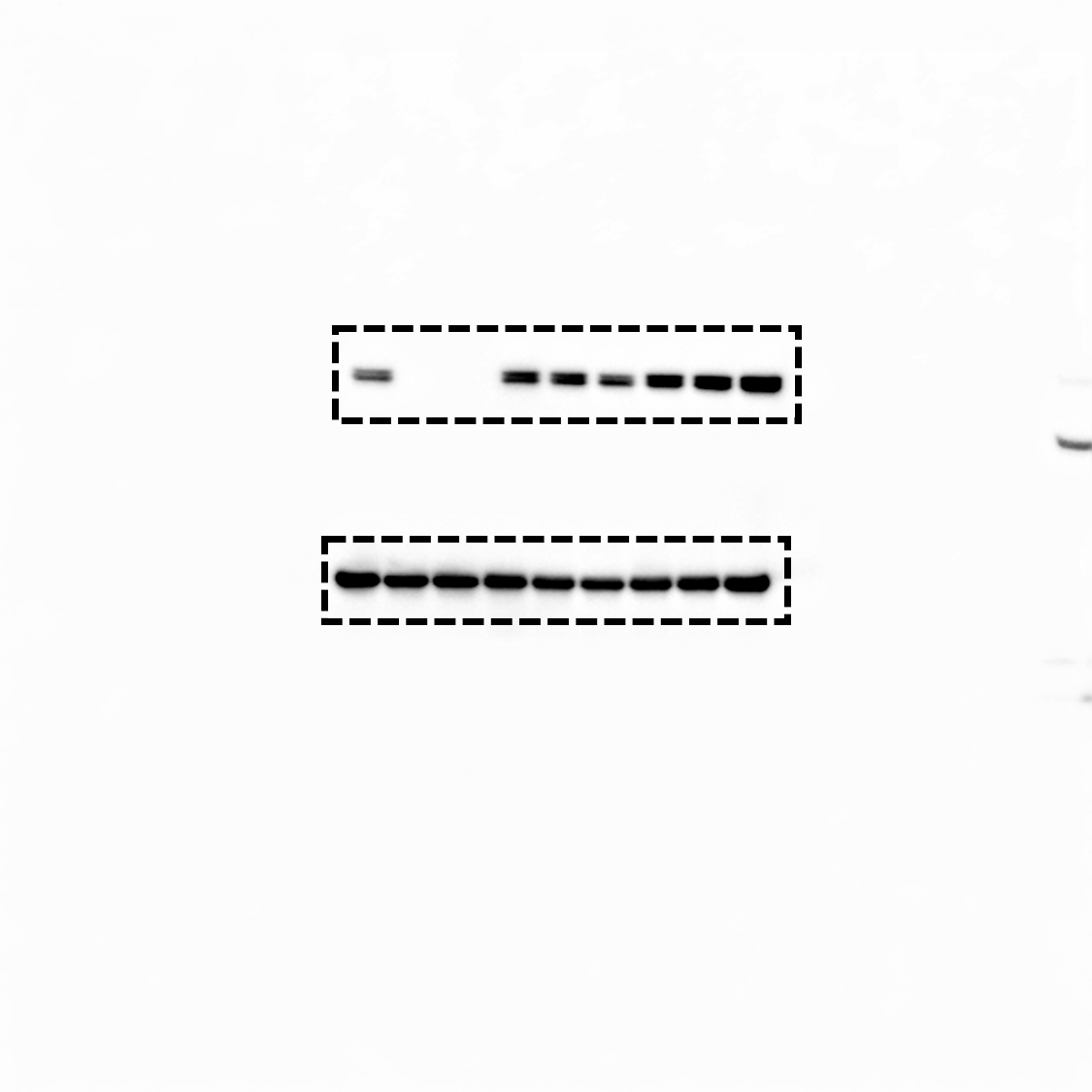

Supplement: Figure 3—source data 1. [file elife-98649-fig3-data1.zip › Figure 3-source data1/Figure 3A_p-S6_S6_annotated.Tif]

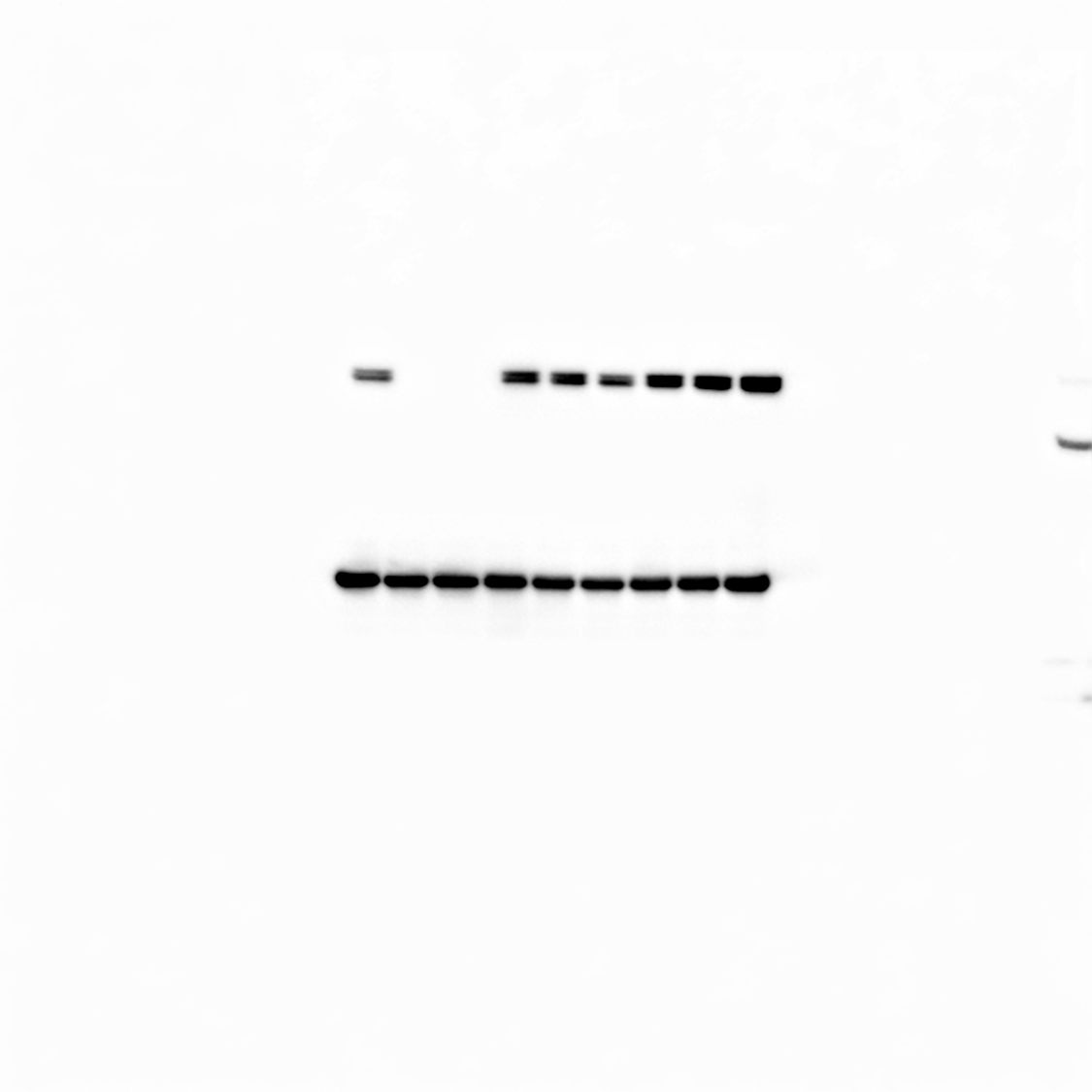

Supplement: Figure 3—source data 1. [file elife-98649-fig3-data1.zip › Figure 3-source data1/Figure 3A_p-S6_S6_raw.Tif]

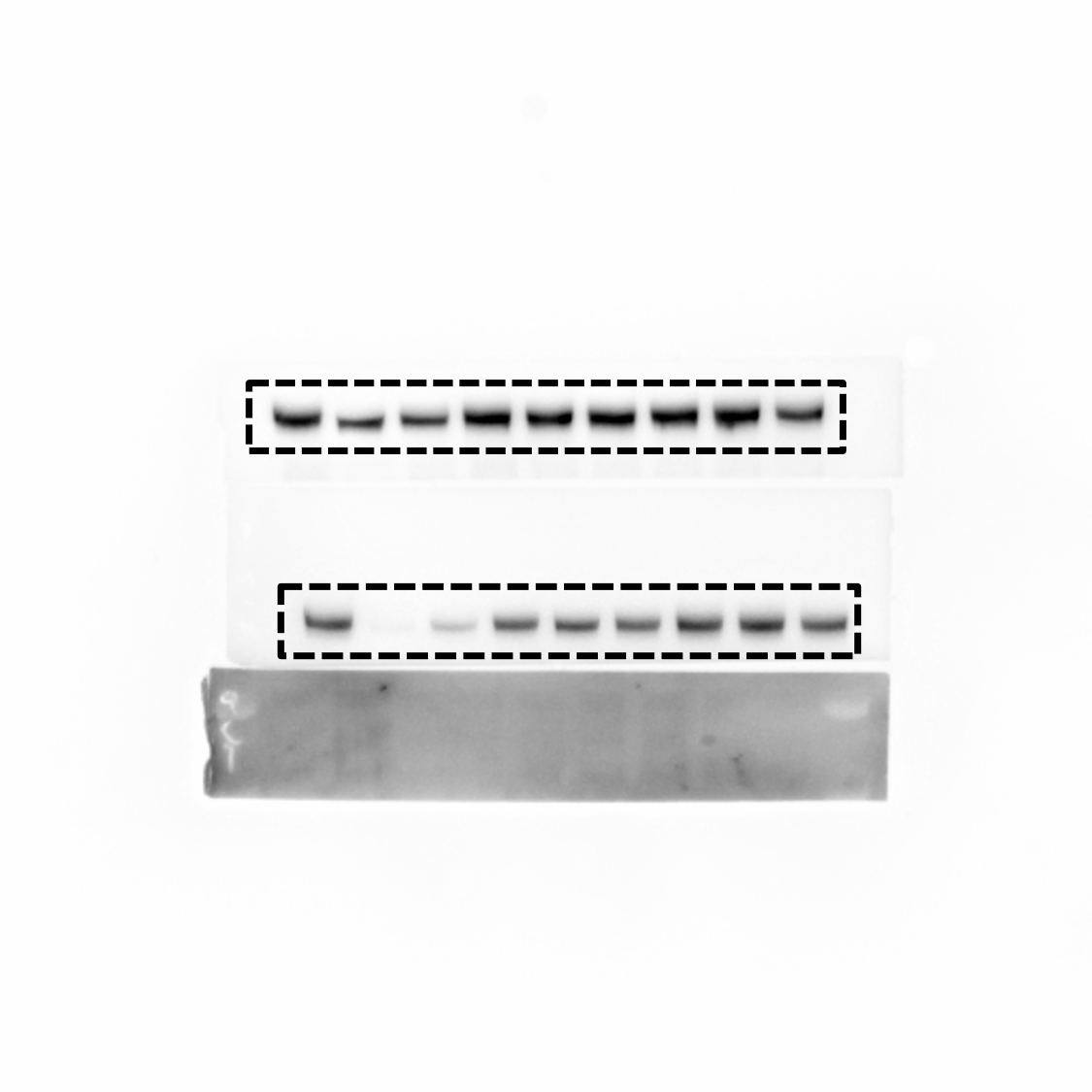

Supplement: Figure 3—source data 1. [file elife-98649-fig3-data1.zip › Figure 3-source data1/Figure 3A_p-ULK1_ULK1_annotated.Tif]

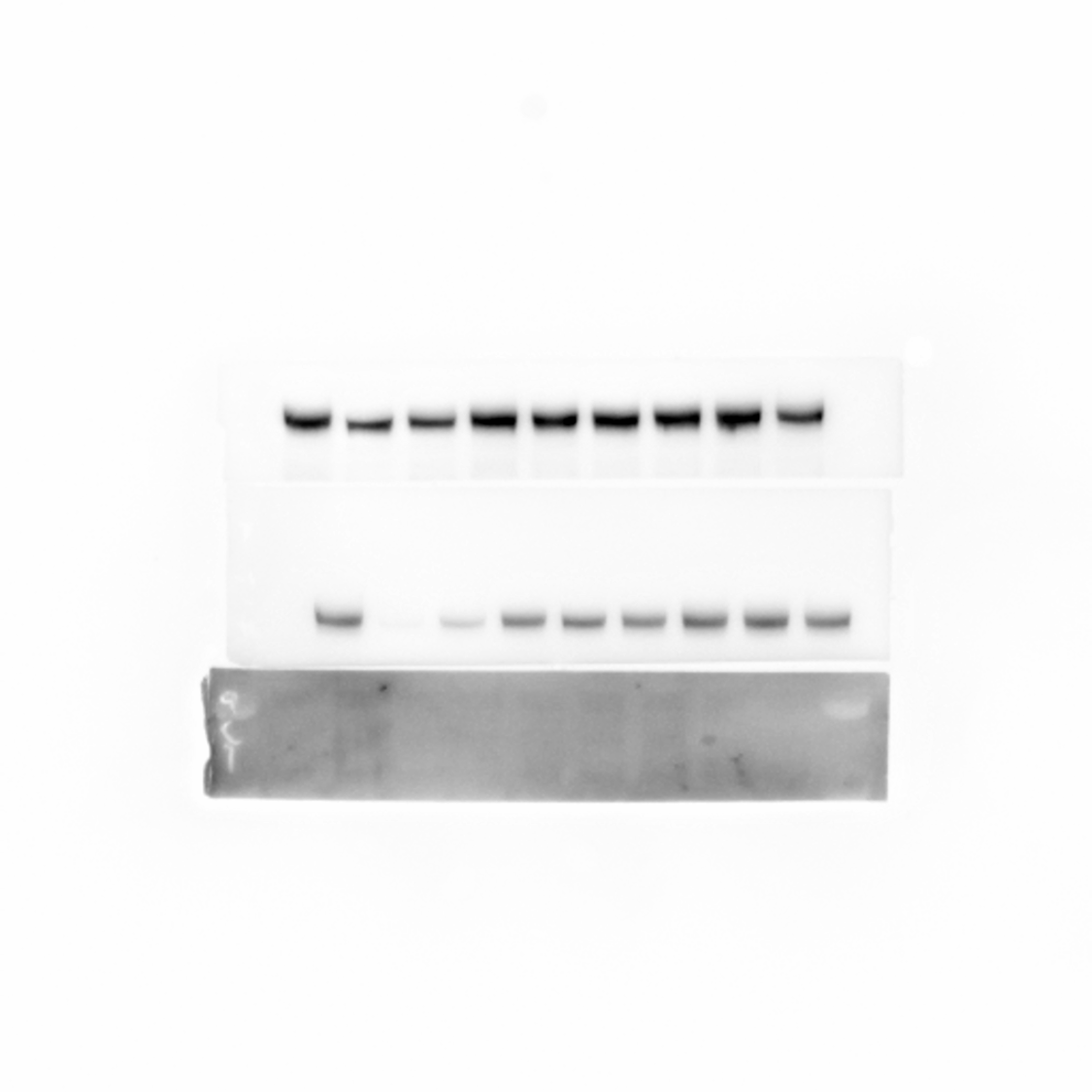

Supplement: Figure 3—source data 1. [file elife-98649-fig3-data1.zip › Figure 3-source data1/Figure 3A_p-ULK1_ULK1_raw.Tif]

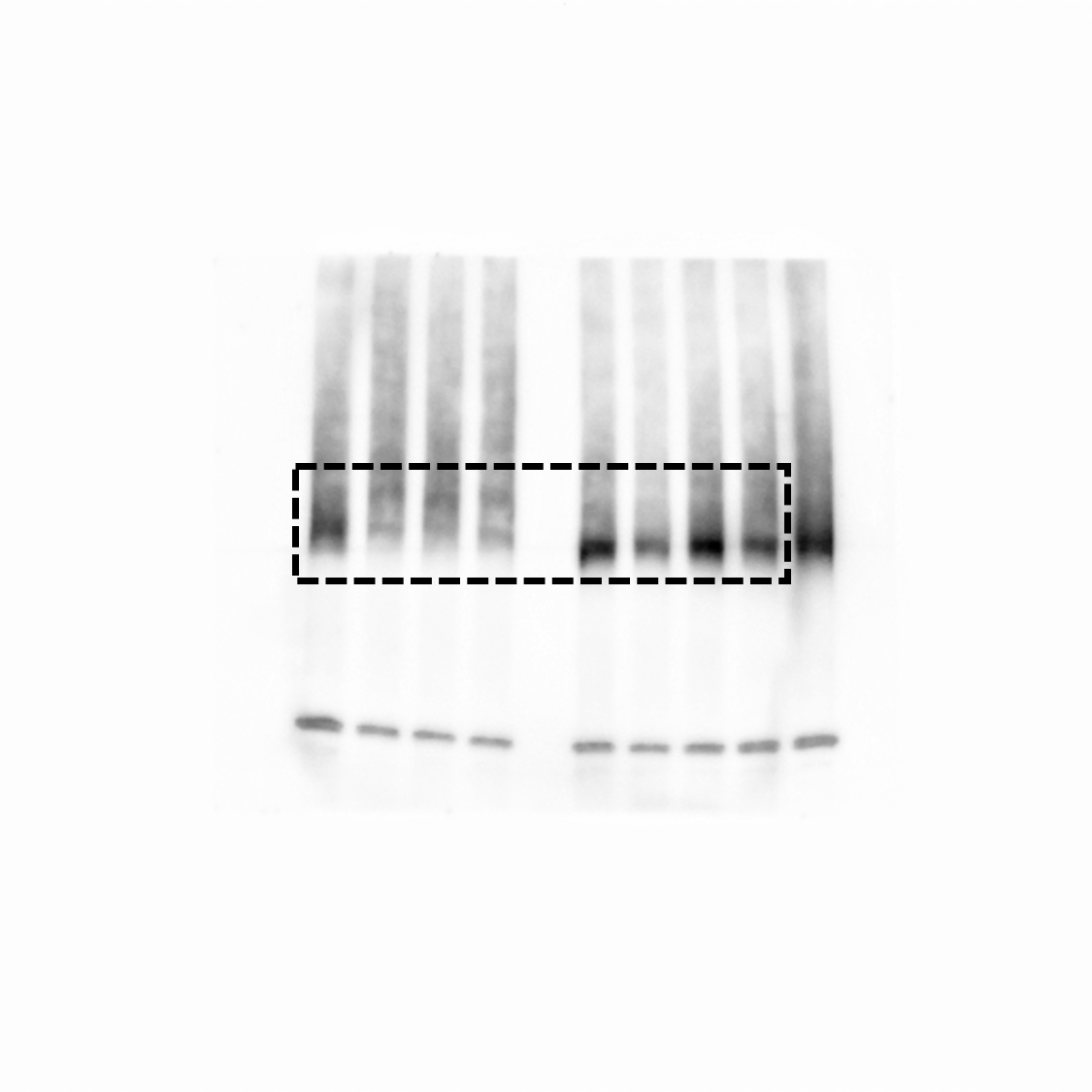

Supplement: Figure 5—source data 1. [file elife-98649-fig5-data1.zip › Figure 5-source data1/Figure 5D phostagPAGE_JIP4_annotated.tif]

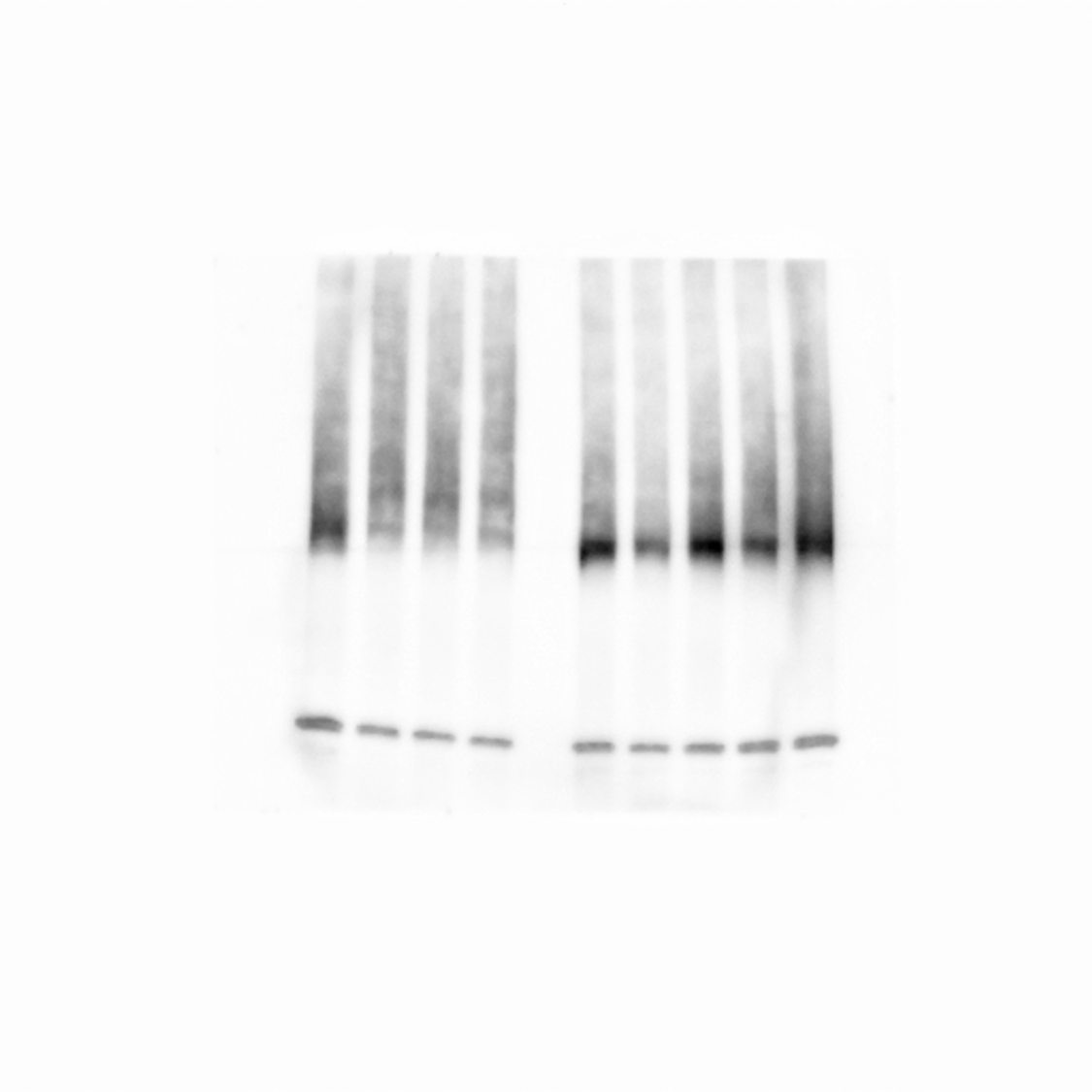

Supplement: Figure 5—source data 1. [file elife-98649-fig5-data1.zip › Figure 5-source data1/Figure 5D phostagPAGE_JIP4_raw.tif]

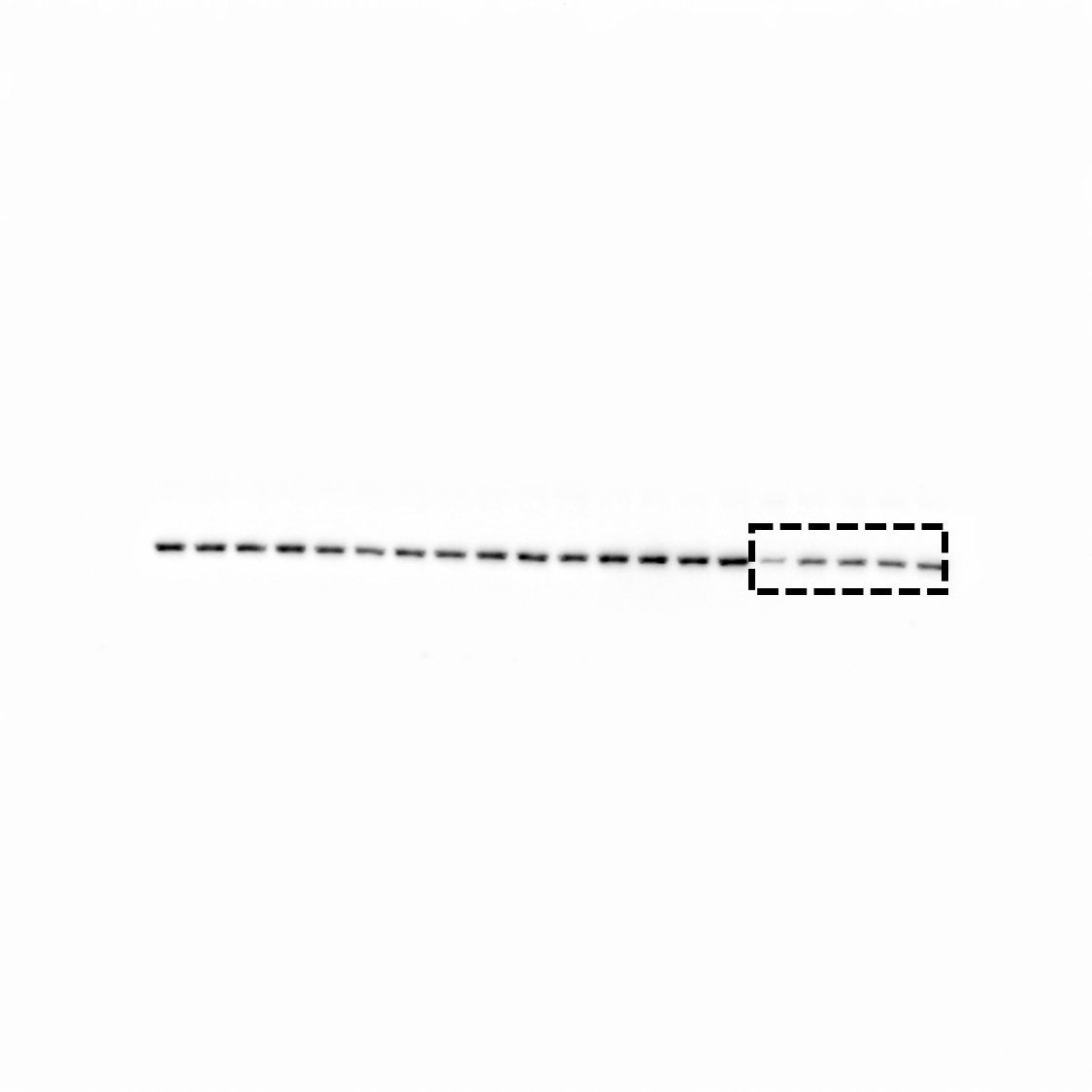

Supplement: Figure 5—source data 1. [file elife-98649-fig5-data1.zip › Figure 5-source data1/Figure 5G_actin_annotated.tif]

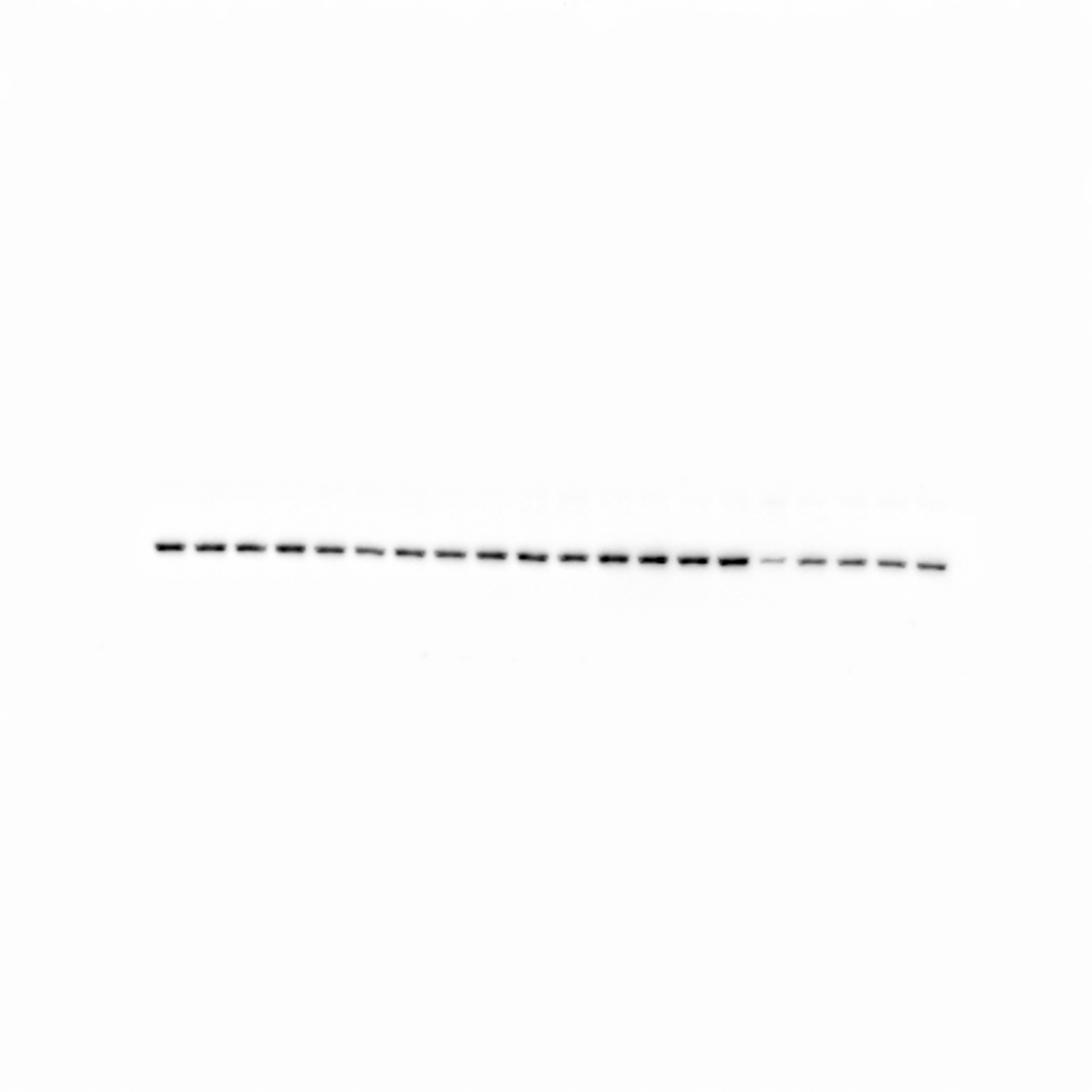

Supplement: Figure 5—source data 1. [file elife-98649-fig5-data1.zip › Figure 5-source data1/Figure 5G_actin_raw.tif]

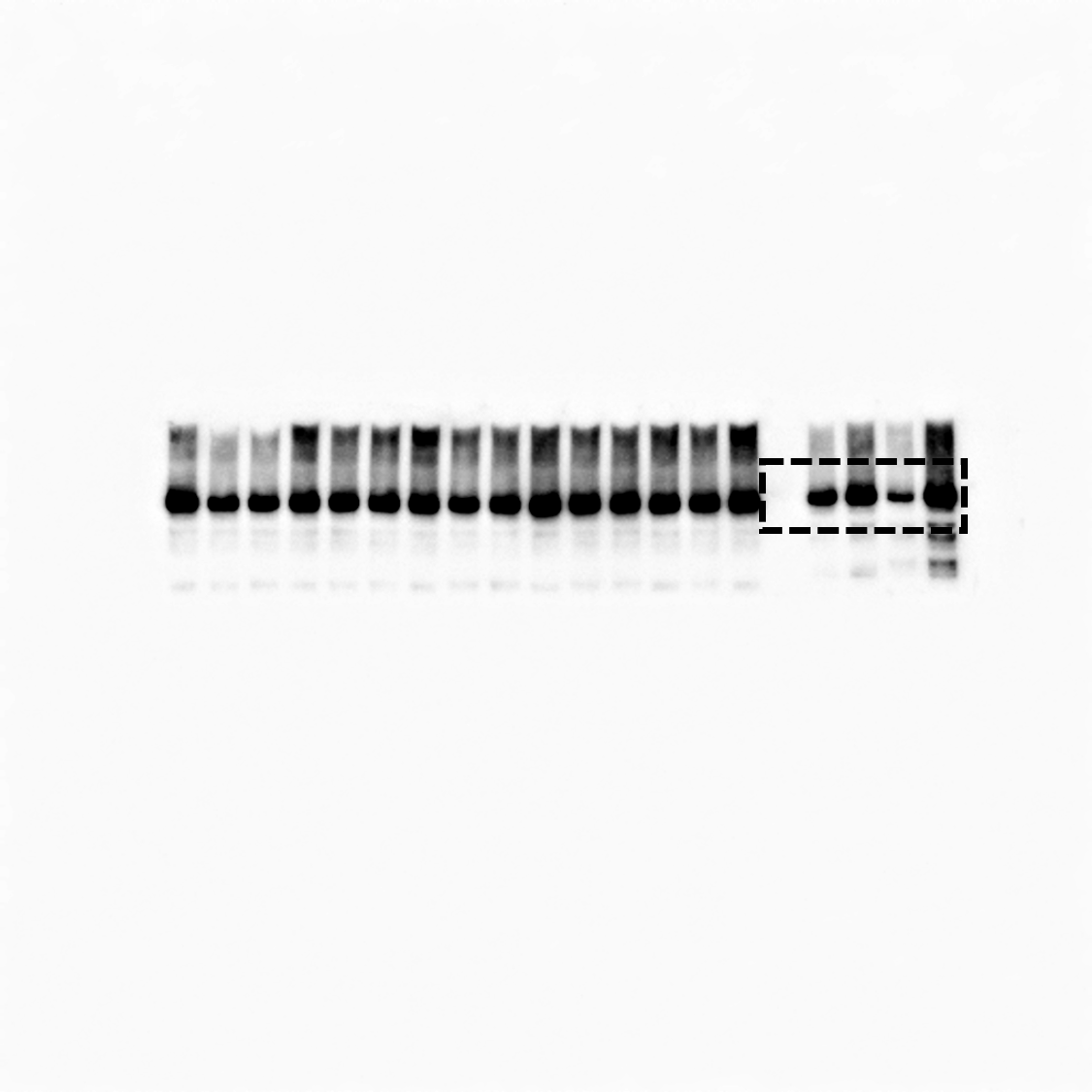

Supplement: Figure 5—source data 1. [file elife-98649-fig5-data1.zip › Figure 5-source data1/Figure 5G_JIP4_annotated.tif]

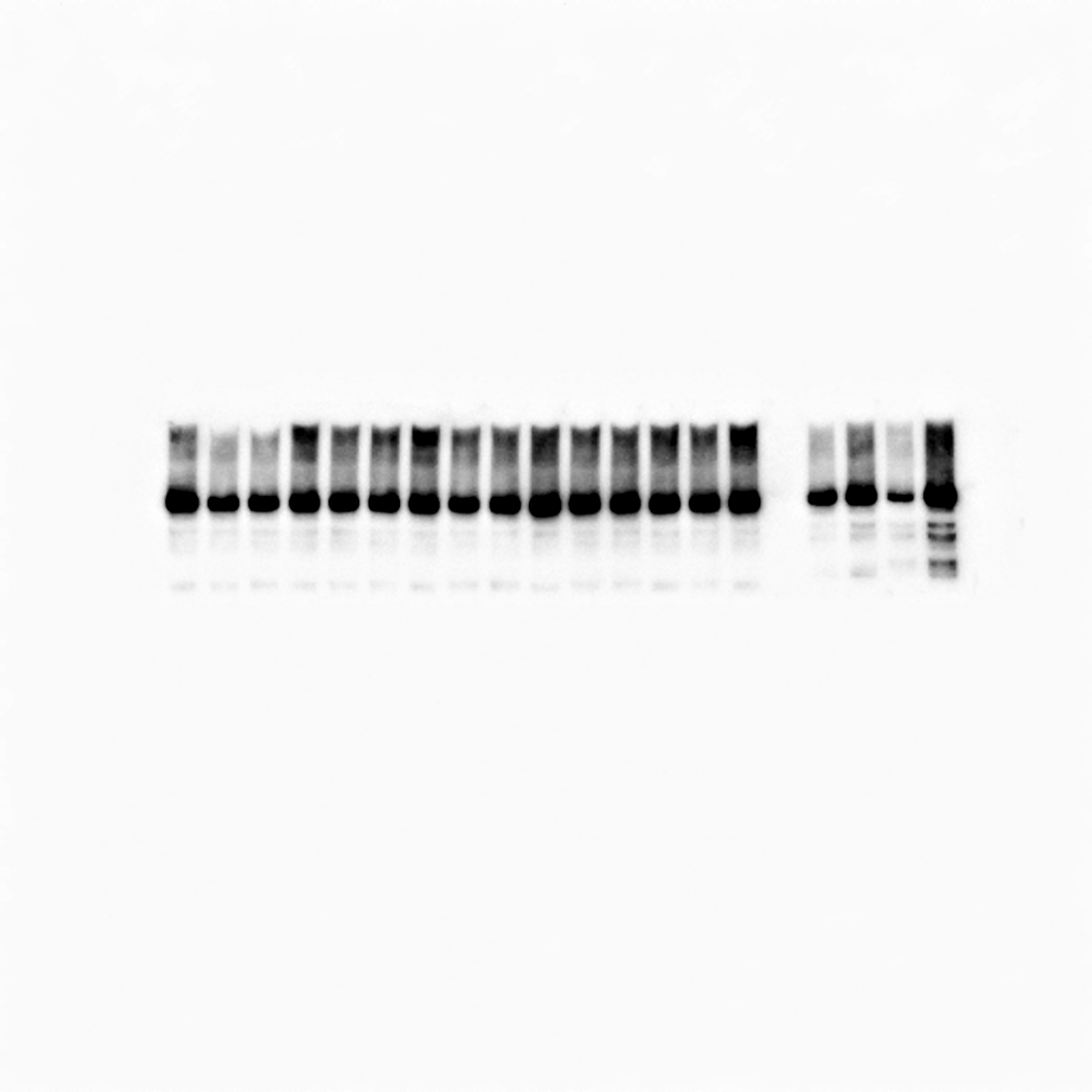

Supplement: Figure 5—source data 1. [file elife-98649-fig5-data1.zip › Figure 5-source data1/Figure 5G_JIP4_raw.tif]

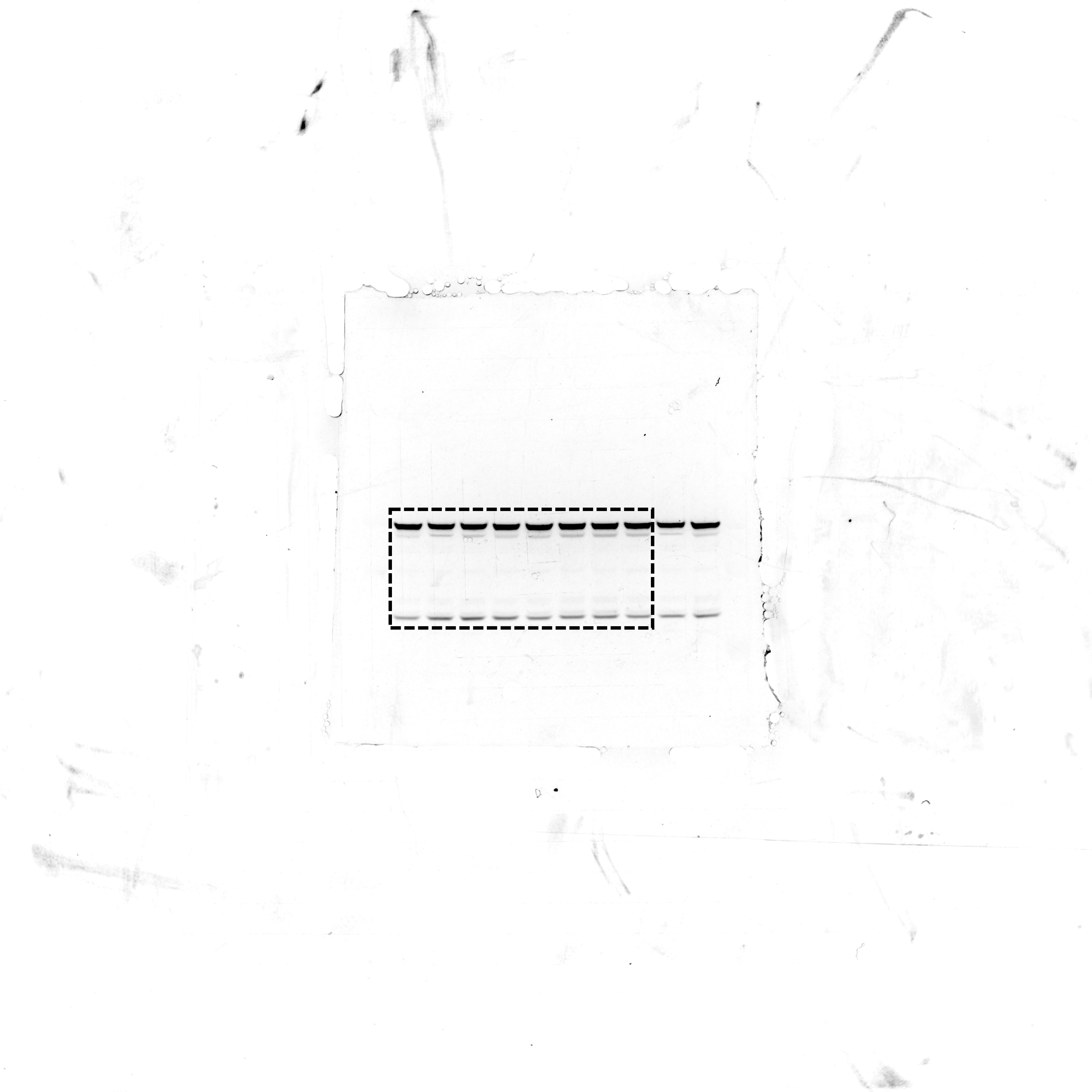

Supplement: Figure 6—source data 1. [file elife-98649-fig6-data1.zip › Figure 6-source data1/Figure 6A_ gelfluorescence_benzmimdazole_annotated.Tif]

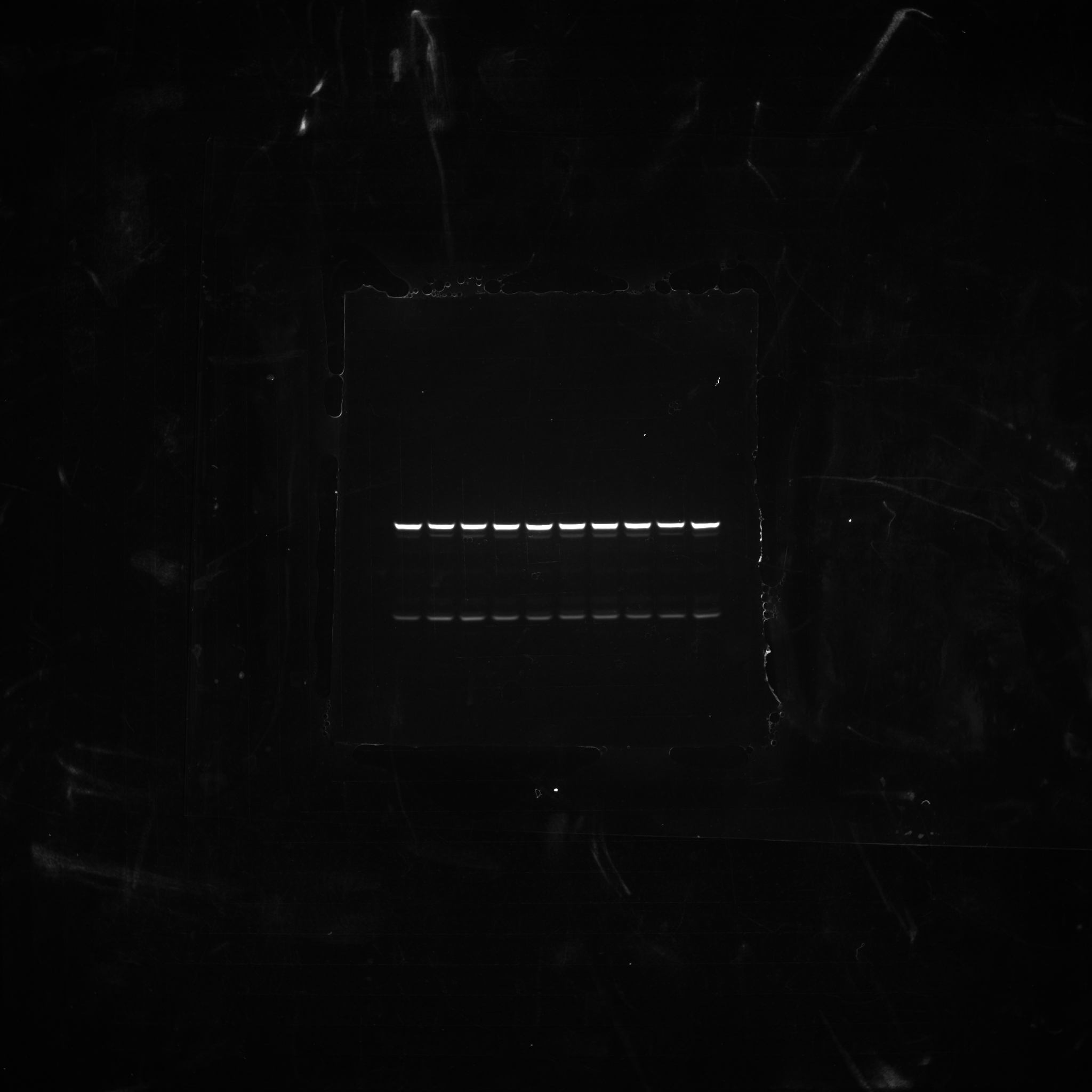

Supplement: Figure 6—source data 1. [file elife-98649-fig6-data1.zip › Figure 6-source data1/Figure 6A_ gelfluorescence_benzmimdazole_raw.Tif]

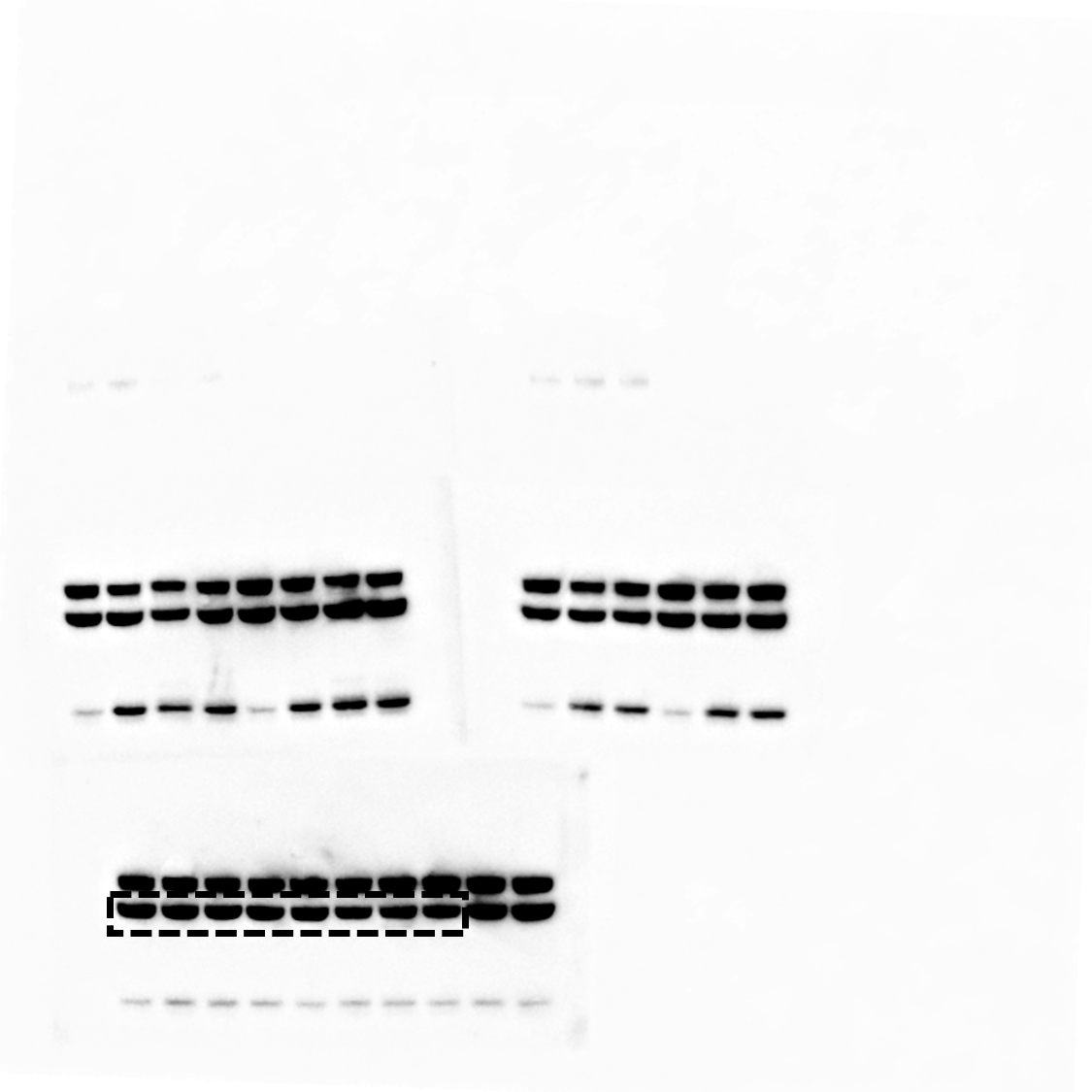

Supplement: Figure 6—source data 1. [file elife-98649-fig6-data1.zip › Figure 6-source data1/Figure 6A_actin_ benzimidazole_annotated.tif]

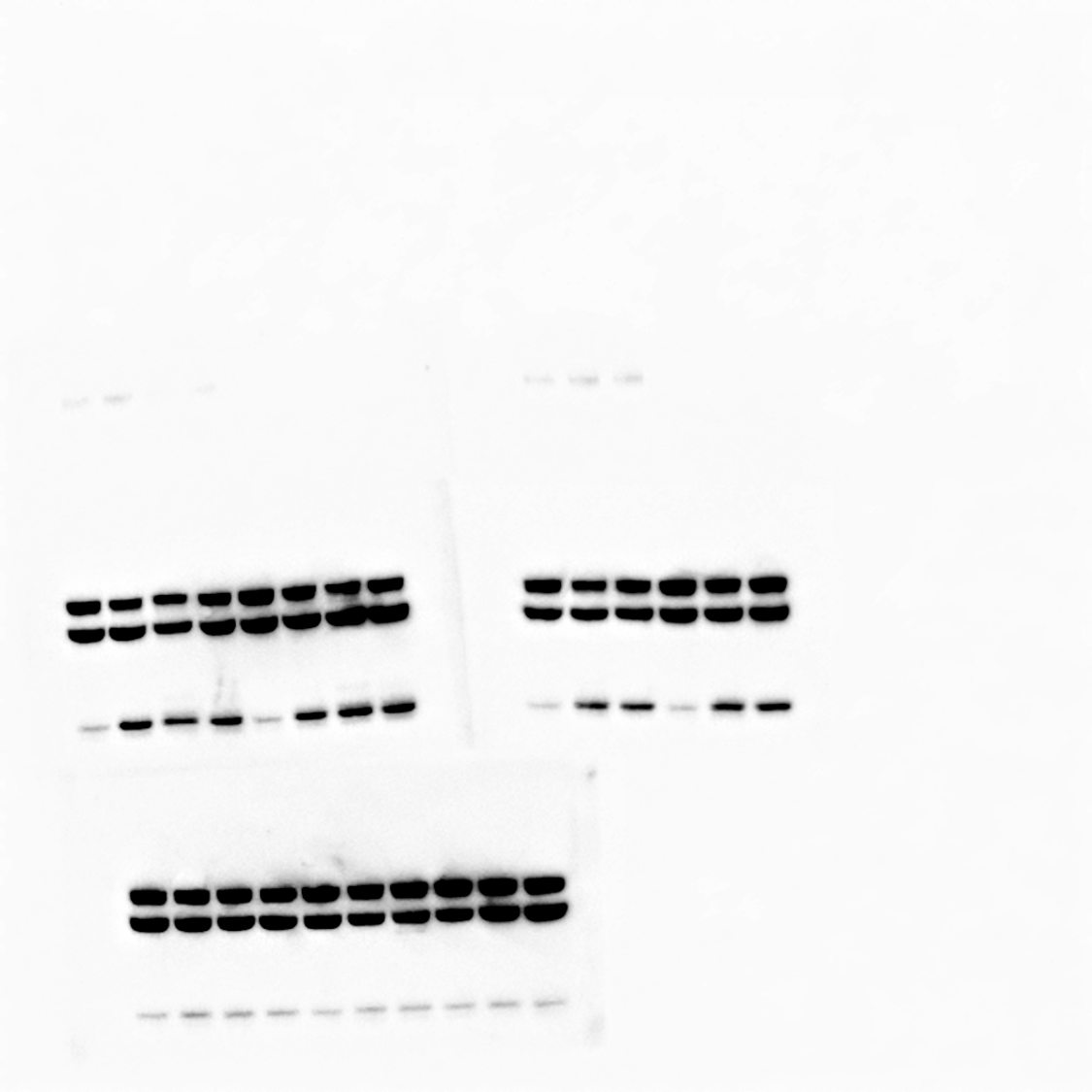

Supplement: Figure 6—source data 1. [file elife-98649-fig6-data1.zip › Figure 6-source data1/Figure 6A_actin_ benzimidazole_raw.tif]

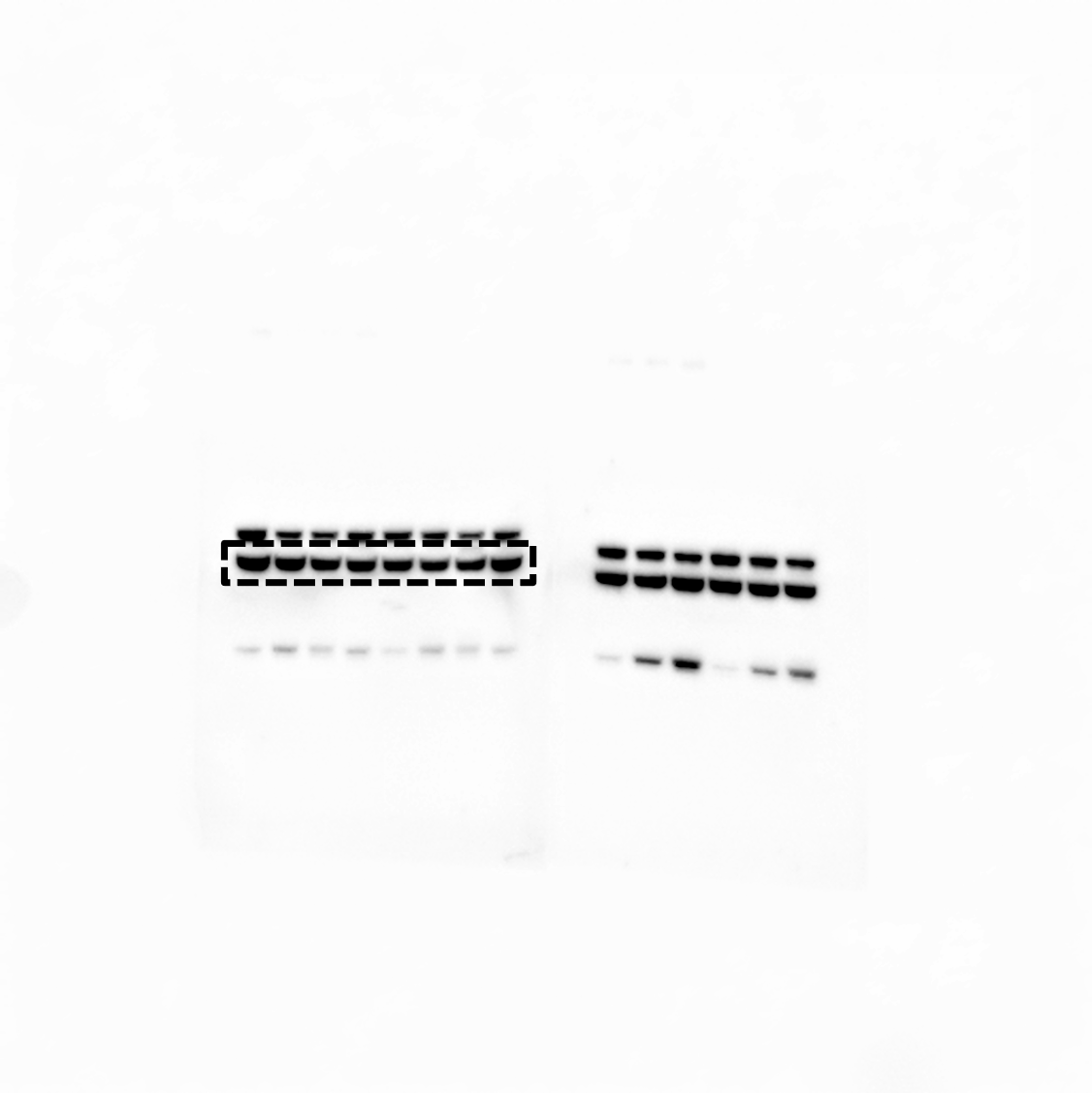

Supplement: Figure 6—source data 1. [file elife-98649-fig6-data1.zip › Figure 6-source data1/Figure 6A_actin_Topoisomerasei _annotated.tif]

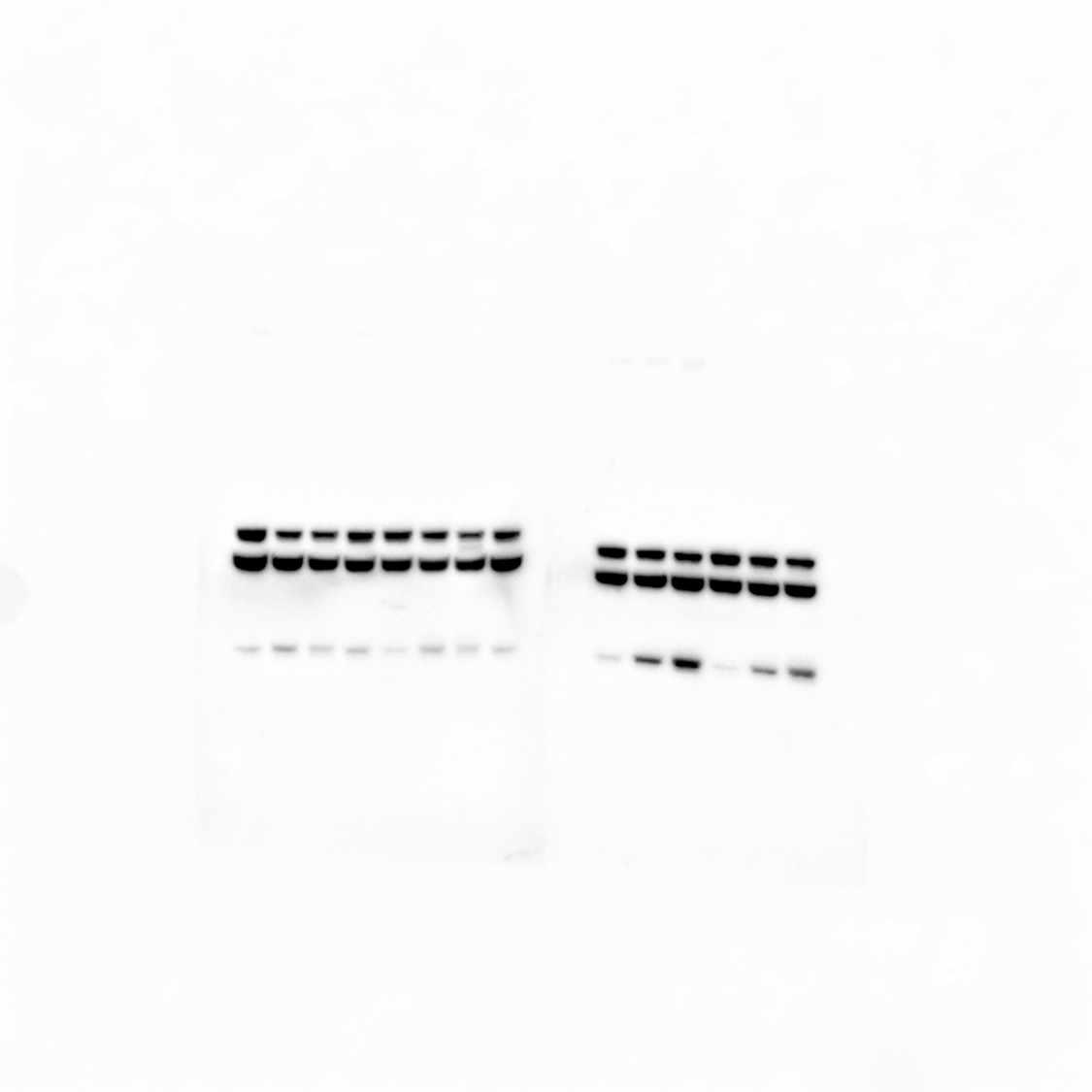

Supplement: Figure 6—source data 1. [file elife-98649-fig6-data1.zip › Figure 6-source data1/Figure 6A_actin_Topoisomerasei _raw.tif]

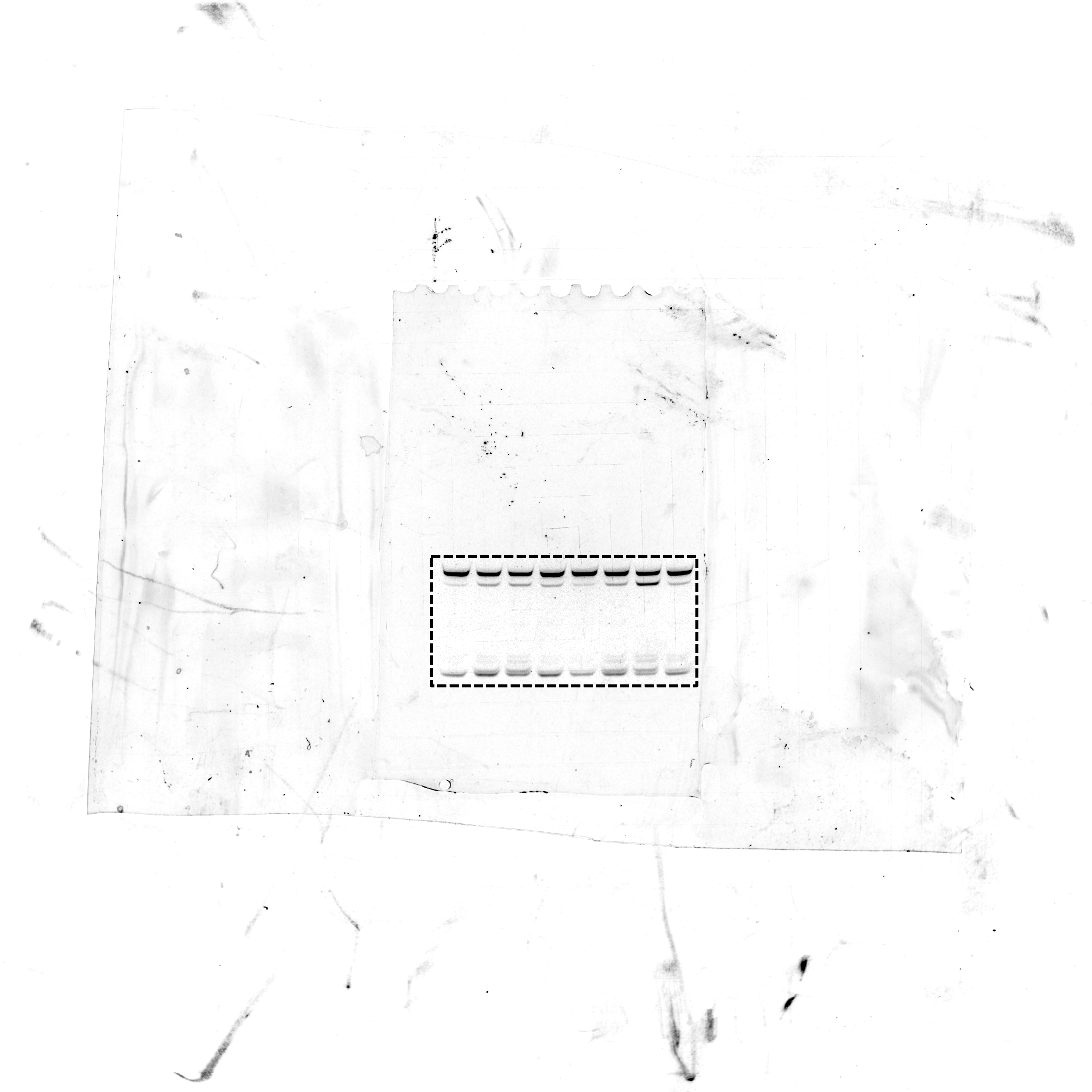

Supplement: Figure 6—source data 1. [file elife-98649-fig6-data1.zip › Figure 6-source data1/Figure 6A_gelfluorescence_Topoisomerasei_annotated.Tif]

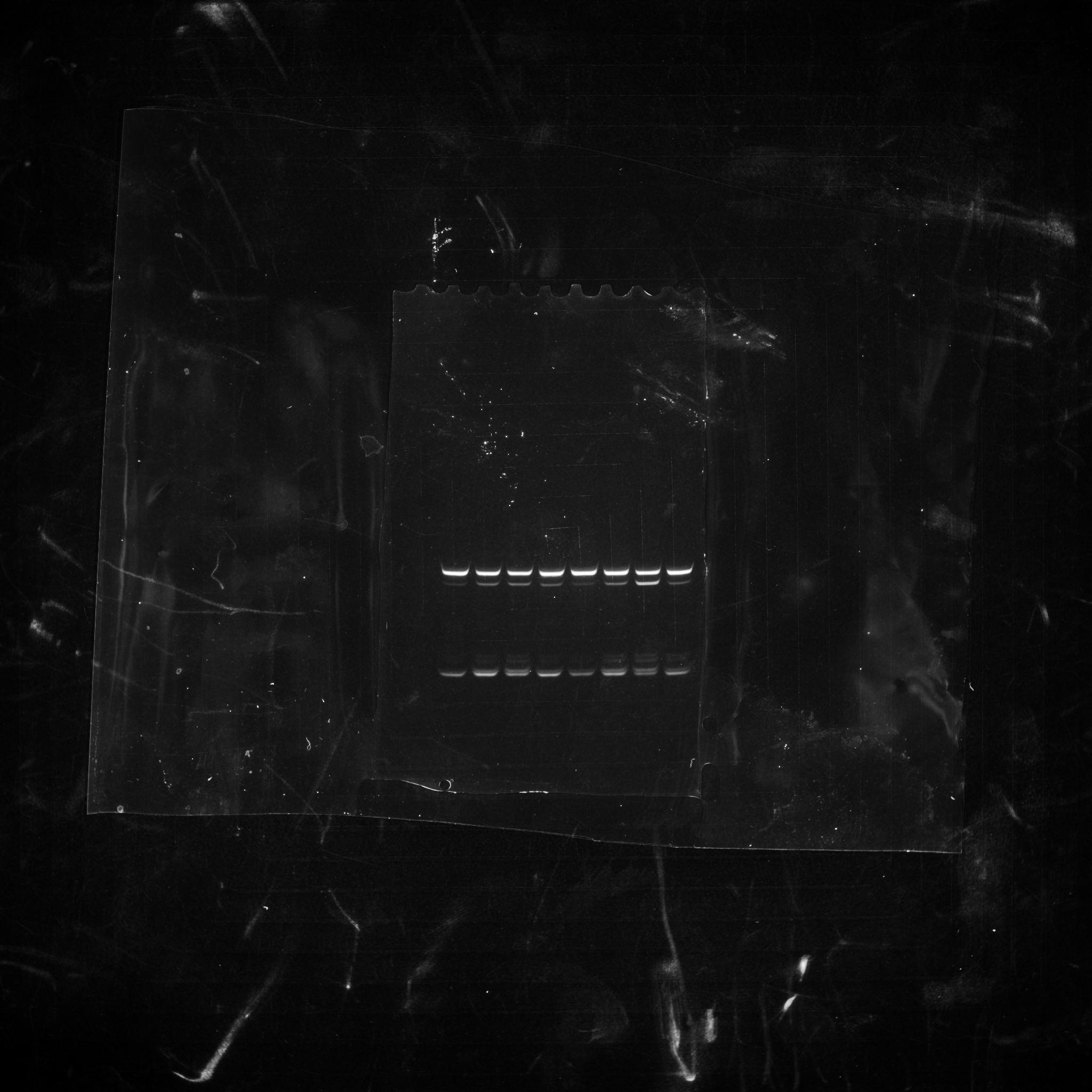

Supplement: Figure 6—source data 1. [file elife-98649-fig6-data1.zip › Figure 6-source data1/Figure 6A_gelfluorescence_Topoisomerasei_raw.Tif]

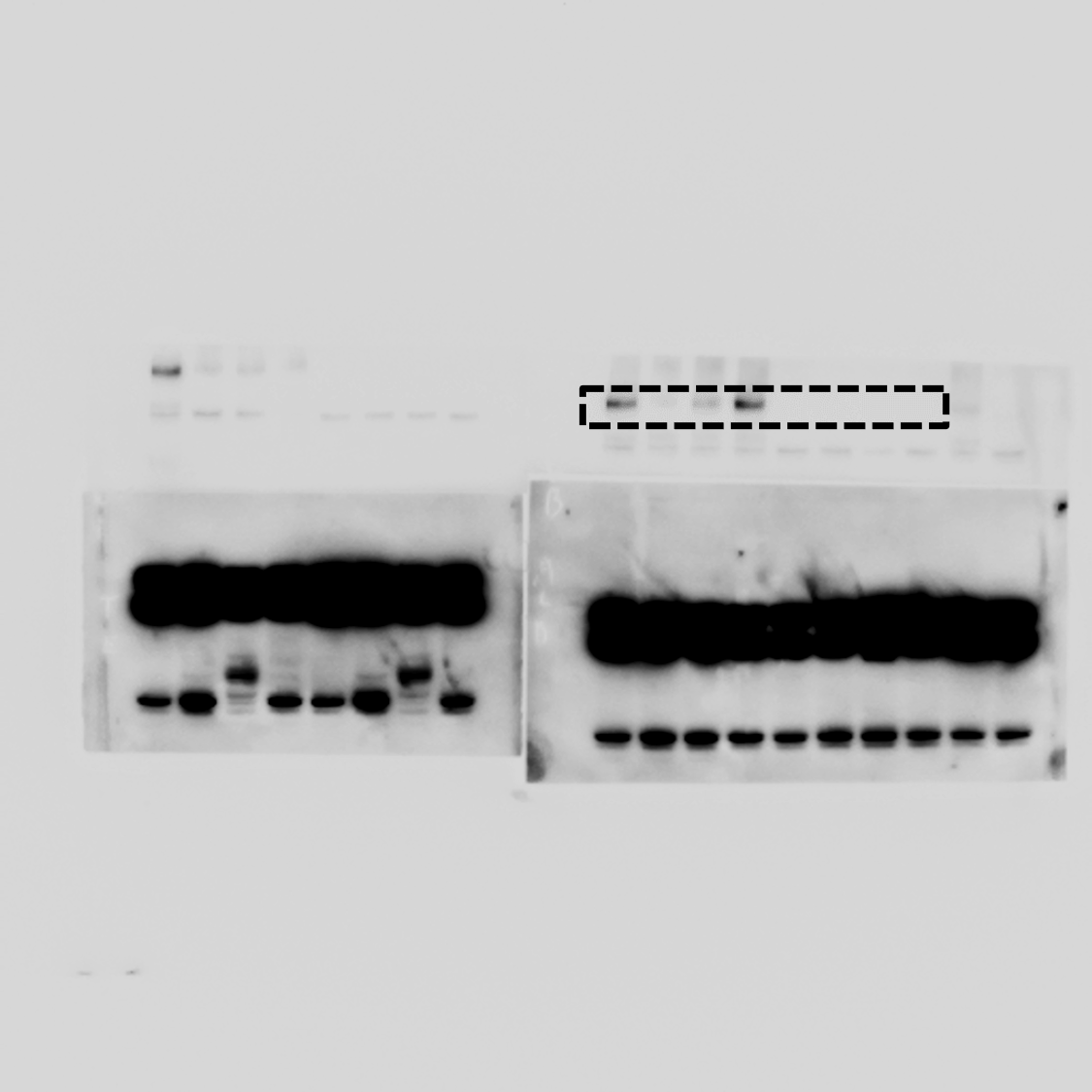

Supplement: Figure 6—source data 1. [file elife-98649-fig6-data1.zip › Figure 6-source data1/Figure 6A_JIP4_ benzimidazole_annotated.tif]

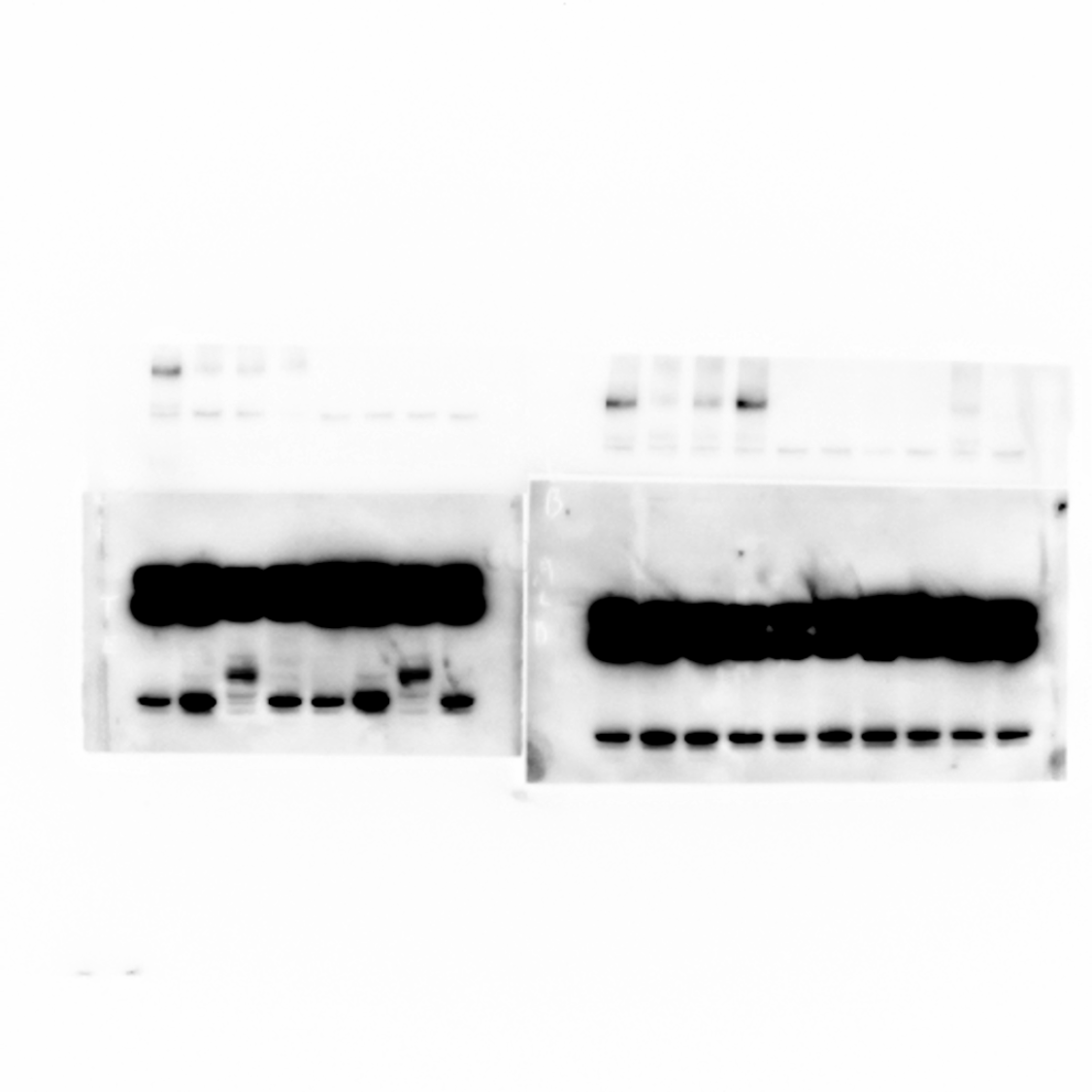

Supplement: Figure 6—source data 1. [file elife-98649-fig6-data1.zip › Figure 6-source data1/Figure 6A_JIP4_ benzimidazole_raw.tif]

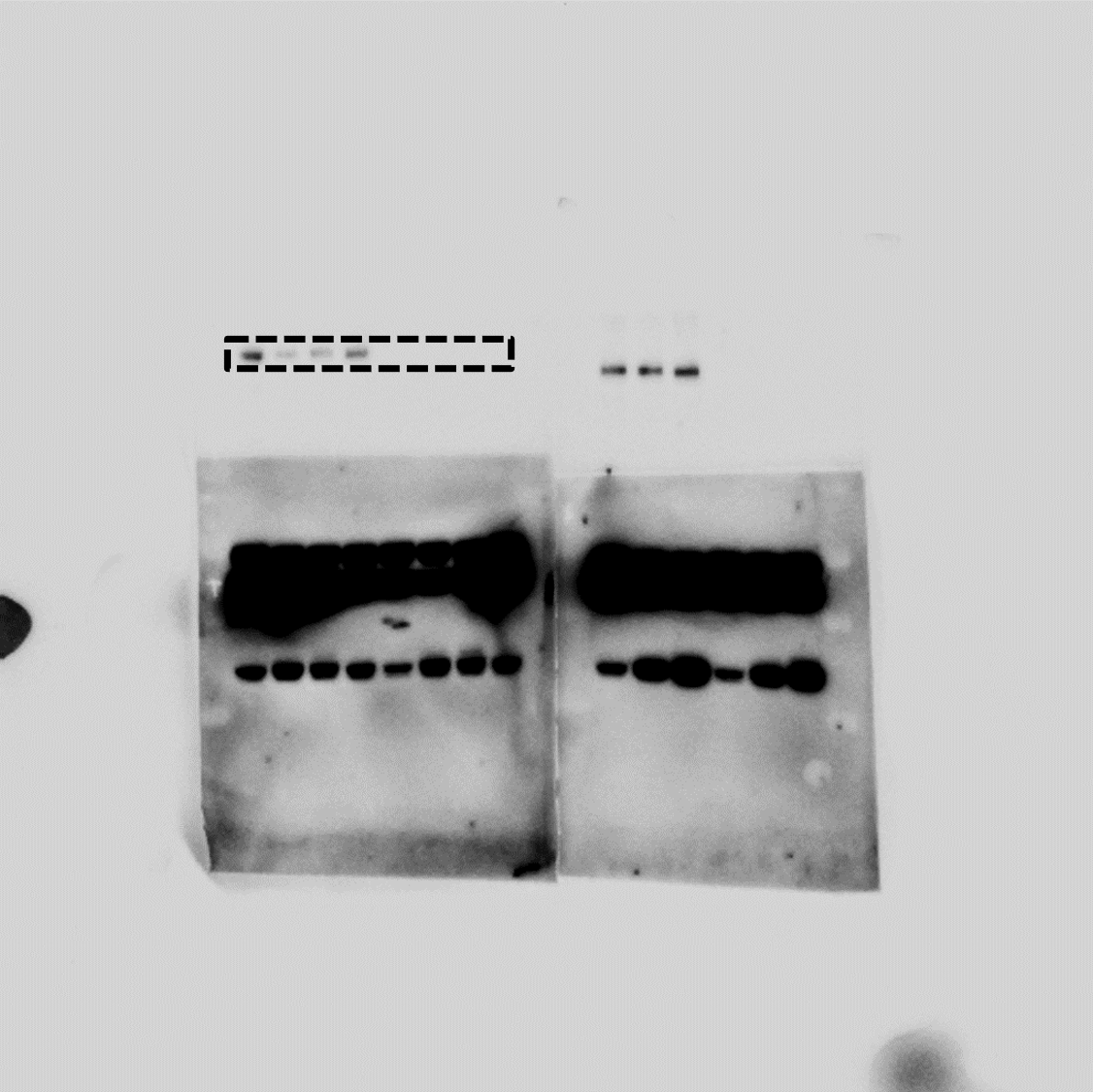

Supplement: Figure 6—source data 1. [file elife-98649-fig6-data1.zip › Figure 6-source data1/Figure 6A_JIP4_Topoisomerasei _annotated.tif]

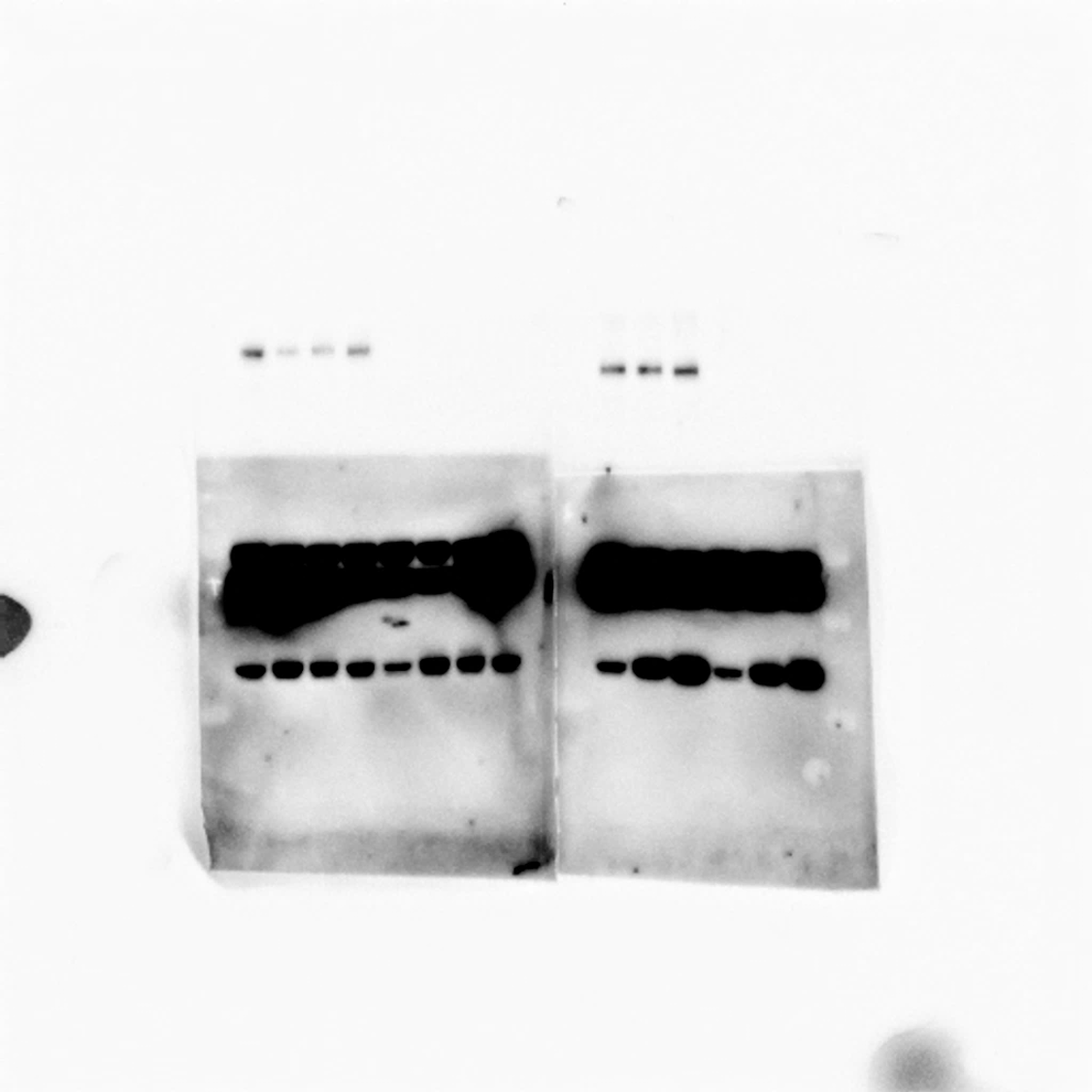

Supplement: Figure 6—source data 1. [file elife-98649-fig6-data1.zip › Figure 6-source data1/Figure 6A_JIP4_Topoisomerasei _raw.tif]

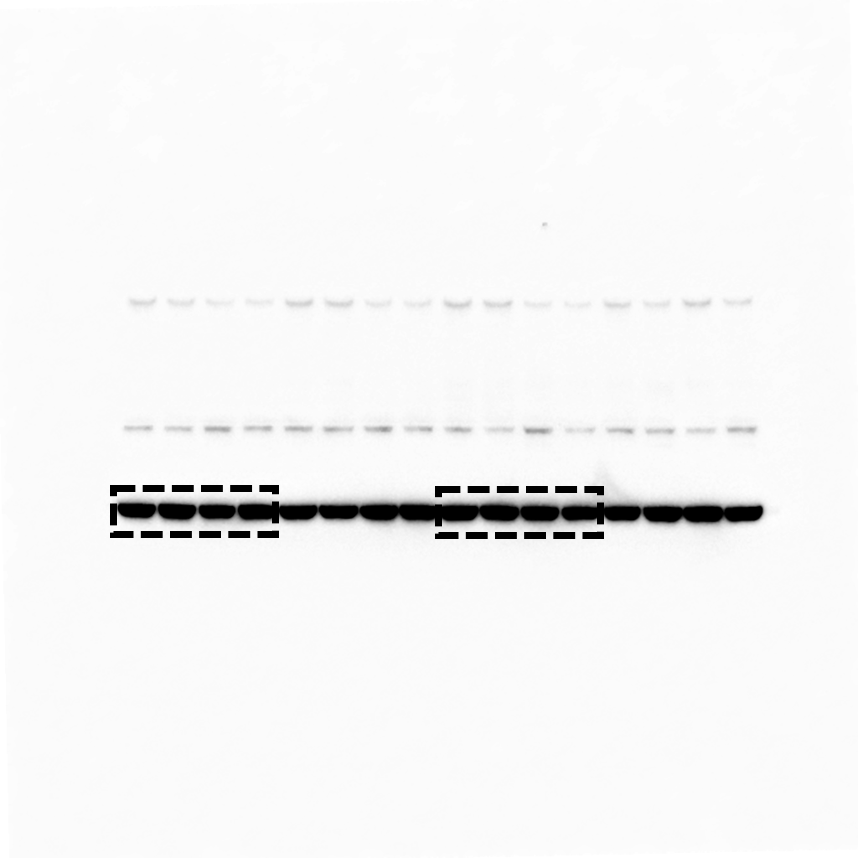

Supplement: Figure 6—source data 1. [file elife-98649-fig6-data1.zip › Figure 6-source data1/Figure 6D_actin_annotatated.tif]

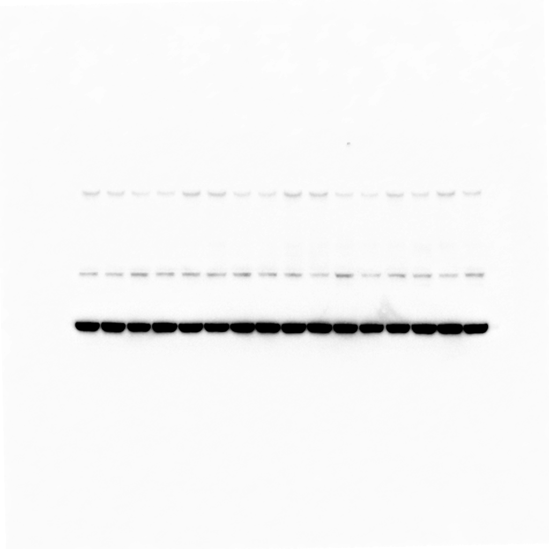

Supplement: Figure 6—source data 1. [file elife-98649-fig6-data1.zip › Figure 6-source data1/Figure 6D_actin_raw.tif]

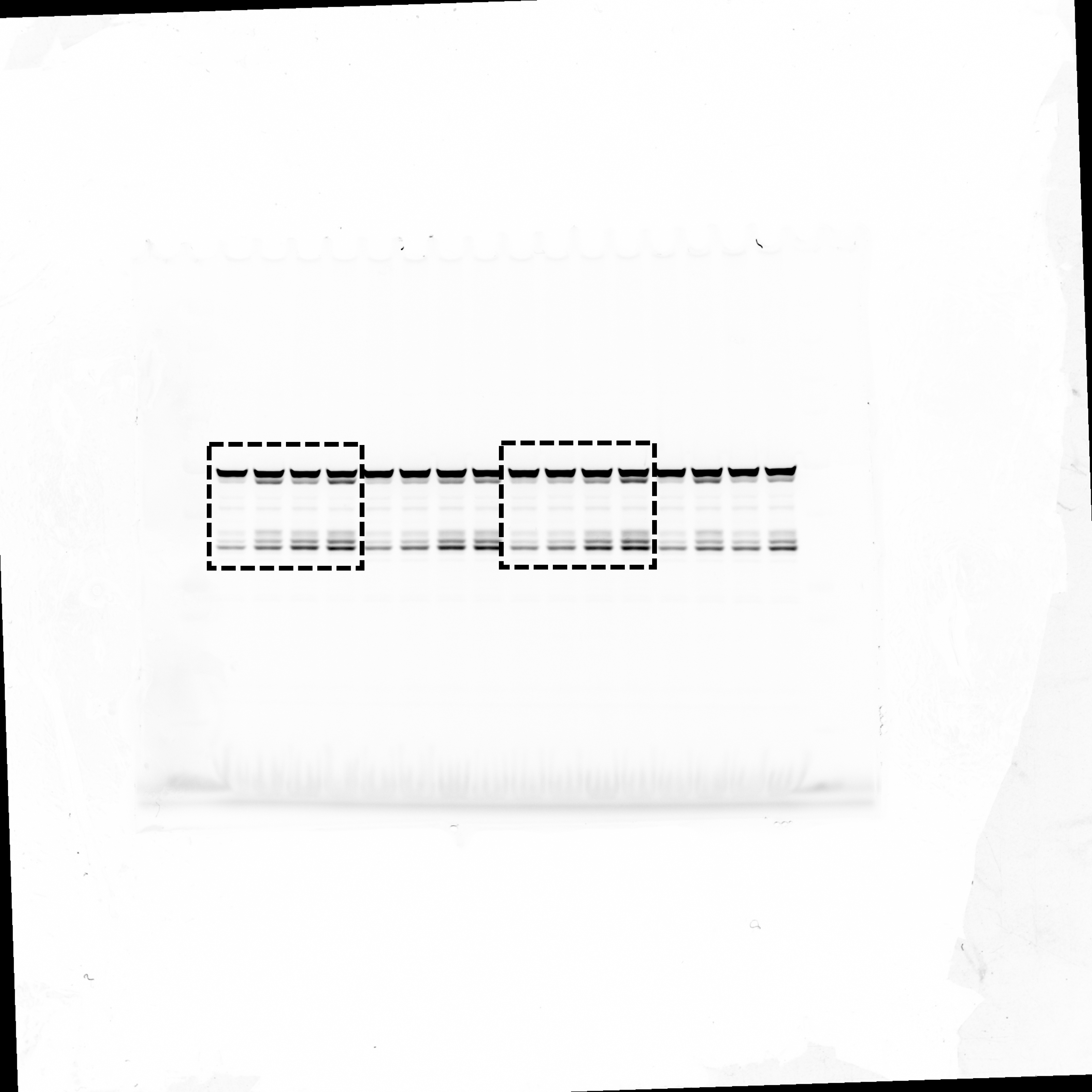

Supplement: Figure 6—source data 1. [file elife-98649-fig6-data1.zip › Figure 6-source data1/Figure 6D_gelfluorescence_annotated.tif]

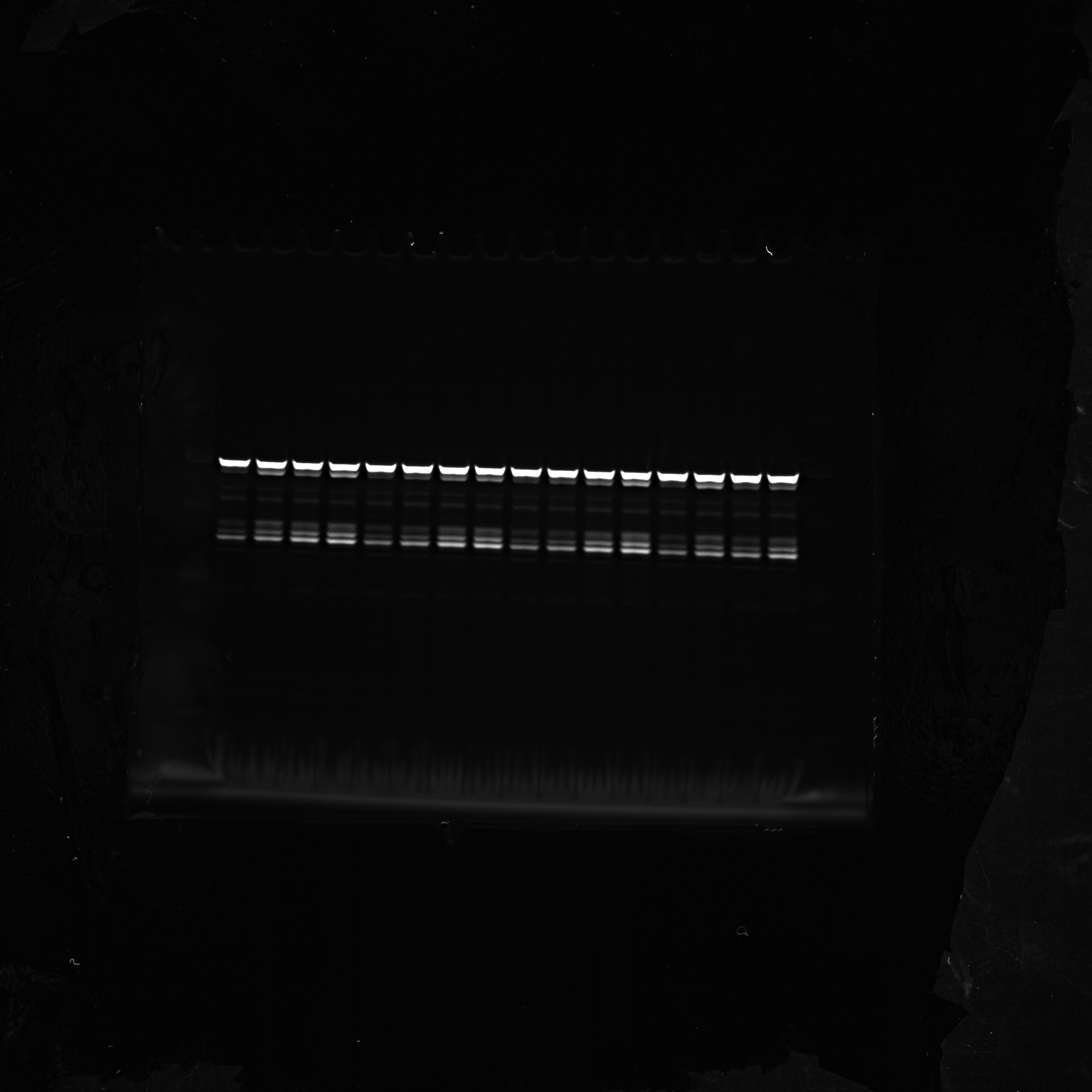

Supplement: Figure 6—source data 1. [file elife-98649-fig6-data1.zip › Figure 6-source data1/Figure 6D_gelfluorescence_raw.Tif]

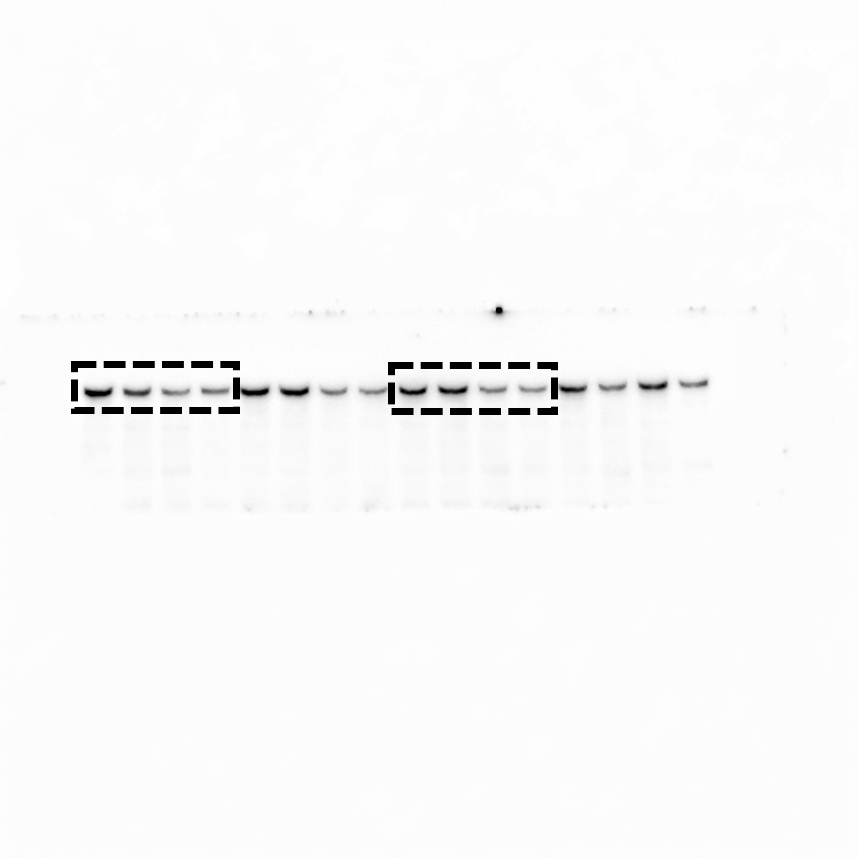

Supplement: Figure 6—source data 1. [file elife-98649-fig6-data1.zip › Figure 6-source data1/Figure 6D_p-mTOR_annotated.tif]

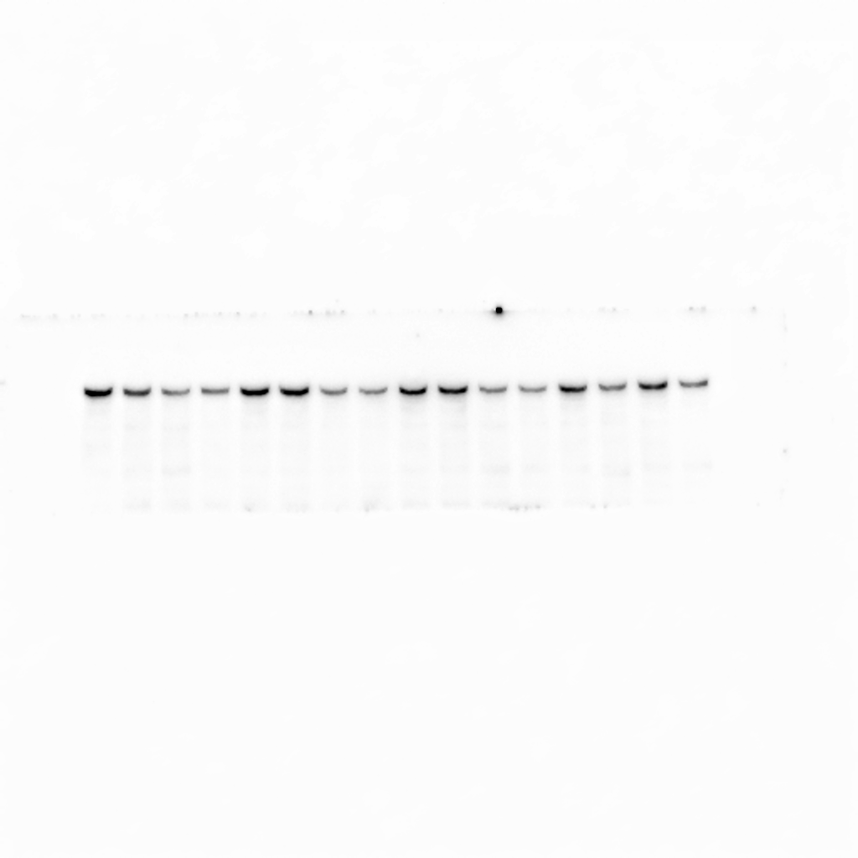

Supplement: Figure 6—source data 1. [file elife-98649-fig6-data1.zip › Figure 6-source data1/Figure 6D_p-mTOR_raw.tif]

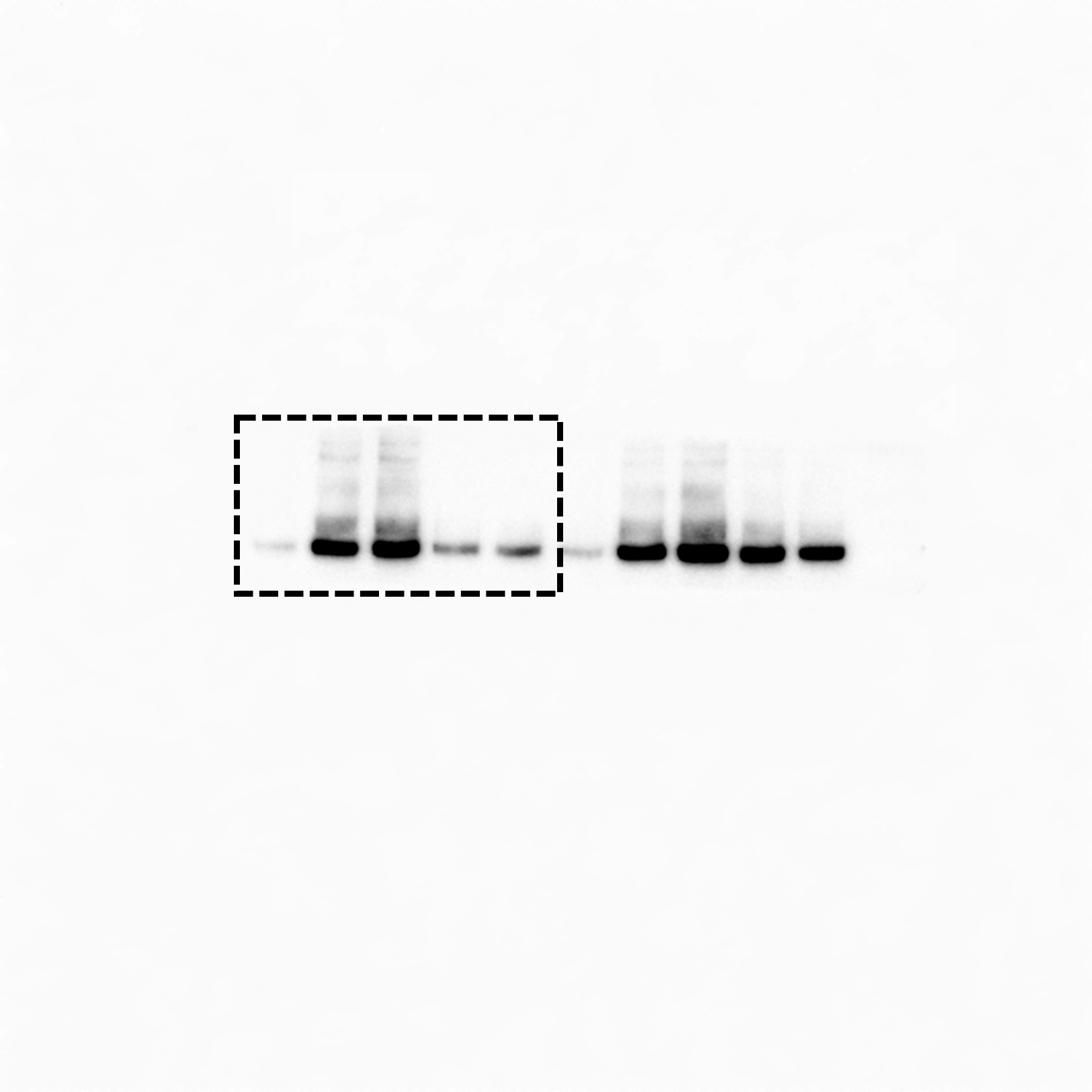

Supplement: Figure 7—source data 1. [file elife-98649-fig7-data1.zip › Figure 7-source data1/Figure 7A_p62_insoluble_annotated.tif]

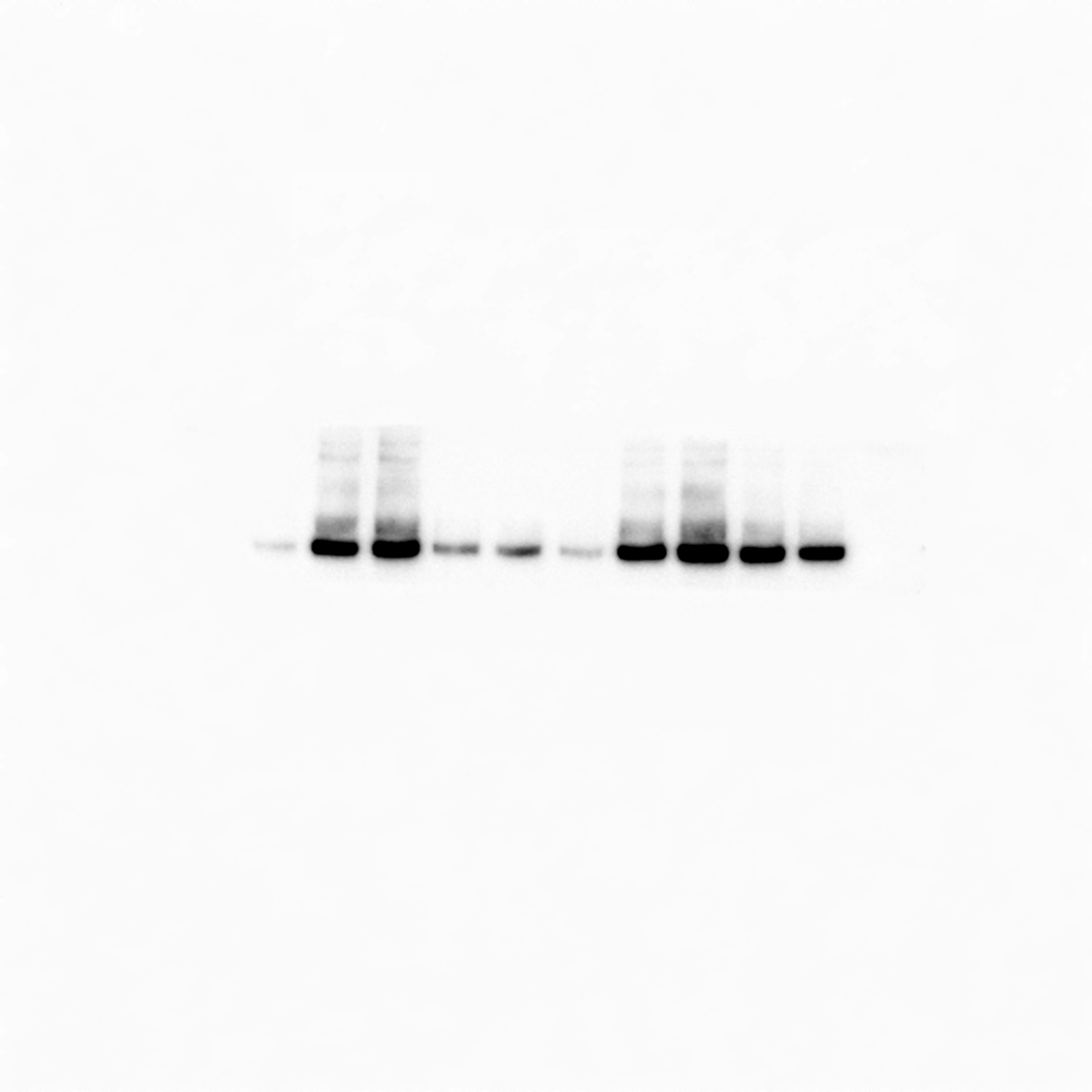

Supplement: Figure 7—source data 1. [file elife-98649-fig7-data1.zip › Figure 7-source data1/Figure 7A_p62_insoluble_raw.tif]

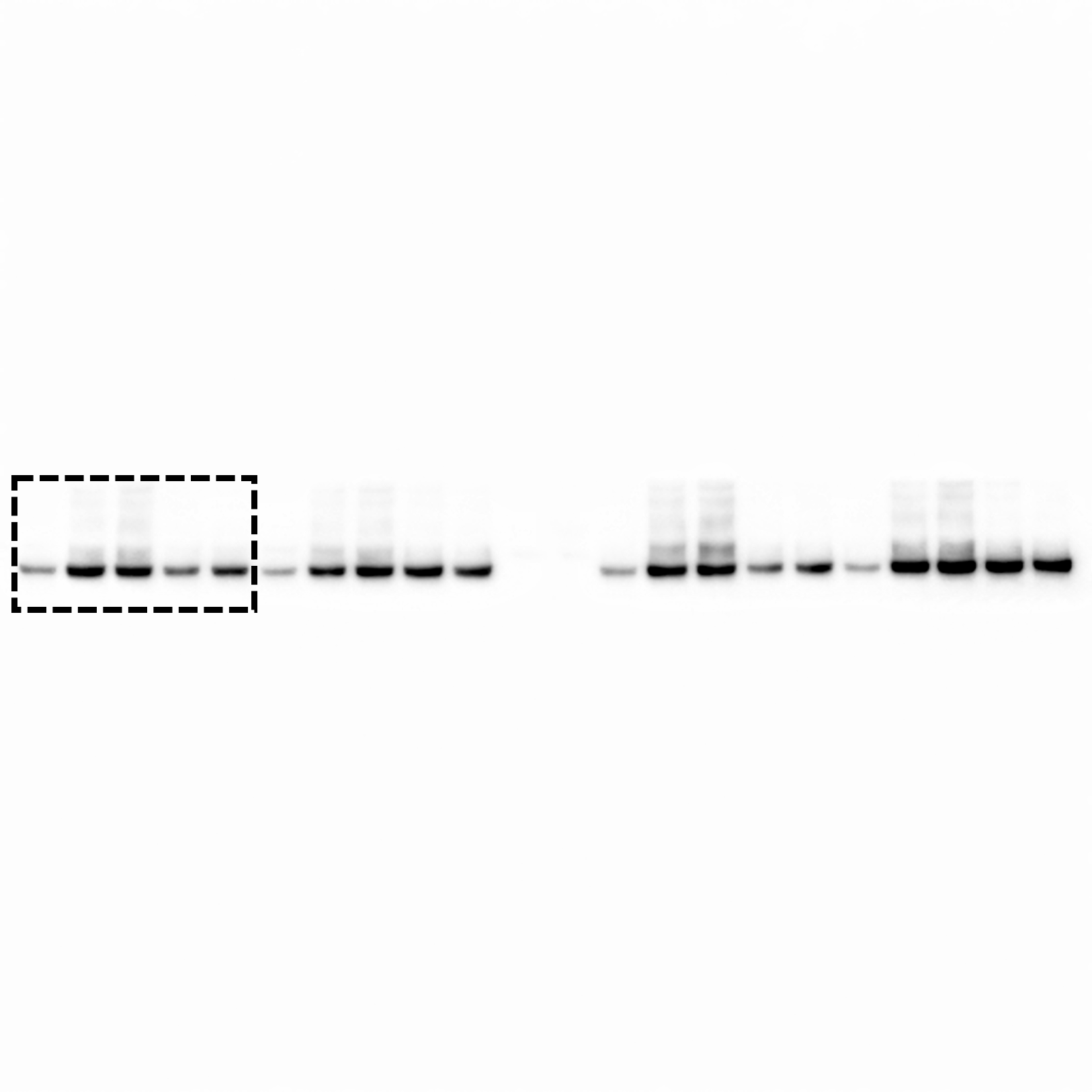

Supplement: Figure 7—source data 1. [file elife-98649-fig7-data1.zip › Figure 7-source data1/Figure 7A_p62_soluble_annotated.tif]

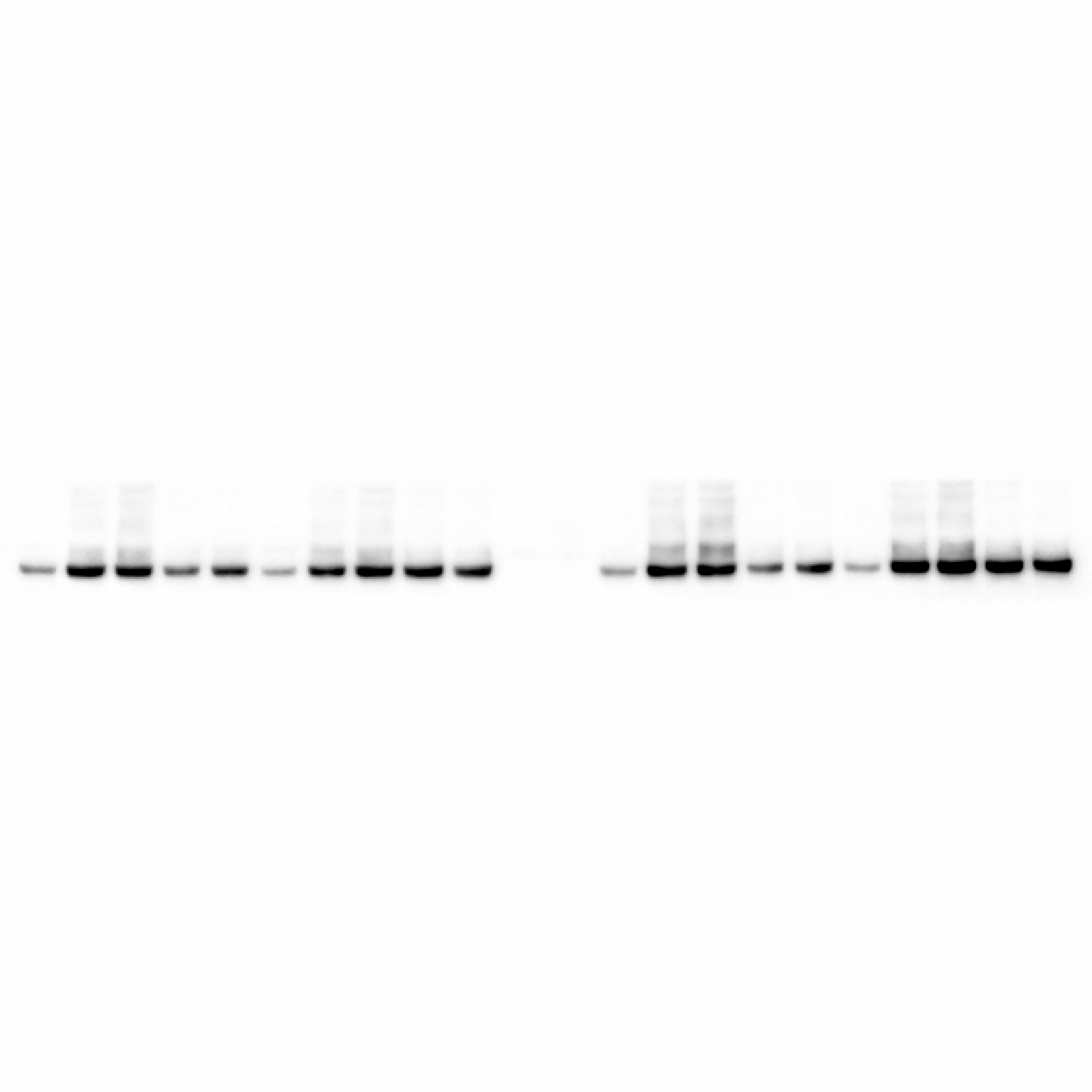

Supplement: Figure 7—source data 1. [file elife-98649-fig7-data1.zip › Figure 7-source data1/Figure 7A_p62_soluble_raw.tif]

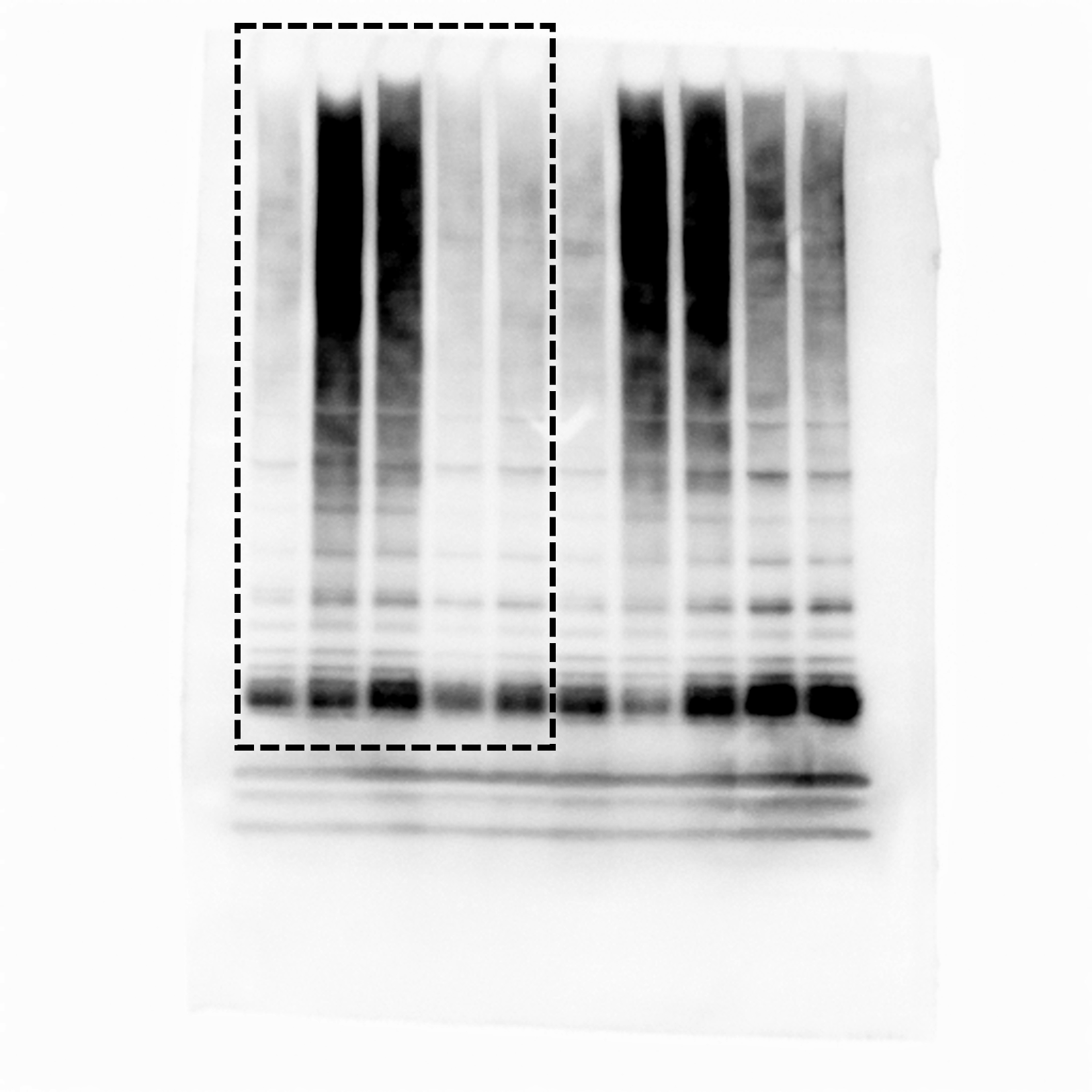

Supplement: Figure 7—source data 1. [file elife-98649-fig7-data1.zip › Figure 7-source data1/Figure 7A_ub_insoluble_annotated.tif]

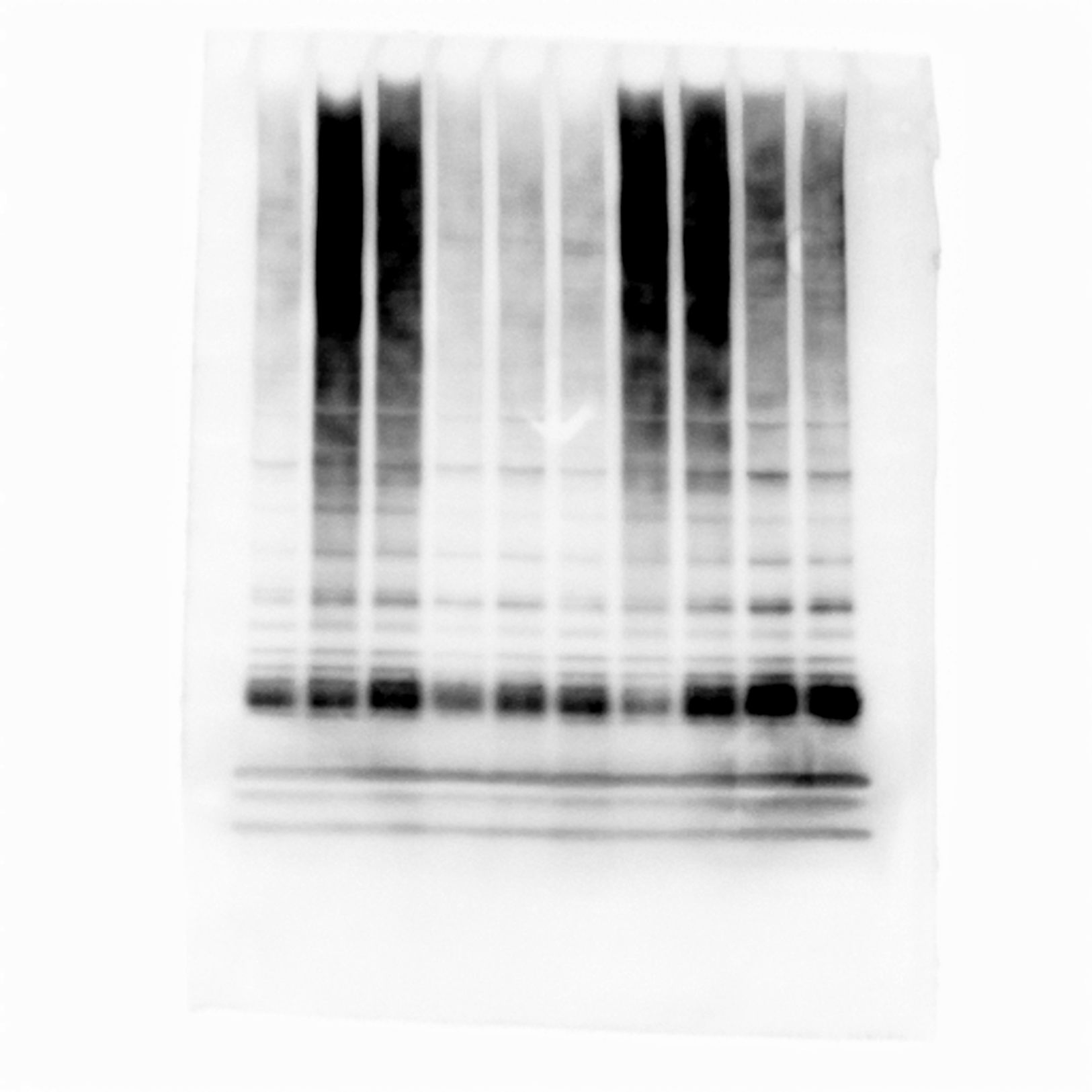

Supplement: Figure 7—source data 1. [file elife-98649-fig7-data1.zip › Figure 7-source data1/Figure 7A_ub_insoluble_raw.tif]

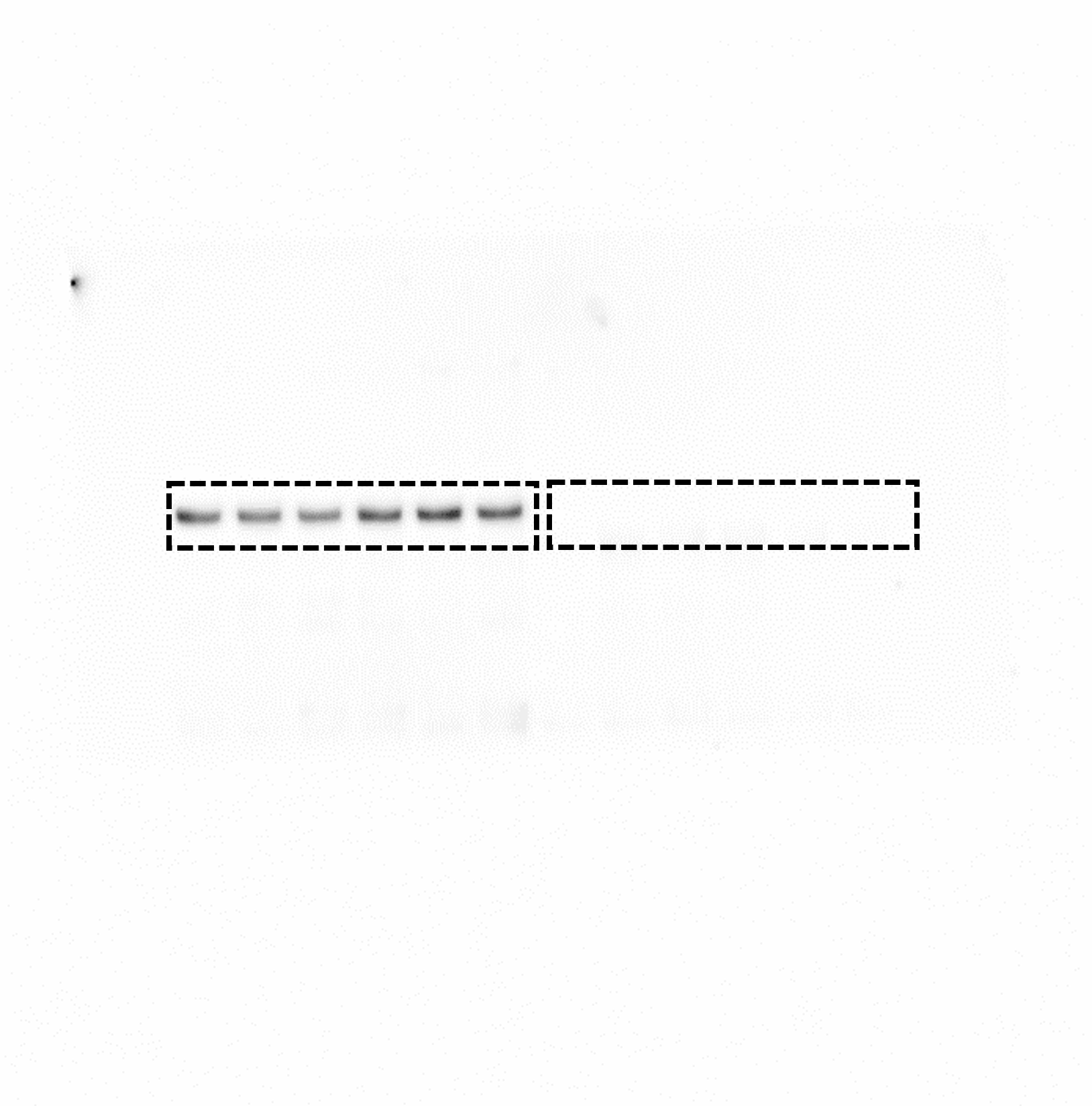

Supplement: Figure 7—source data 1. [file elife-98649-fig7-data1.zip › Figure 7-source data1/Figure 7B_FIP200_soluble_annotated.tif]

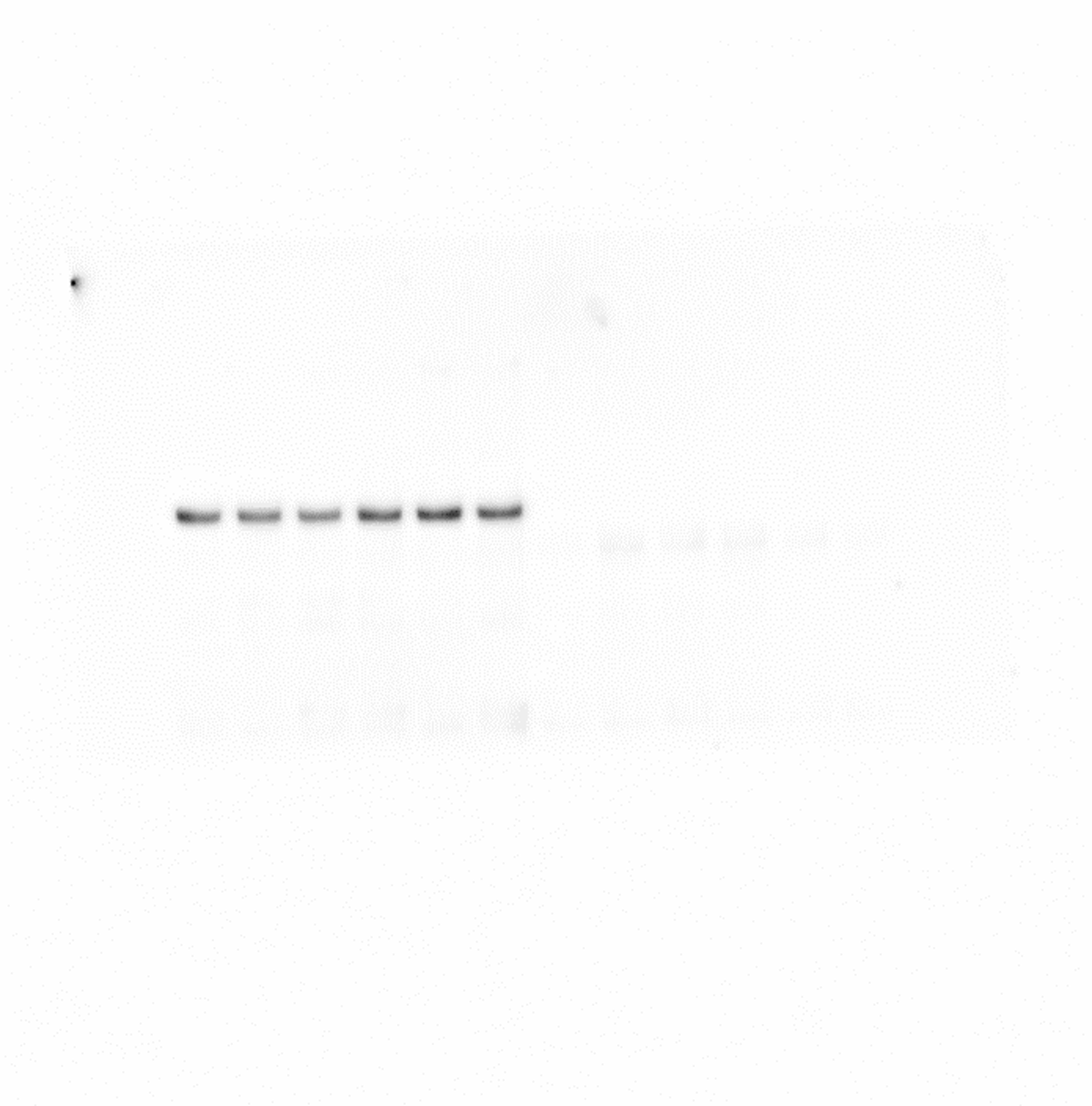

Supplement: Figure 7—source data 1. [file elife-98649-fig7-data1.zip › Figure 7-source data1/Figure 7B_FIP200_soluble_raw.tif]

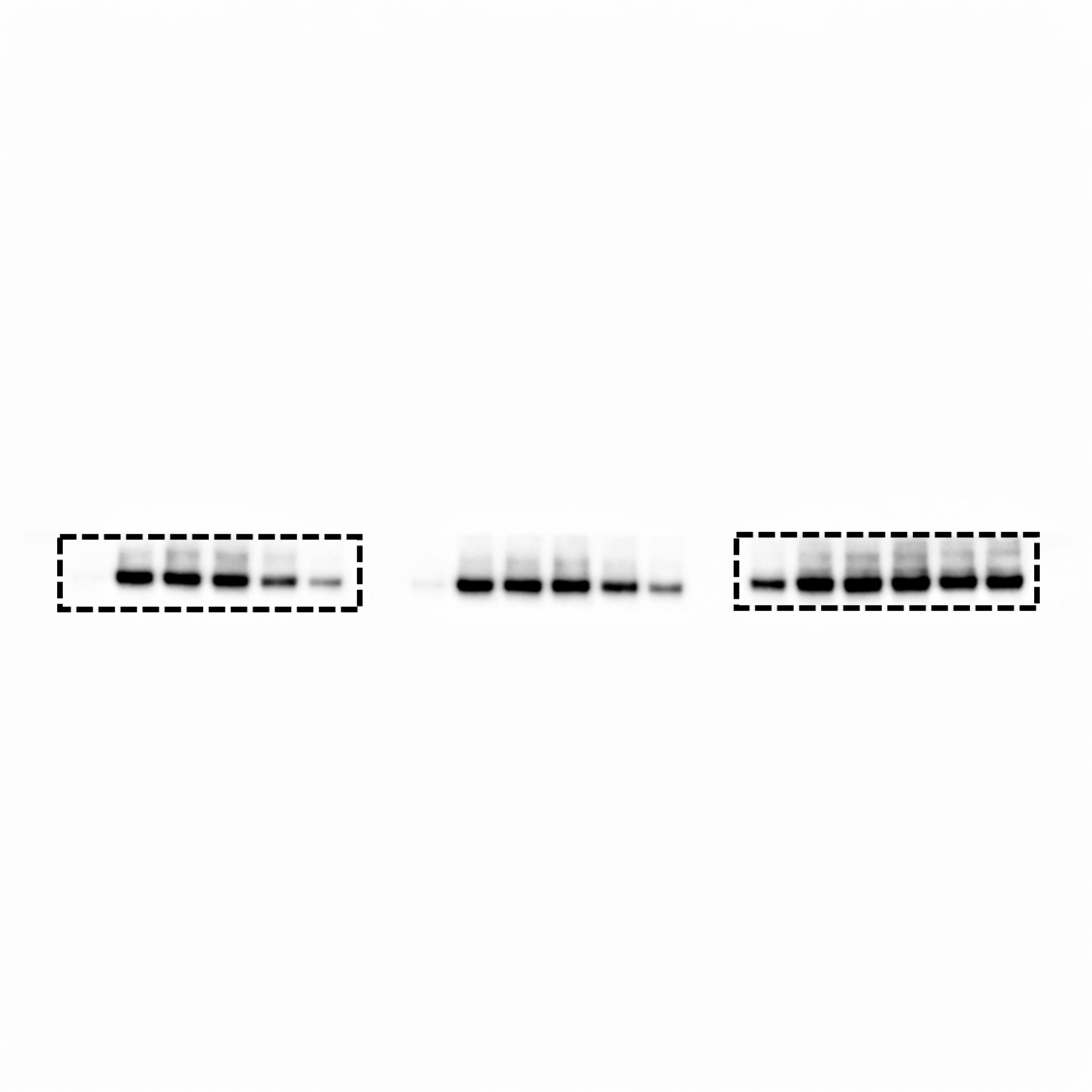

Supplement: Figure 7—source data 1. [file elife-98649-fig7-data1.zip › Figure 7-source data1/Figure 7B_p62_insoluble_annotated.tif]

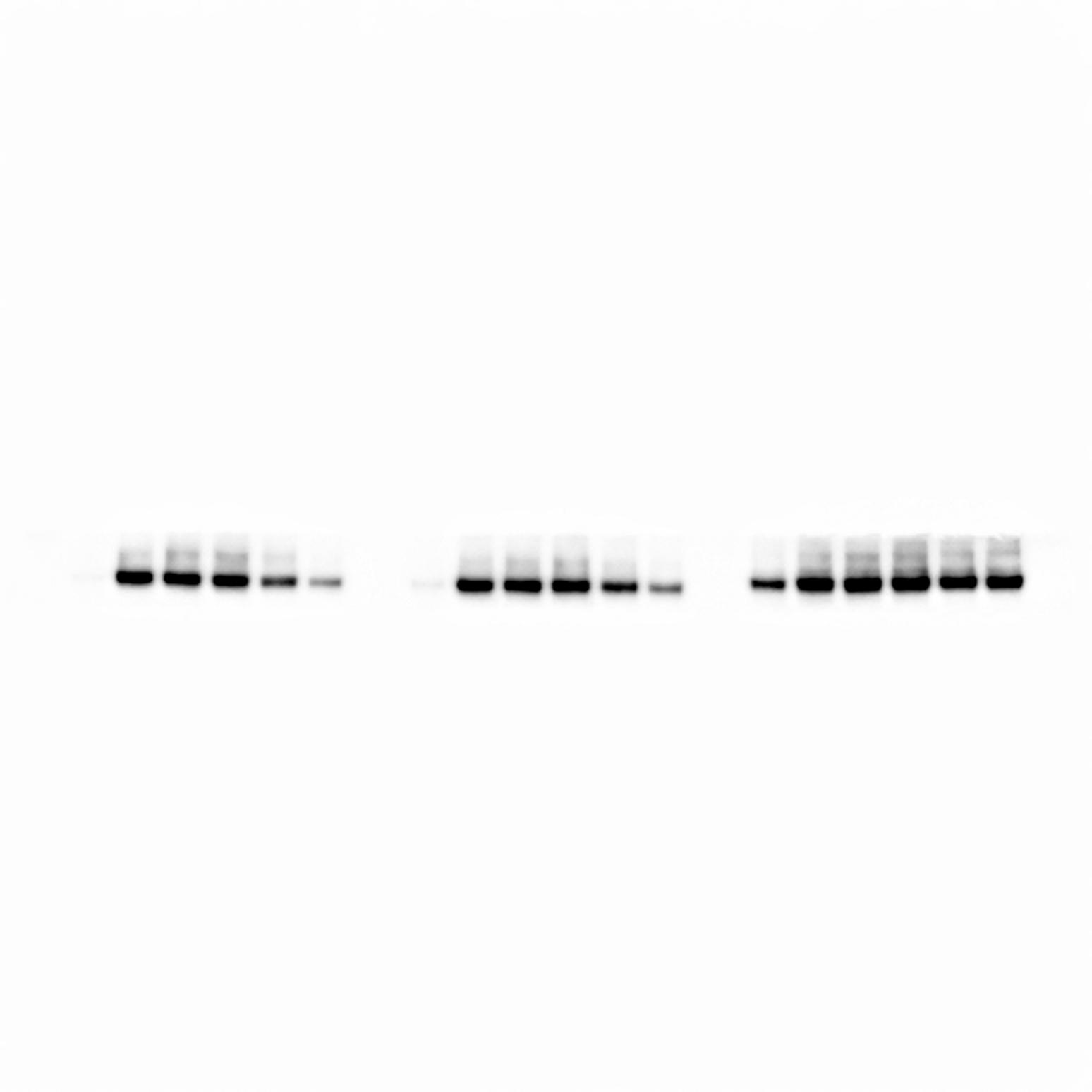

Supplement: Figure 7—source data 1. [file elife-98649-fig7-data1.zip › Figure 7-source data1/Figure 7B_p62_insoluble_raw.tif]

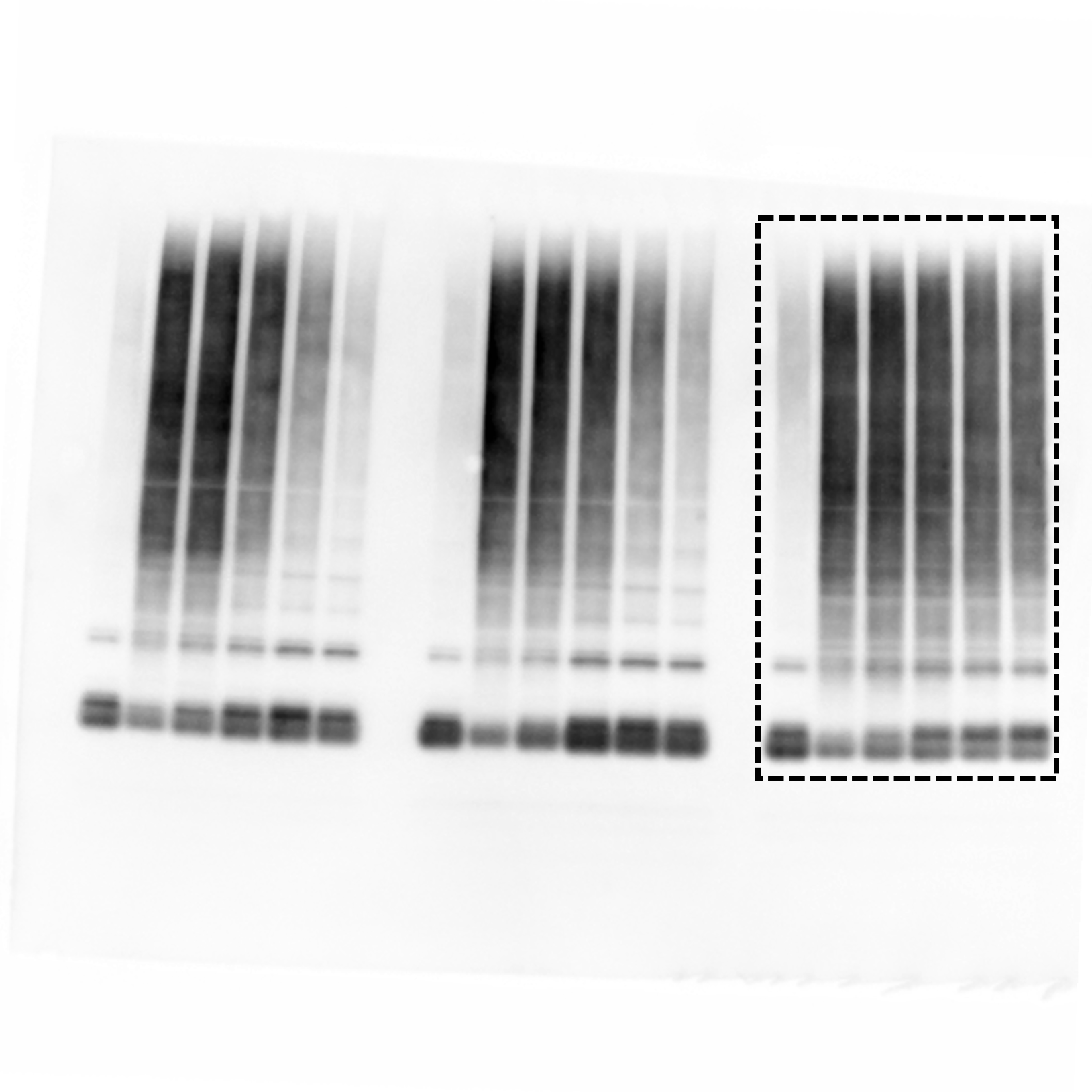

Supplement: Figure 7—source data 1. [file elife-98649-fig7-data1.zip › Figure 7-source data1/Figure 7B_ub_insoluble_annotated.tif]

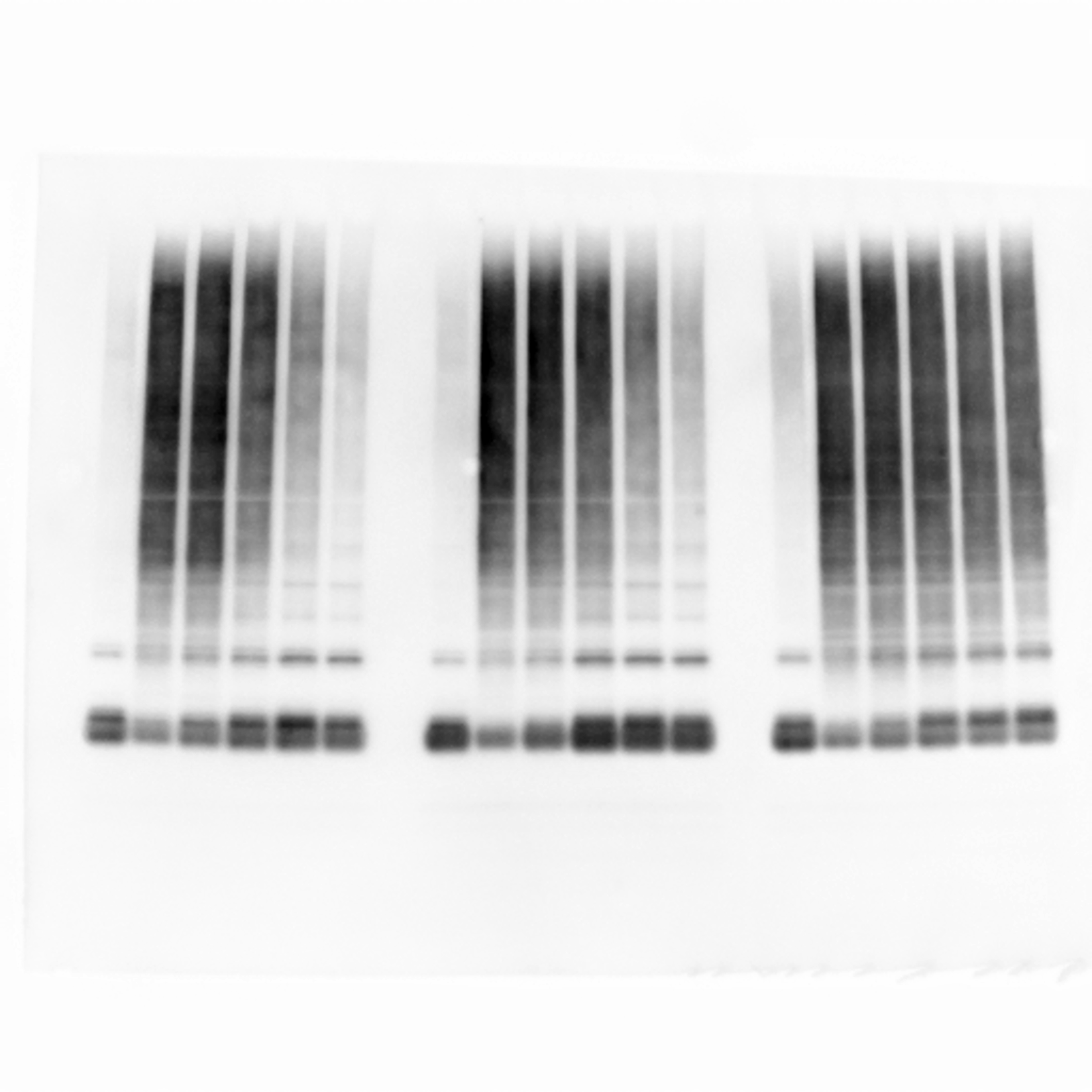

Supplement: Figure 7—source data 1. [file elife-98649-fig7-data1.zip › Figure 7-source data1/Figure 7B_ub_insoluble_raw.tif]

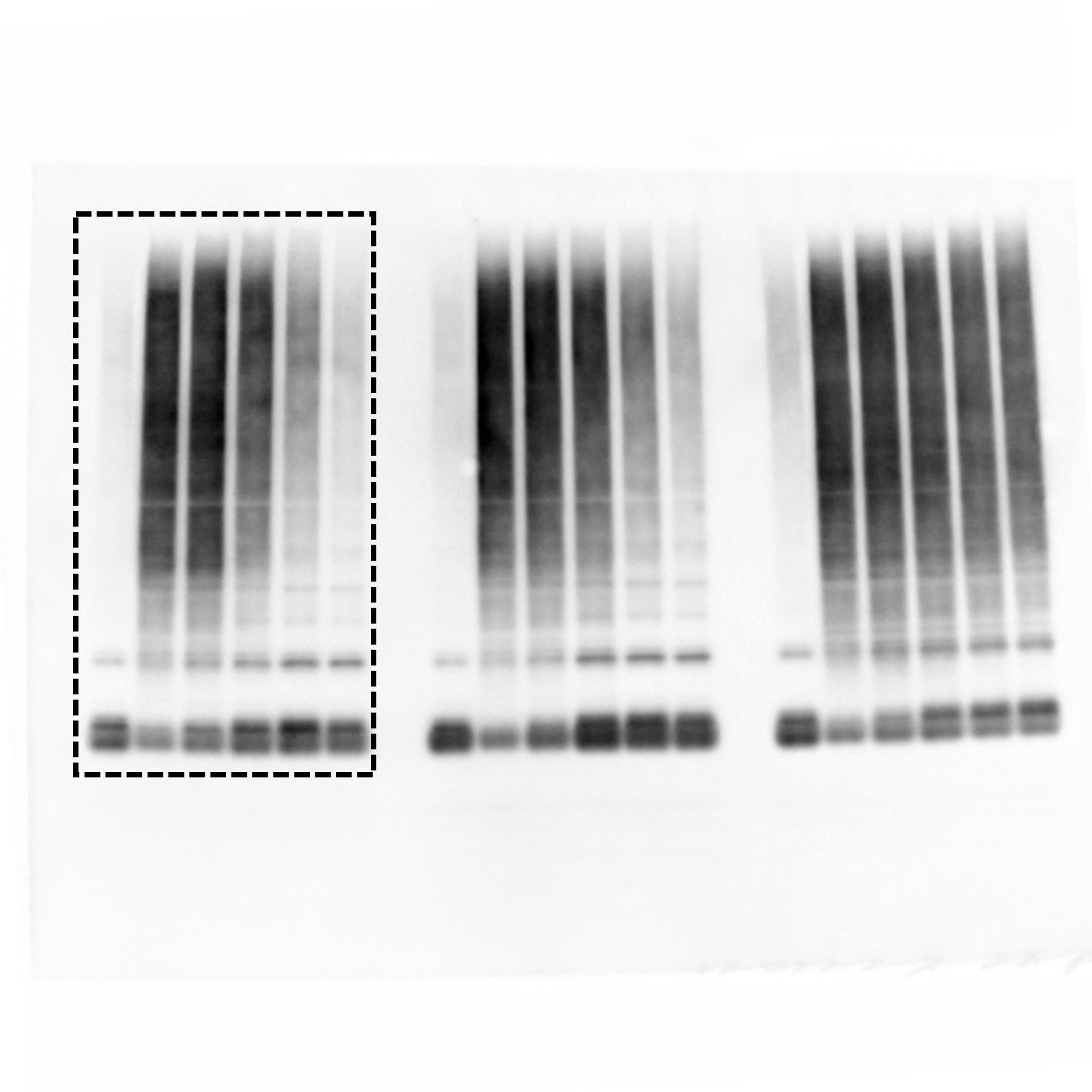

Supplement: Figure 7—source data 1. [file elife-98649-fig7-data1.zip › Figure 7-source data1/Figure 7B_ub_insoluble_WT_annotated.tif]

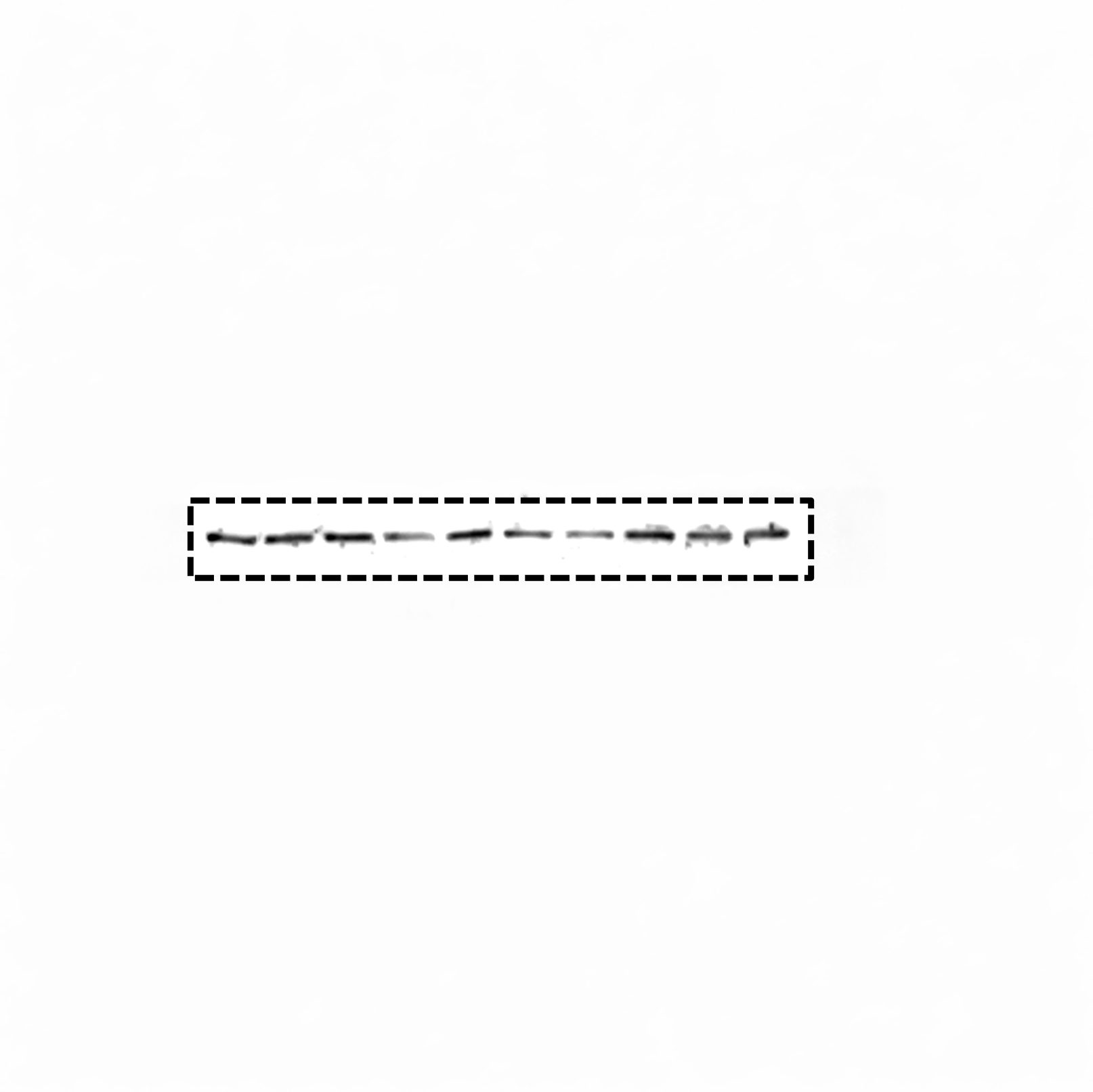

Supplement: Figure 7—source data 1. [file elife-98649-fig7-data1.zip › Figure 7-source data1/Figure 7C_actin_insoluble_annotated.tif]

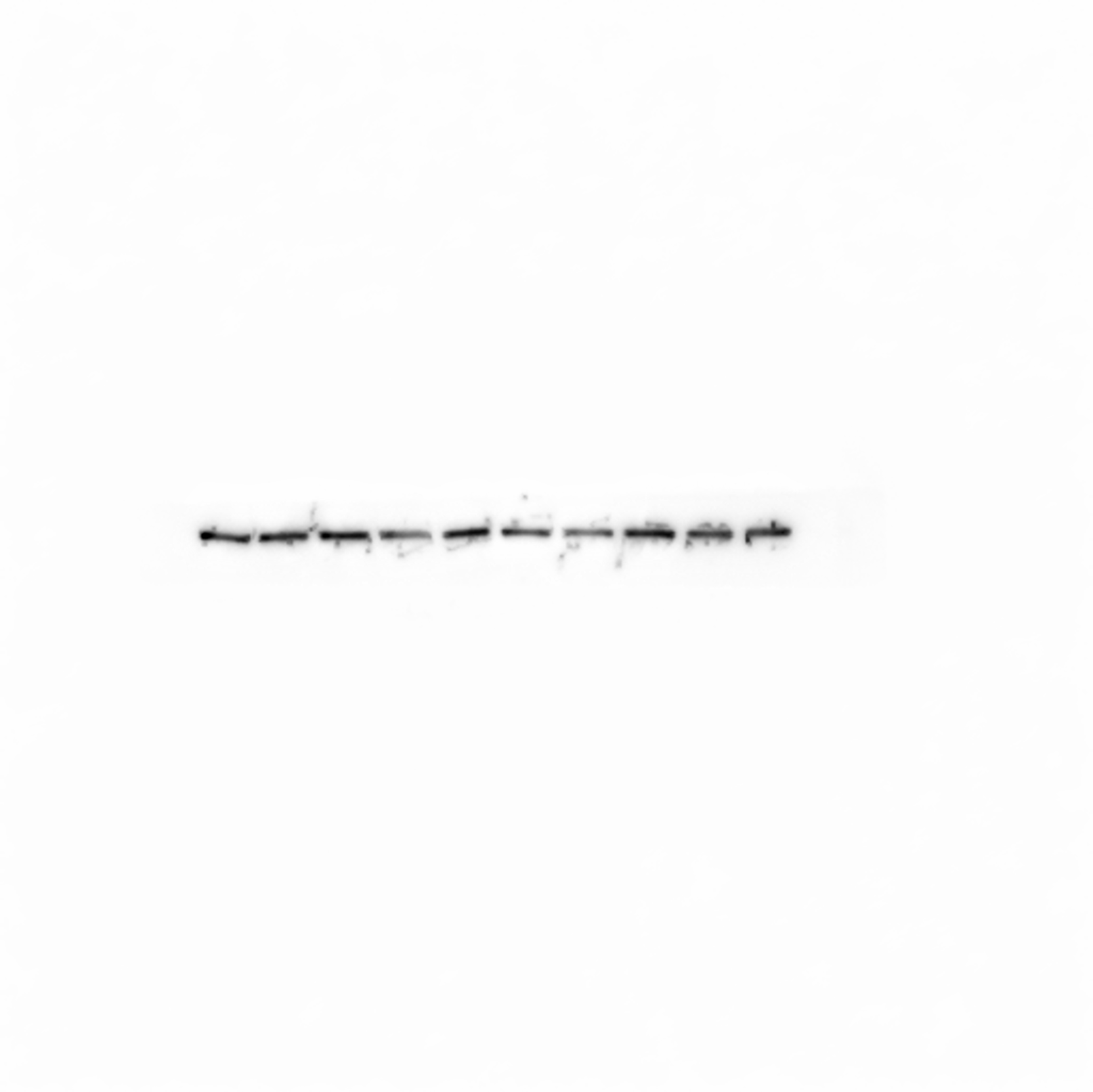

Supplement: Figure 7—source data 1. [file elife-98649-fig7-data1.zip › Figure 7-source data1/Figure 7C_actin_insoluble_raw.tif]

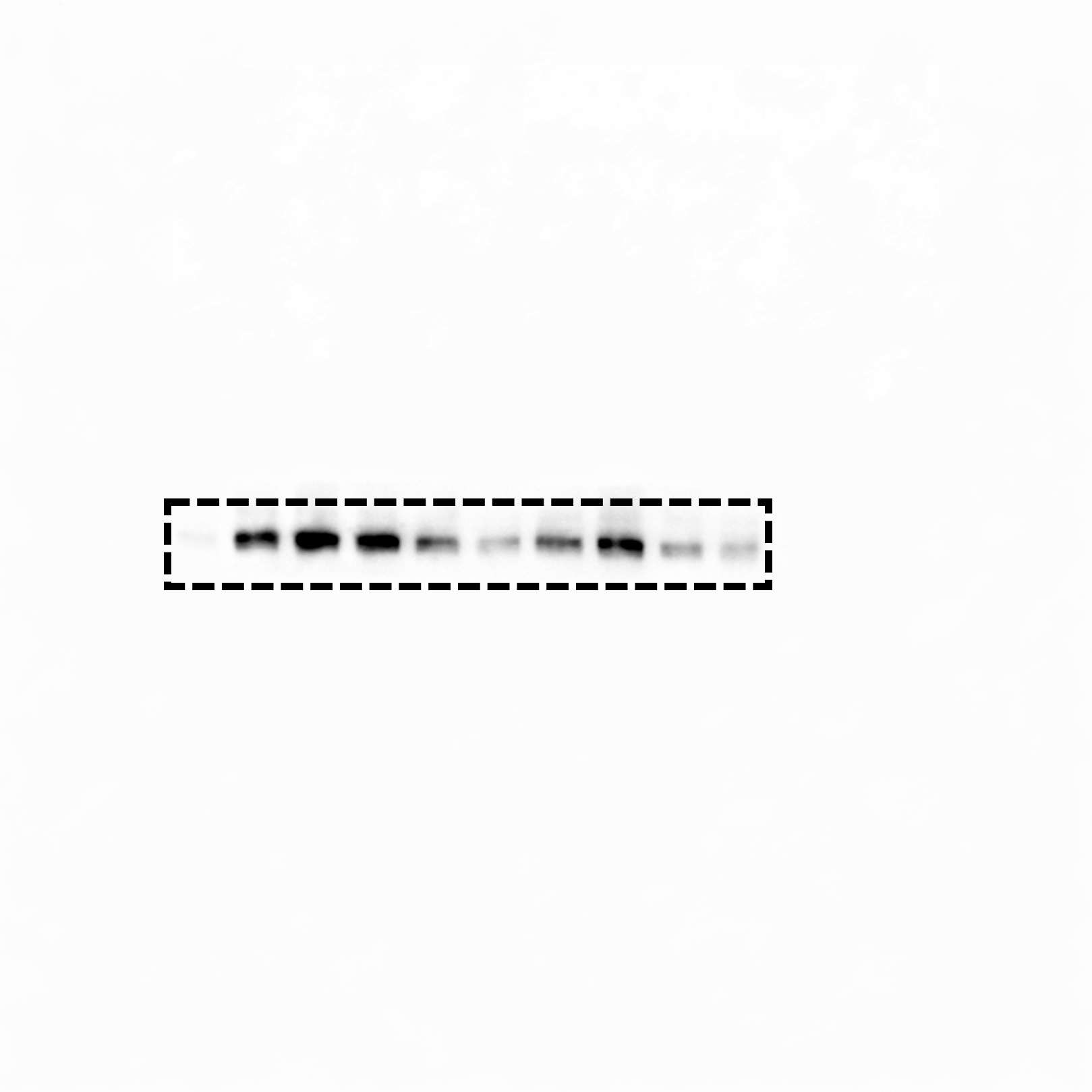

Supplement: Figure 7—source data 1. [file elife-98649-fig7-data1.zip › Figure 7-source data1/Figure 7C_p62_insoluble_annotated.tif]

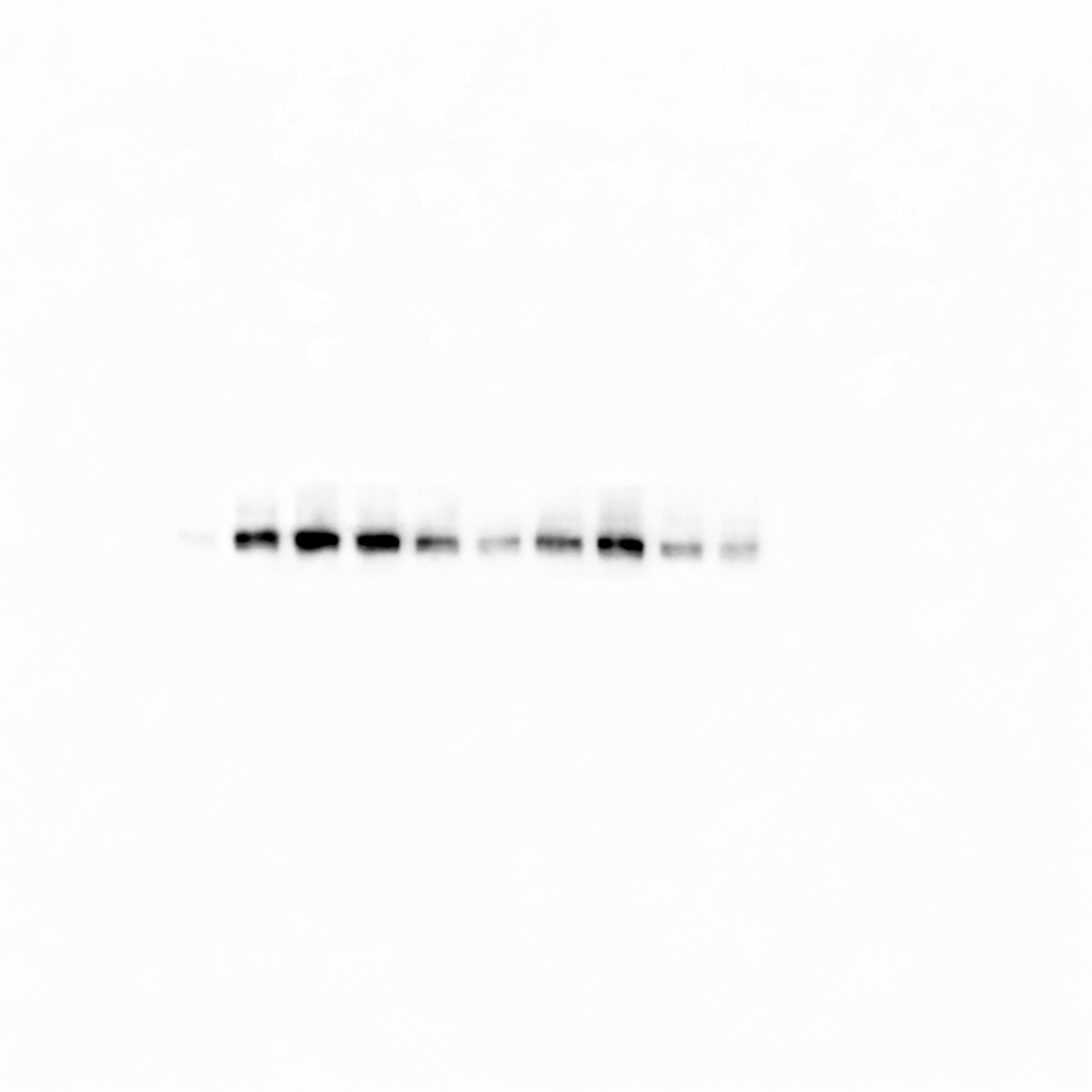

Supplement: Figure 7—source data 1. [file elife-98649-fig7-data1.zip › Figure 7-source data1/Figure 7C_p62_insoluble_raw.tif]

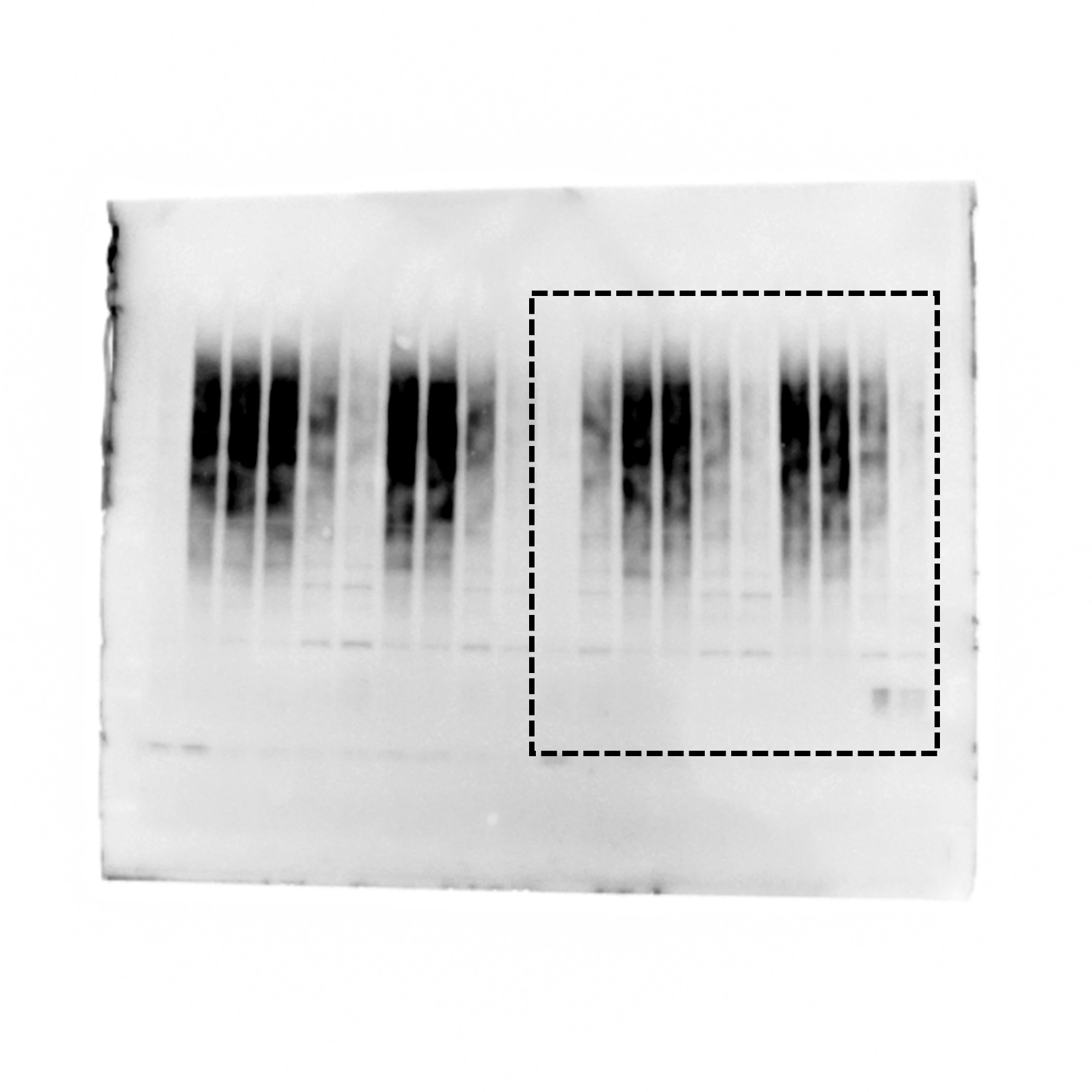

Supplement: Figure 7—source data 1. [file elife-98649-fig7-data1.zip › Figure 7-source data1/Figure 7C_ub_insoluble_annotated.tif]

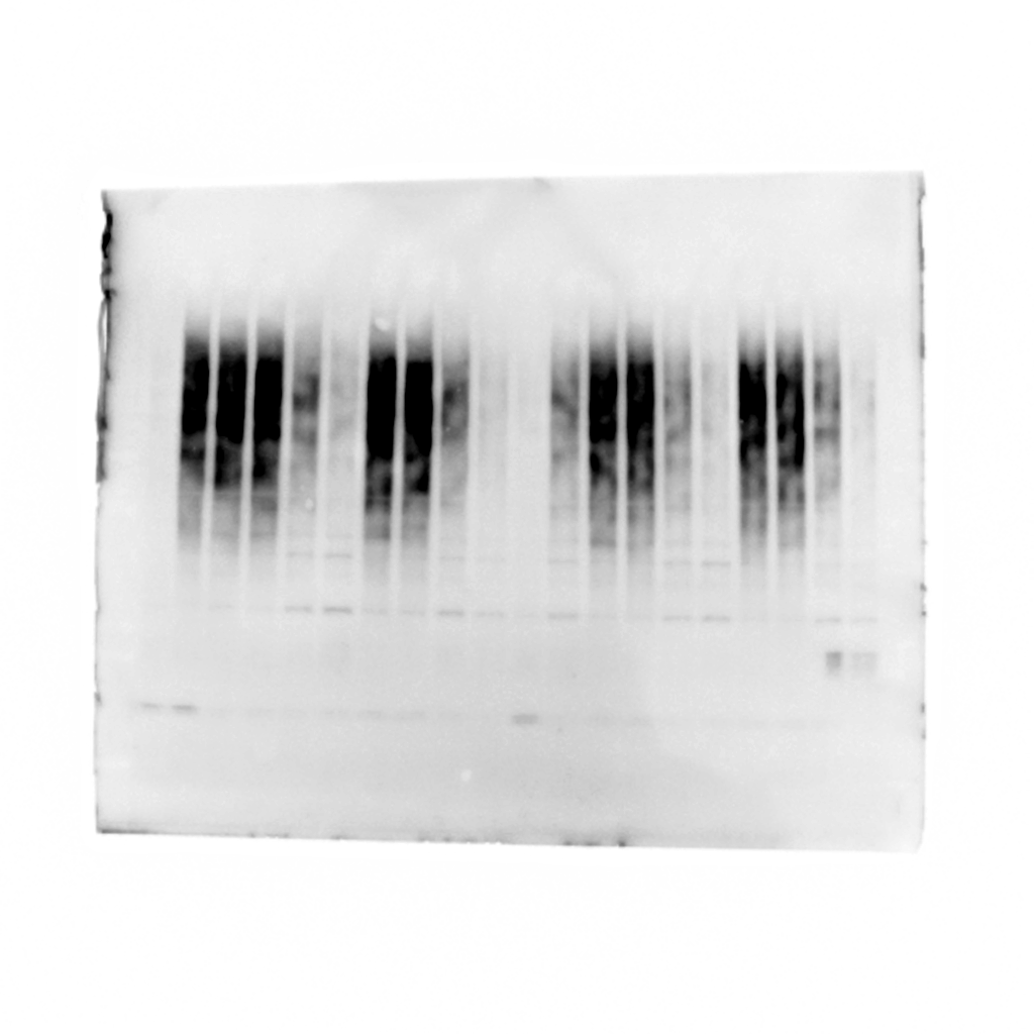

Supplement: Figure 7—source data 1. [file elife-98649-fig7-data1.zip › Figure 7-source data1/Figure 7C_ub_insoluble_raw.tif]

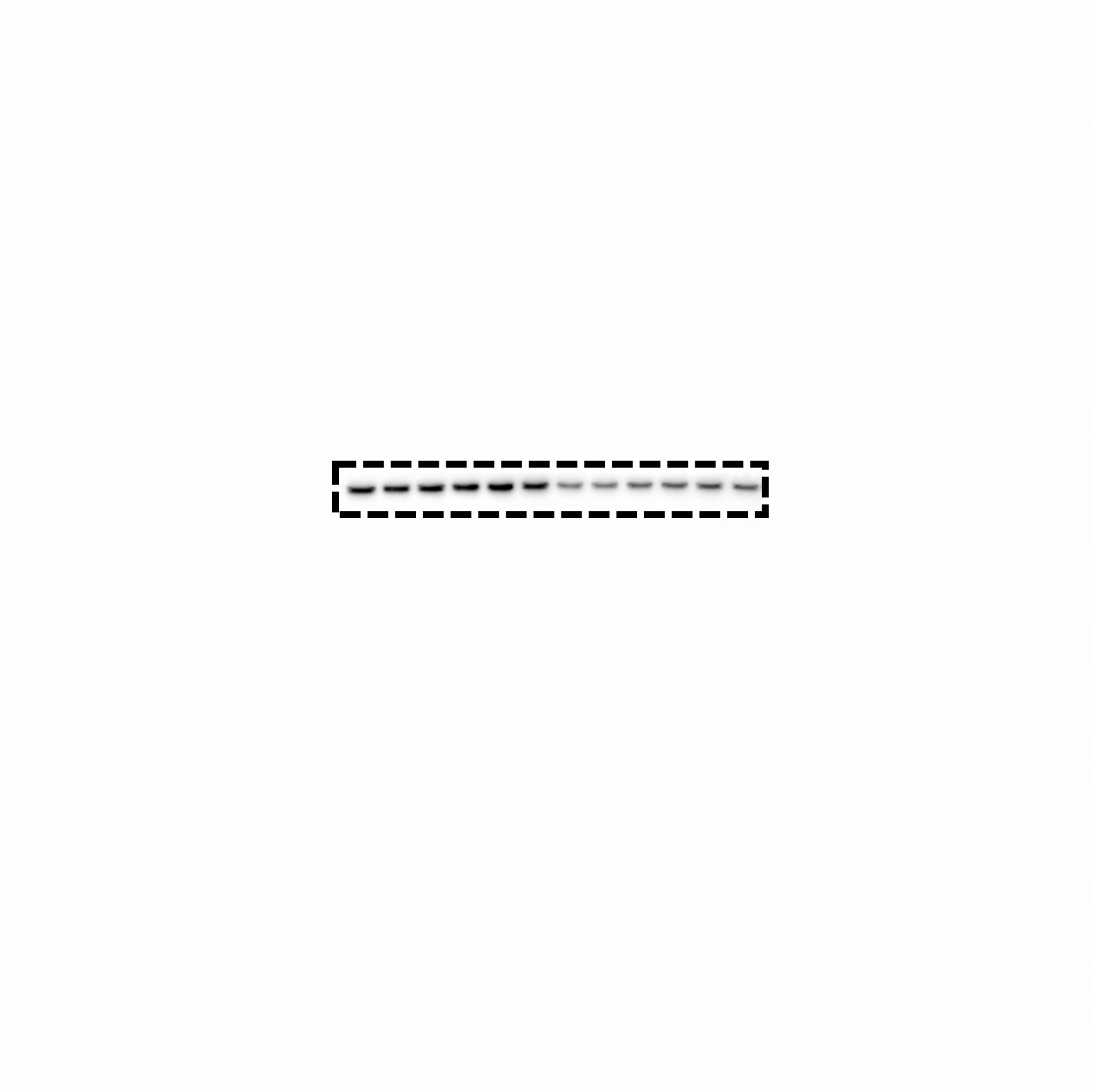

Supplement: Figure 7—source data 1. [file elife-98649-fig7-data1.zip › Figure 7-source data1/Figure 7D_actin_insoluble_annotated.tif]

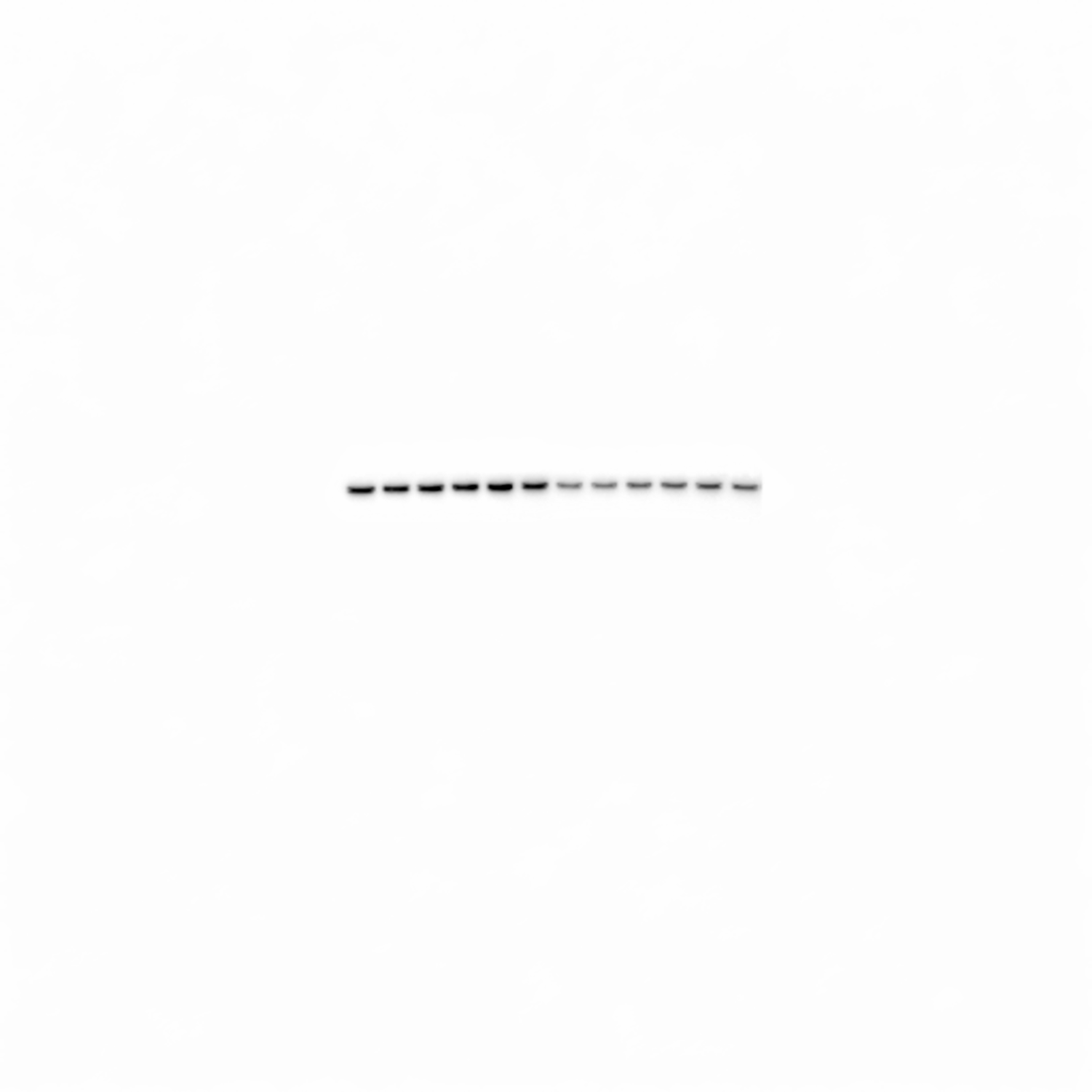

Supplement: Figure 7—source data 1. [file elife-98649-fig7-data1.zip › Figure 7-source data1/Figure 7D_actin_insoluble_raw.tif]

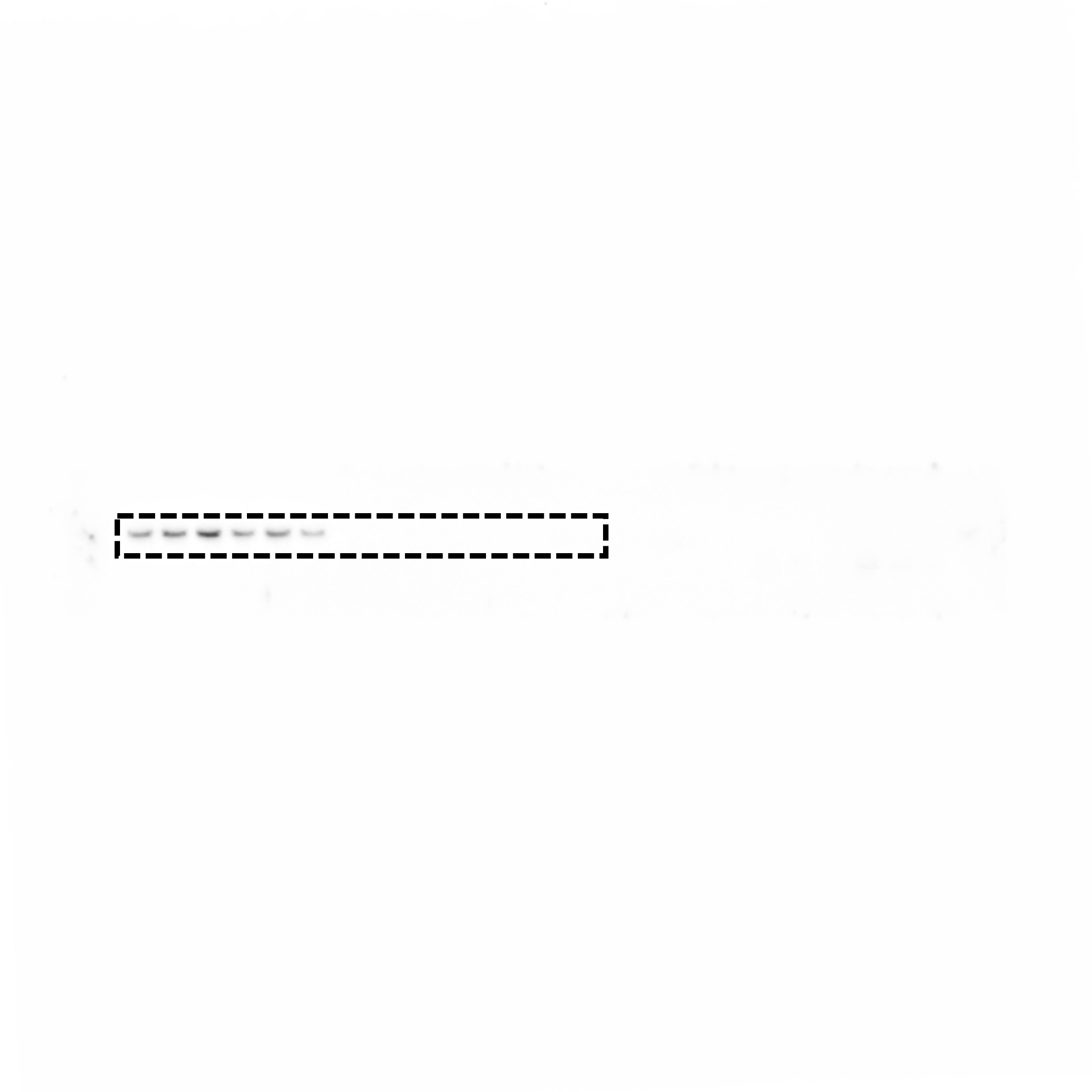

Supplement: Figure 7—source data 1. [file elife-98649-fig7-data1.zip › Figure 7-source data1/Figure 7D_FIP200_soluble_annotated.tif]

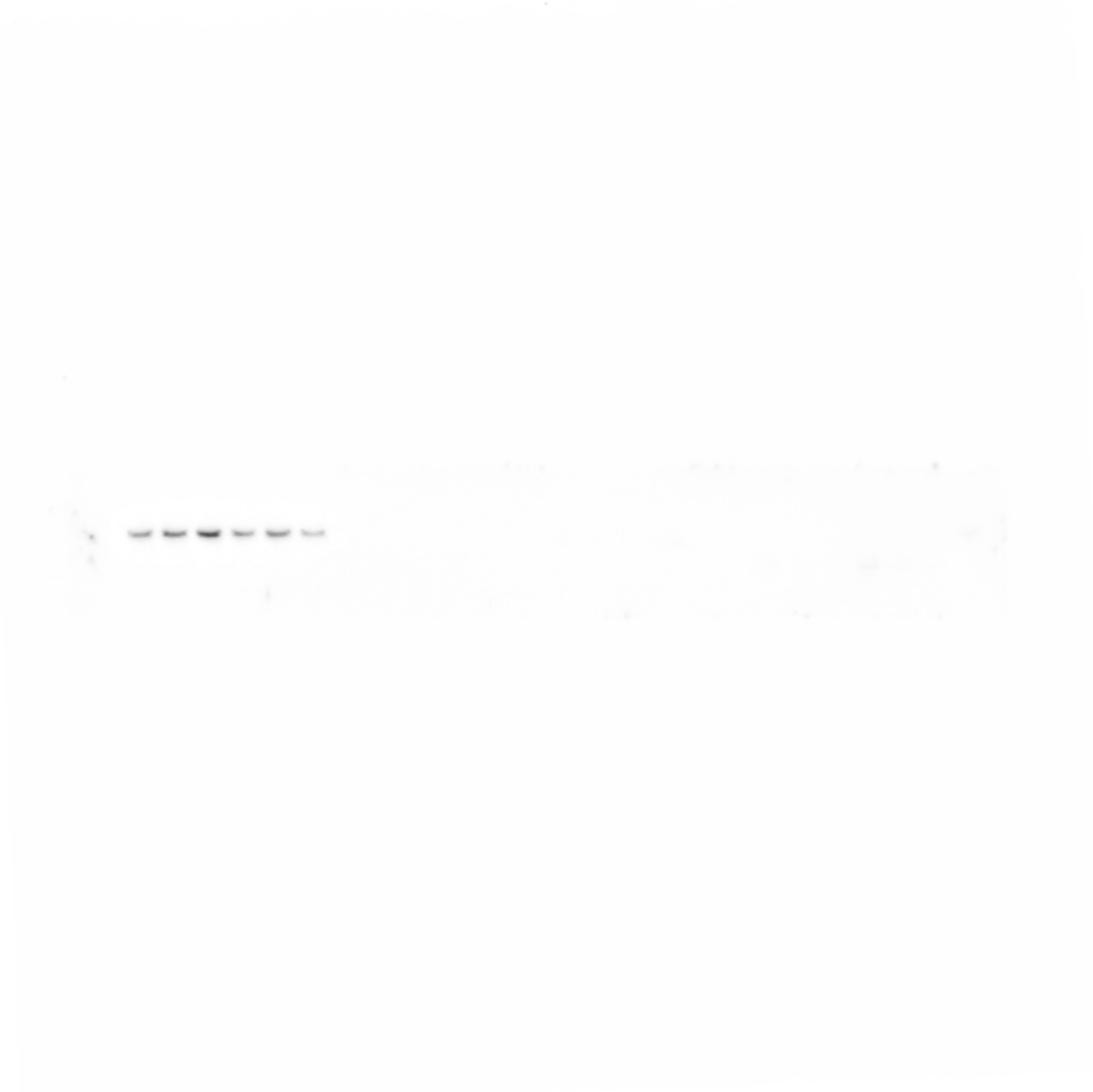

Supplement: Figure 7—source data 1. [file elife-98649-fig7-data1.zip › Figure 7-source data1/Figure 7D_FIP200_soluble_raw.tif]

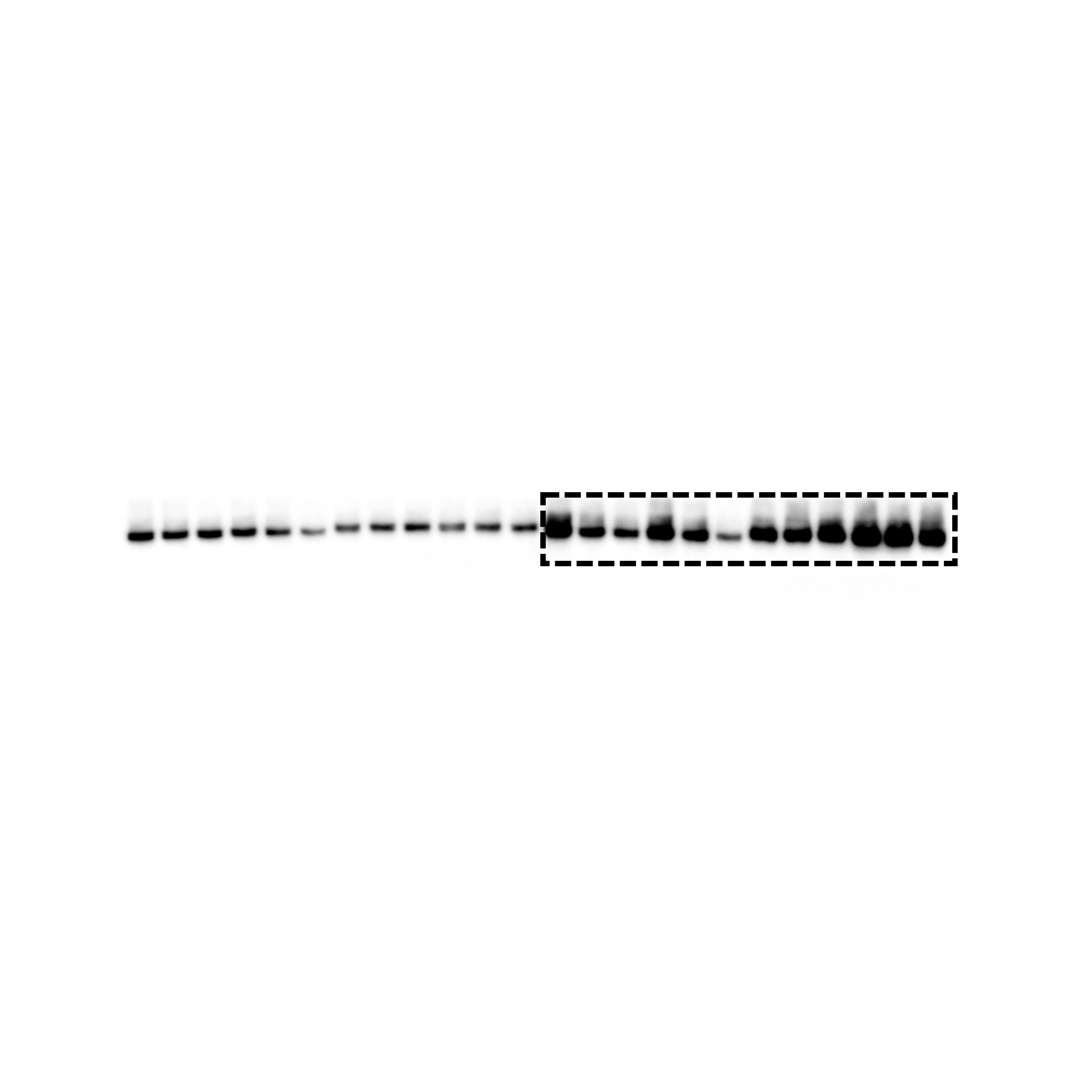

Supplement: Figure 7—source data 1. [file elife-98649-fig7-data1.zip › Figure 7-source data1/Figure 7D_p62_insoluble_annotated.tif]

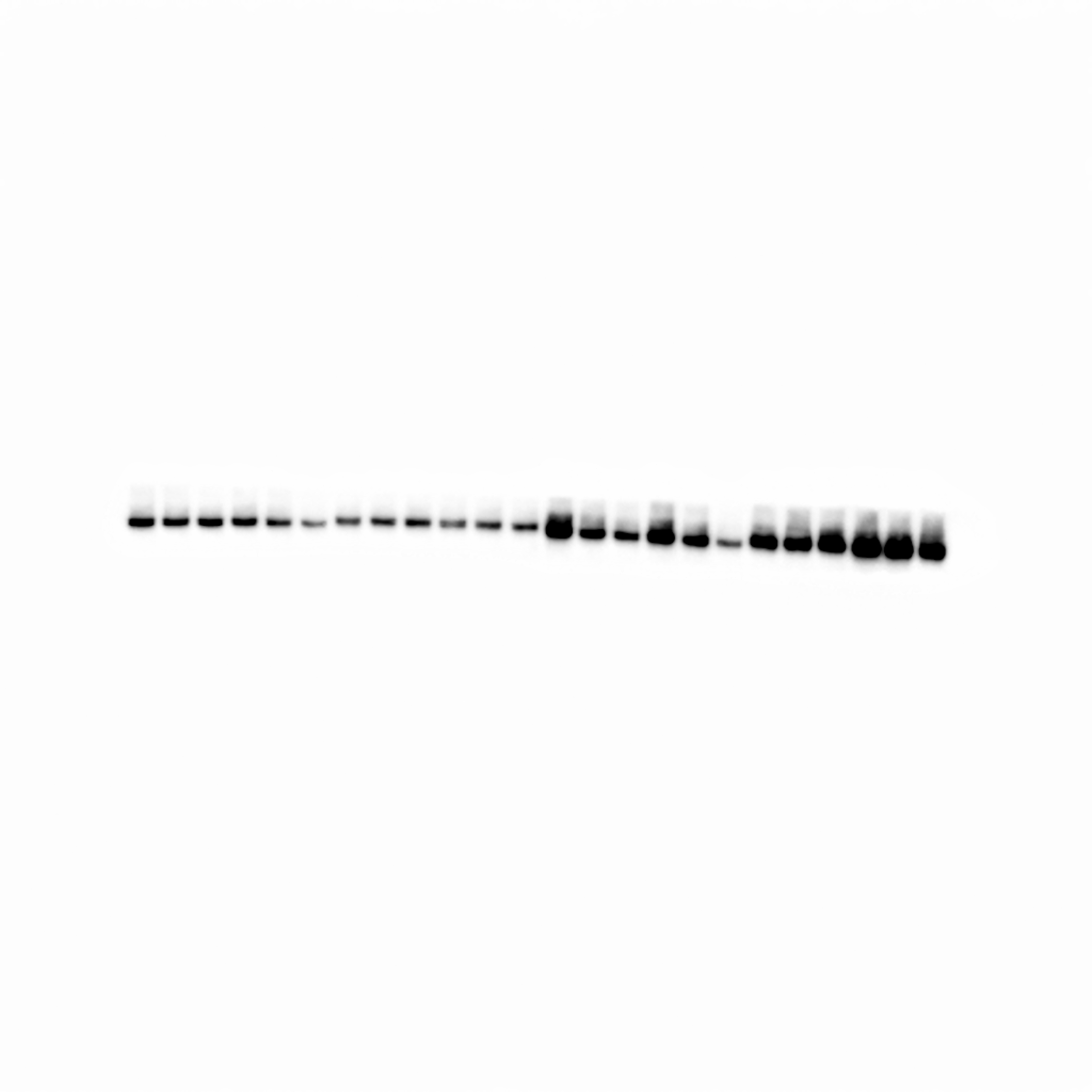

Supplement: Figure 7—source data 1. [file elife-98649-fig7-data1.zip › Figure 7-source data1/Figure 7D_p62_insoluble_raw.tif]

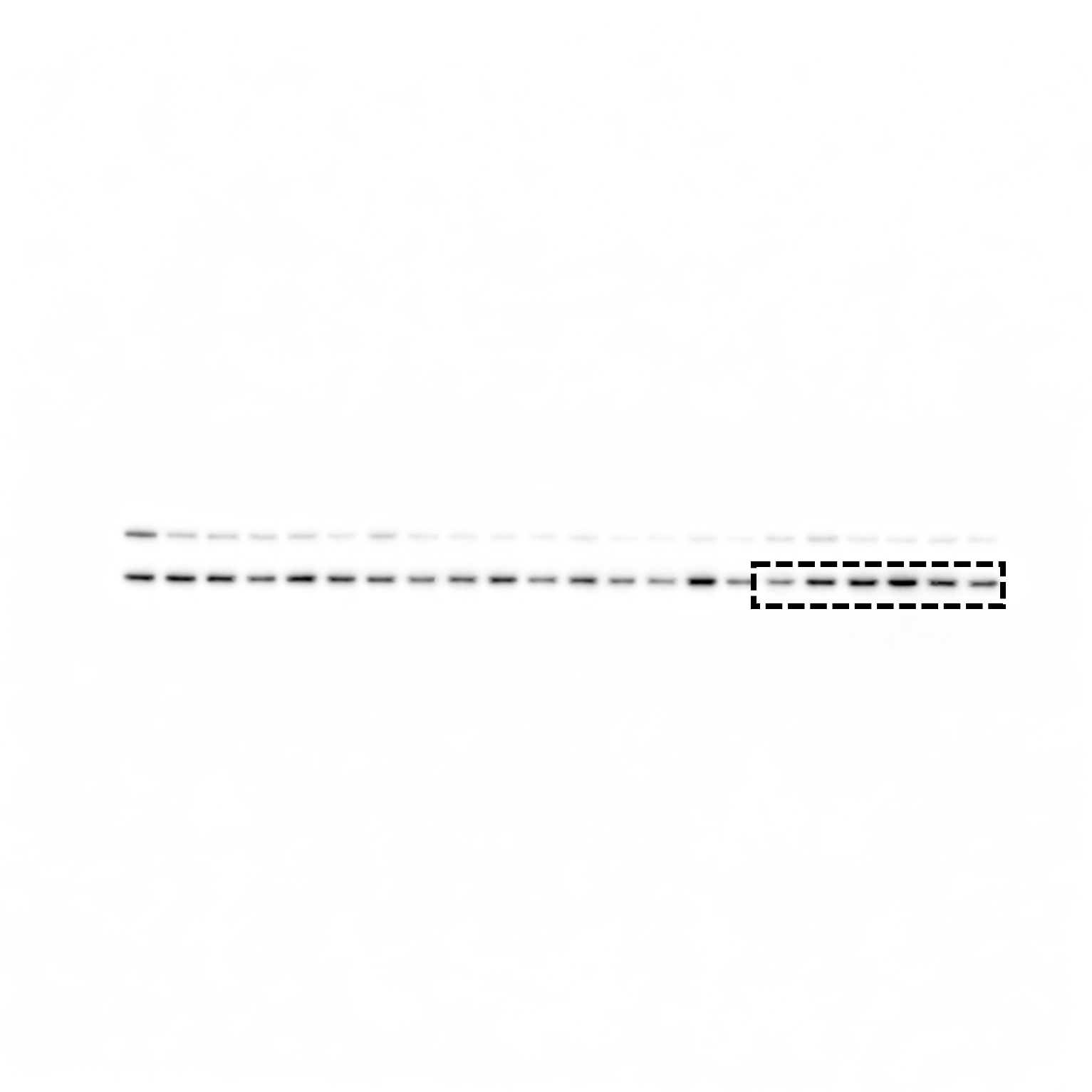

Supplement: Figure 8—source data 1. [file elife-98649-fig8-data1.zip › Figure 8-source data1/Figure 8D_GAPDH_soluble_annotated.tif]

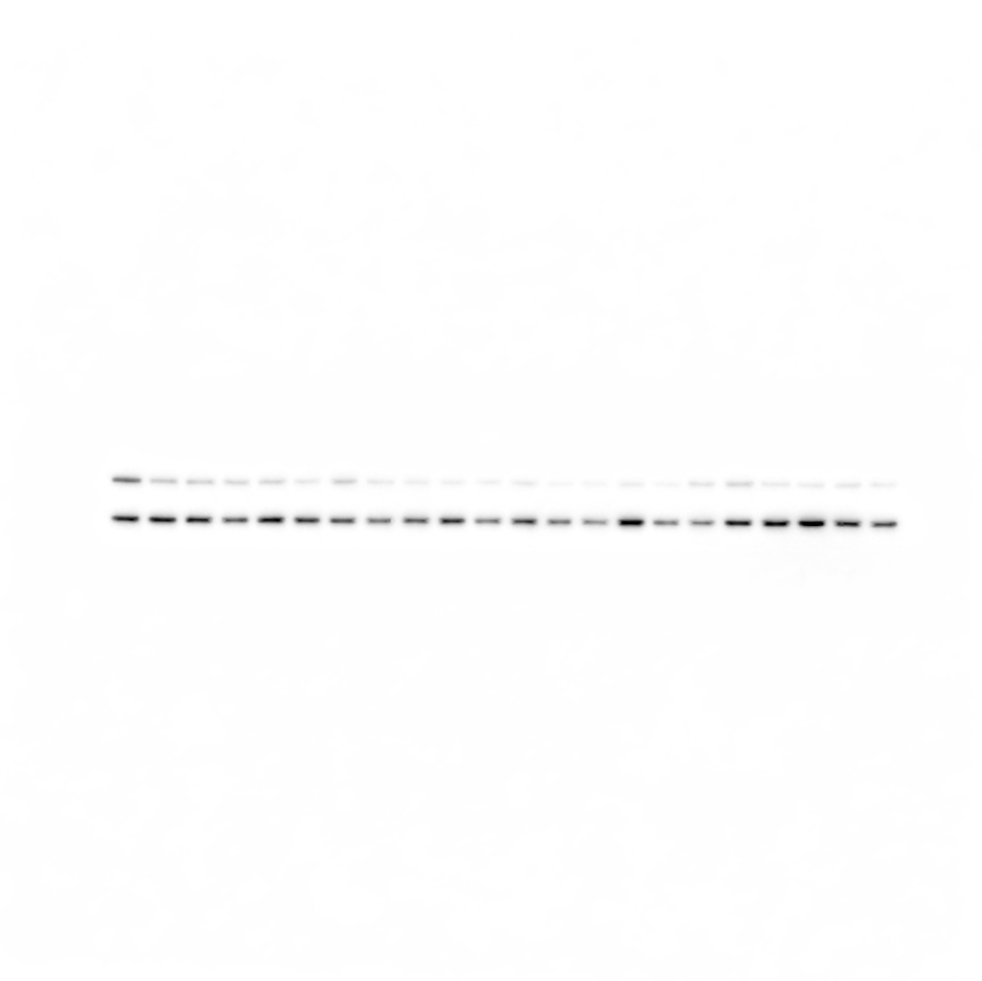

Supplement: Figure 8—source data 1. [file elife-98649-fig8-data1.zip › Figure 8-source data1/Figure 8D_GAPDH_soluble_raw.tif]

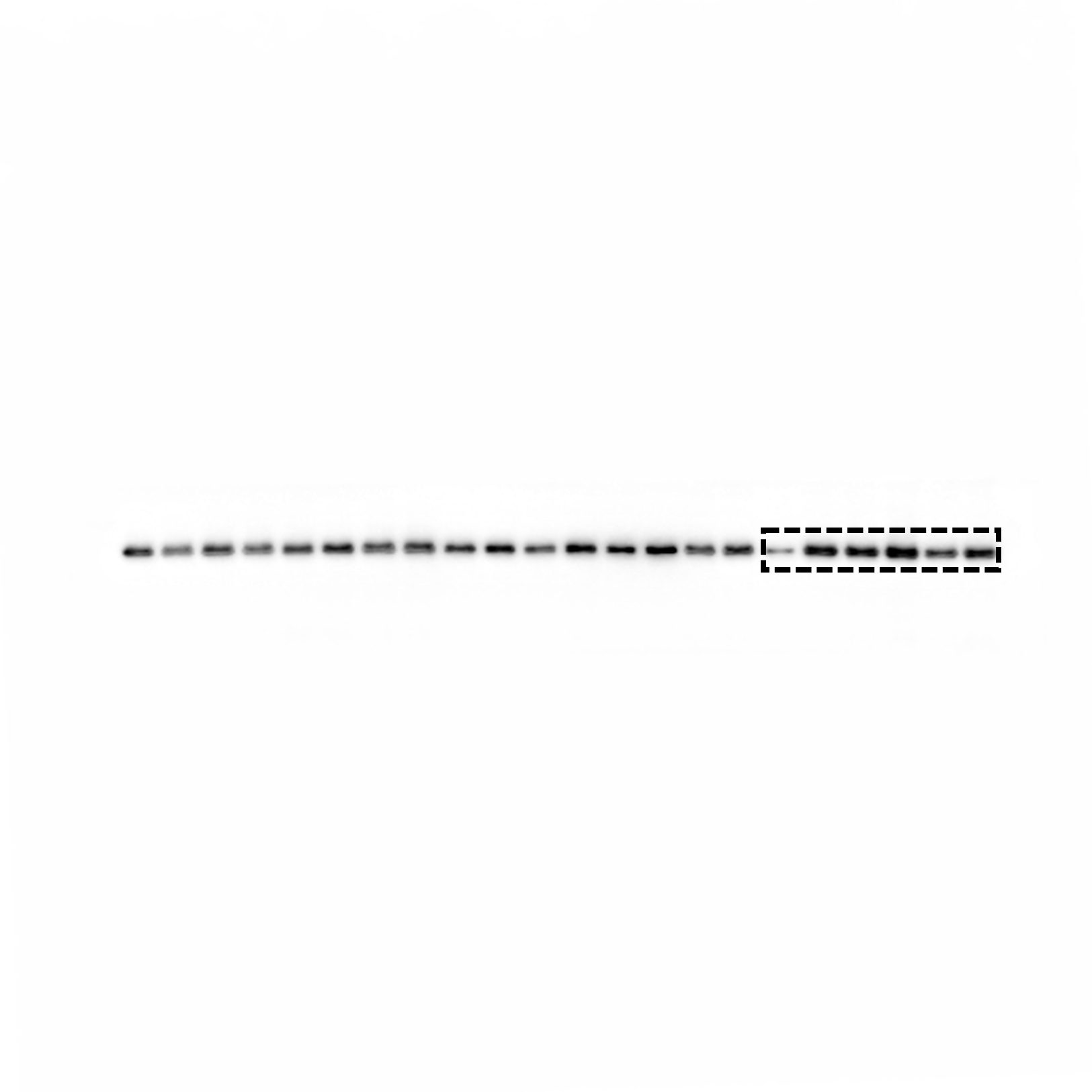

Supplement: Figure 8—source data 1. [file elife-98649-fig8-data1.zip › Figure 8-source data1/Figure 8D_GFP_insoluble_annotated.tif]

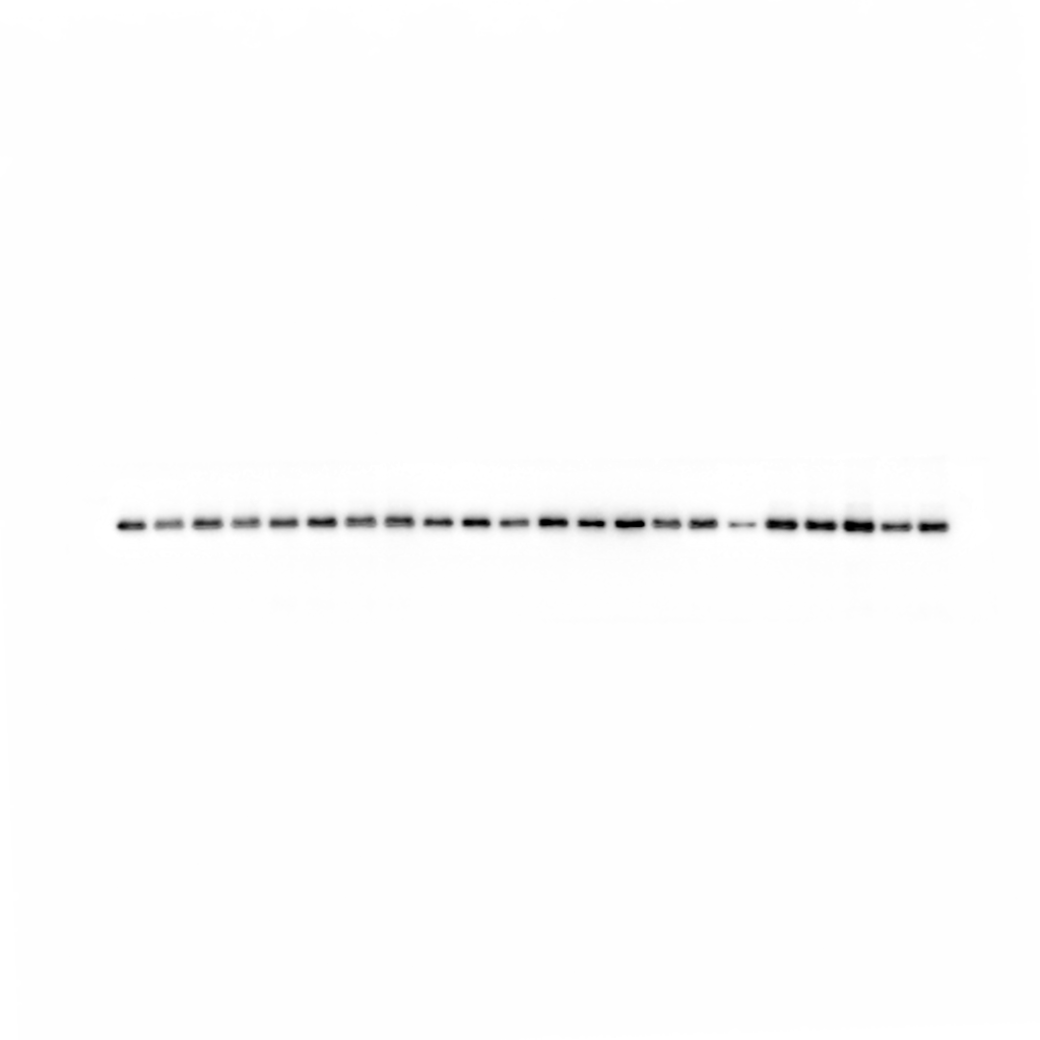

Supplement: Figure 8—source data 1. [file elife-98649-fig8-data1.zip › Figure 8-source data1/Figure 8D_GFP_insoluble_raw.tif]

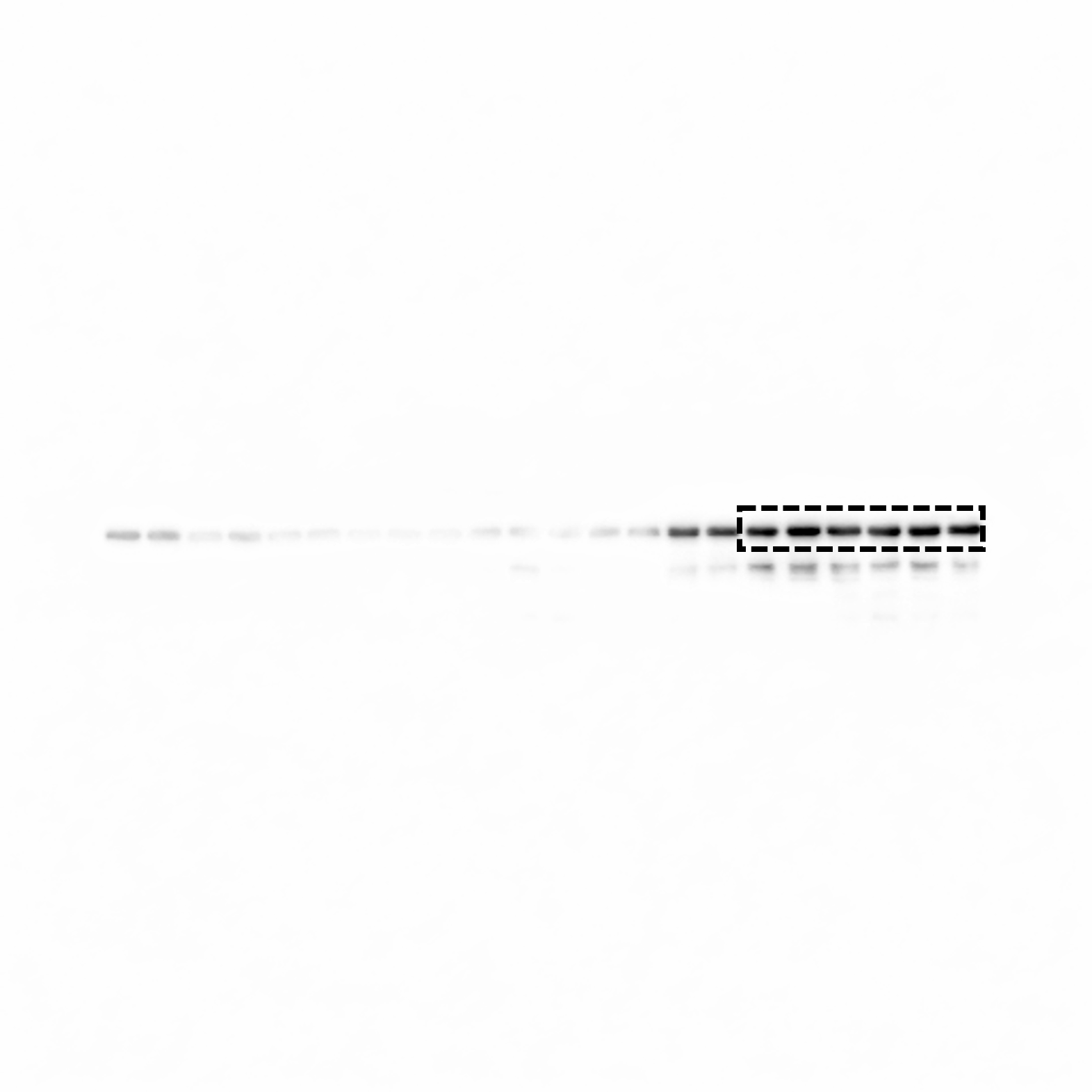

Supplement: Figure 8—source data 1. [file elife-98649-fig8-data1.zip › Figure 8-source data1/Figure 8D_GFP_soluble_annotated.tif]

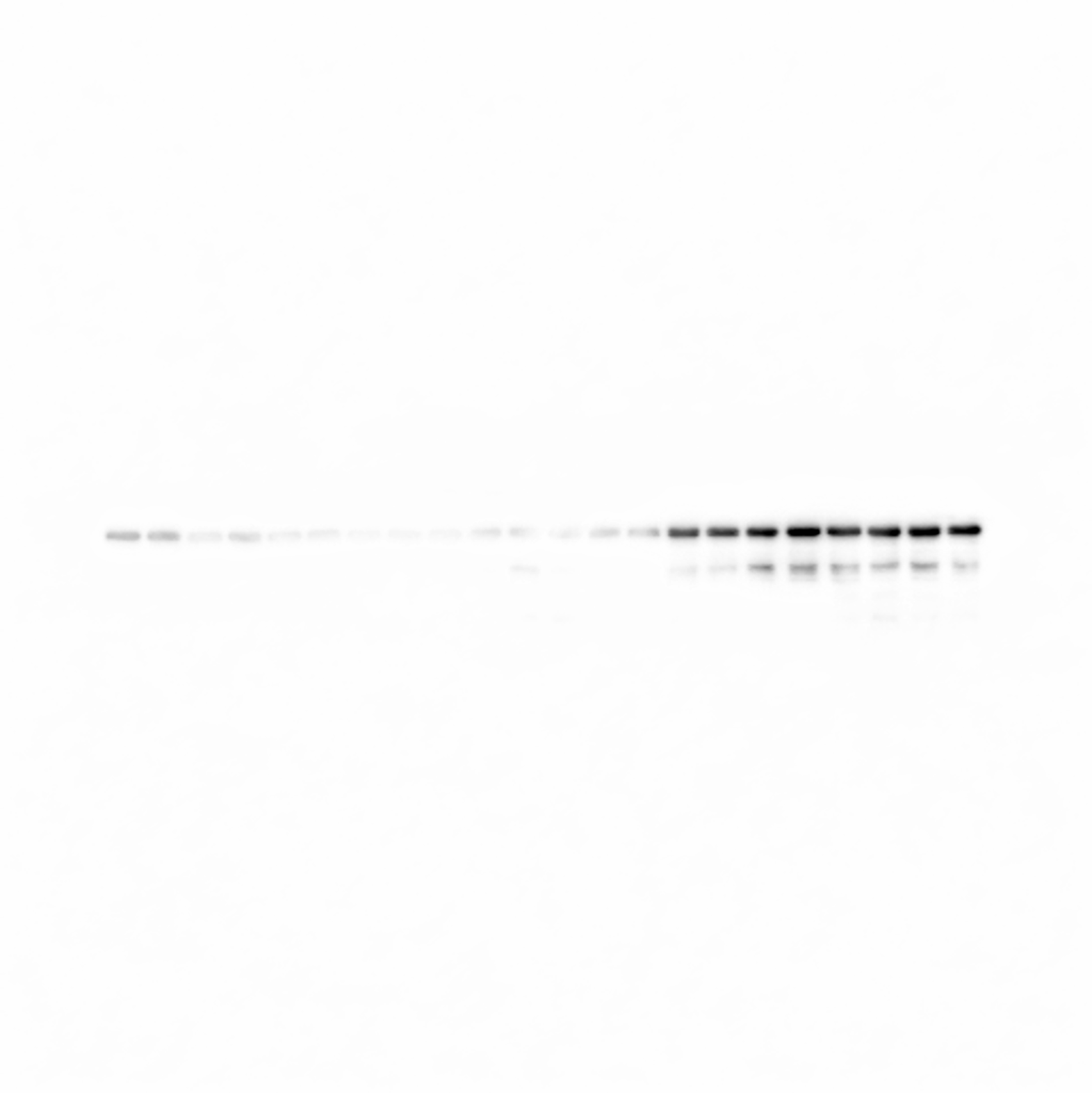

Supplement: Figure 8—source data 1. [file elife-98649-fig8-data1.zip › Figure 8-source data1/Figure 8D_GFP_soluble_raw.tif]

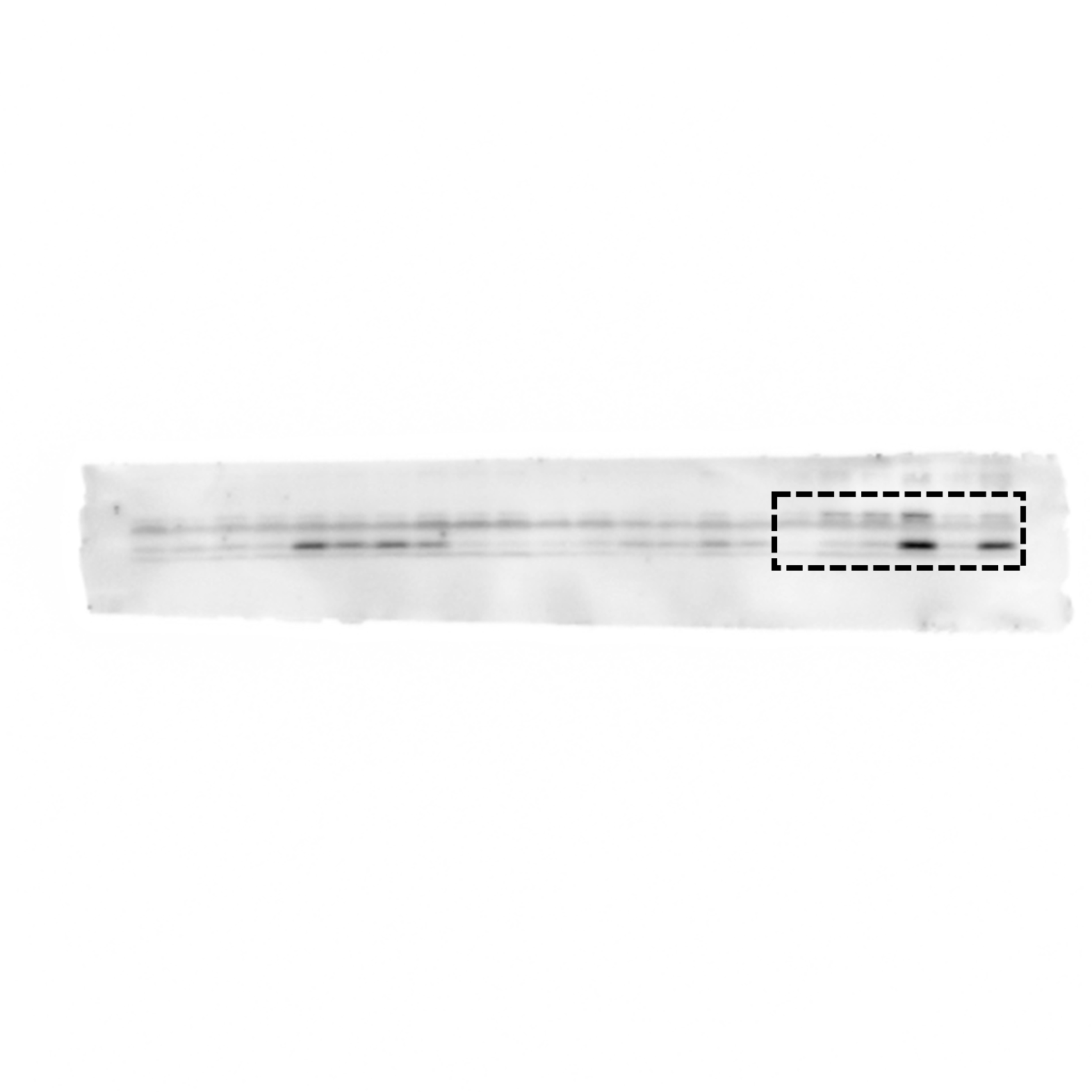

Supplement: Figure 8—source data 1. [file elife-98649-fig8-data1.zip › Figure 8-source data1/Figure 8D_LC3B_insoluble_anotated.tif]

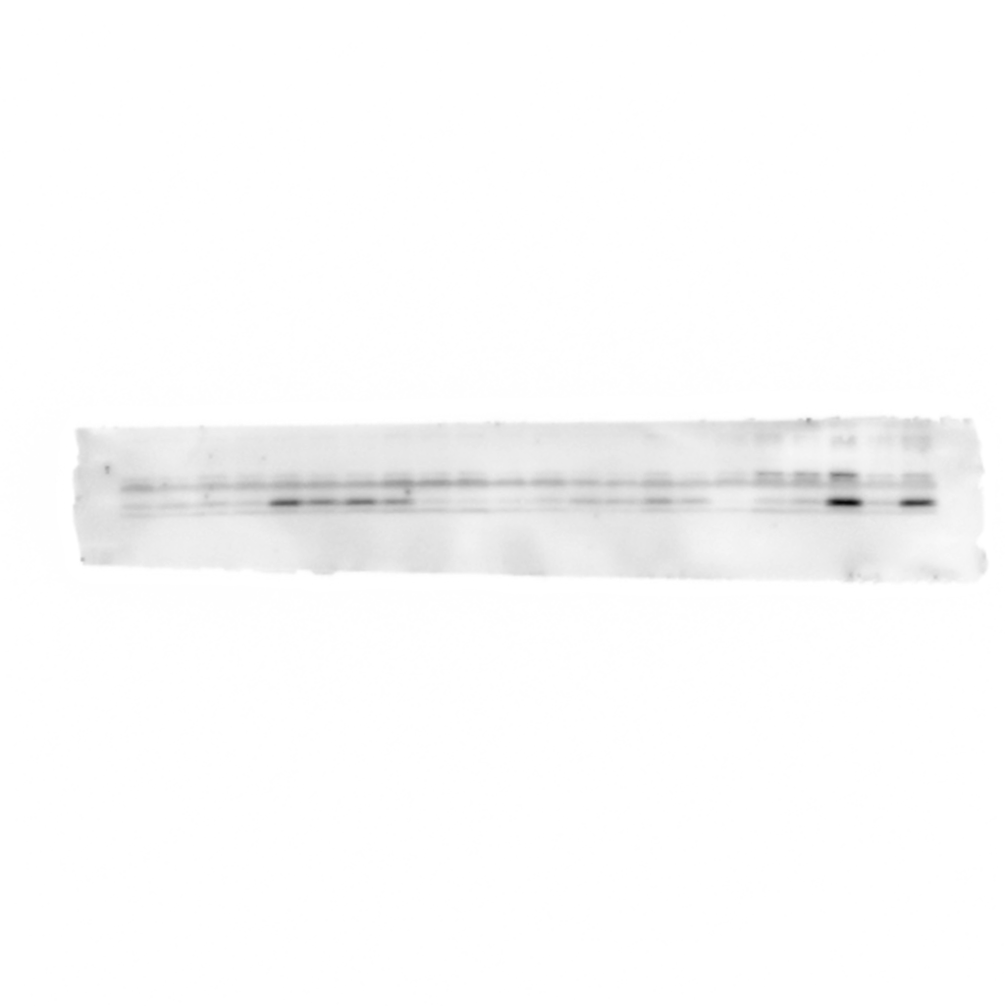

Supplement: Figure 8—source data 1. [file elife-98649-fig8-data1.zip › Figure 8-source data1/Figure 8D_LC3B_insoluble_raw.tif]

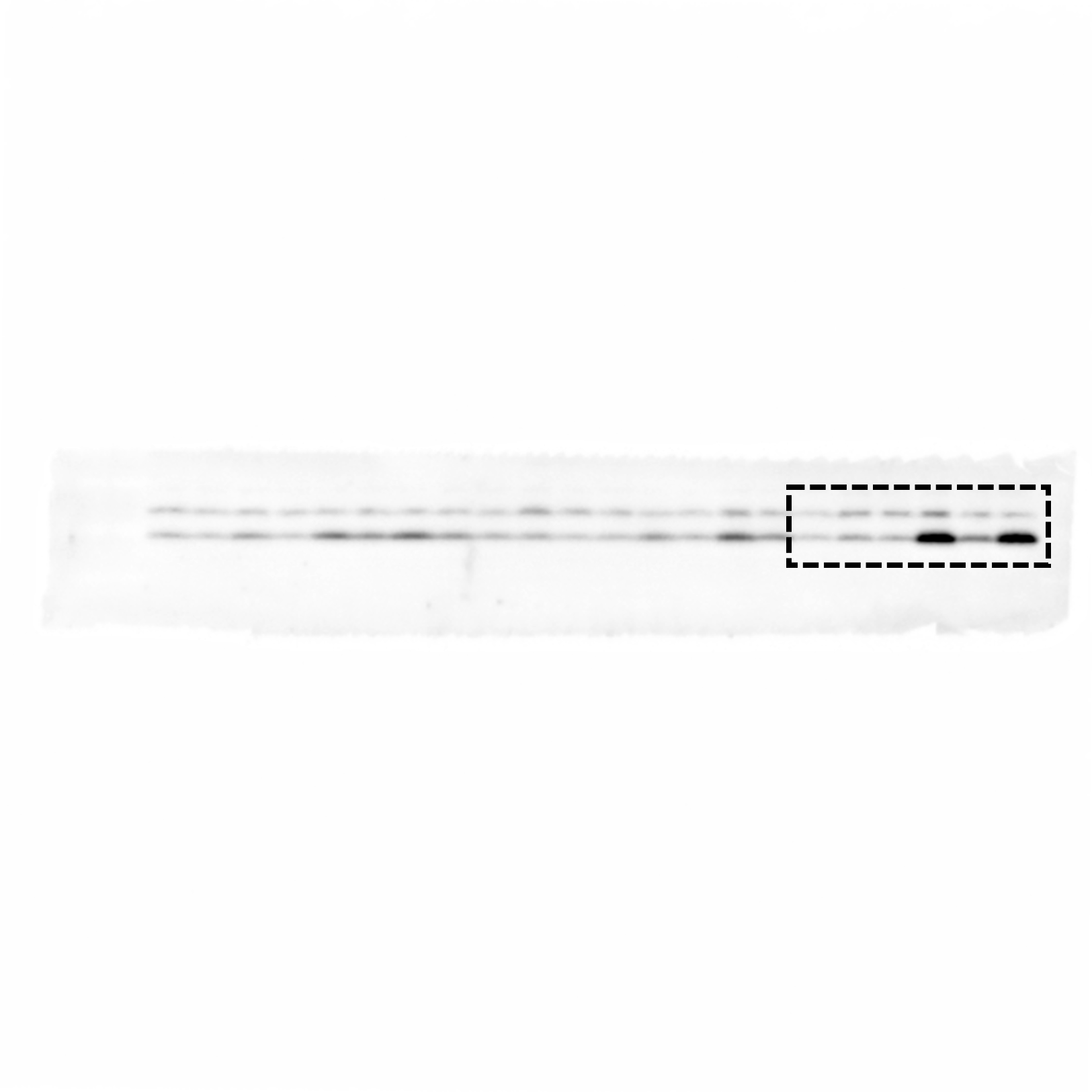

Supplement: Figure 8—source data 1. [file elife-98649-fig8-data1.zip › Figure 8-source data1/Figure 8D_LC3B_soluble_anotated.tif]

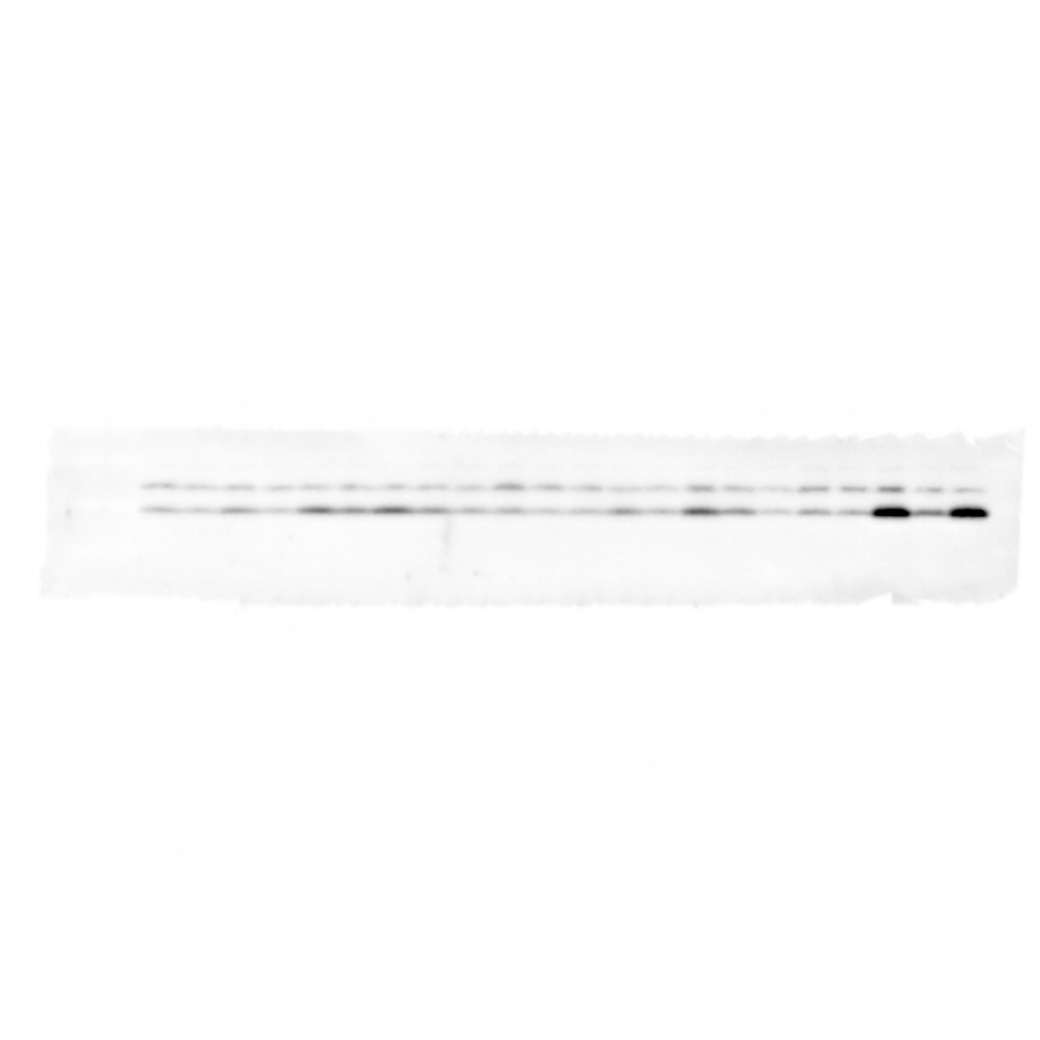

Supplement: Figure 8—source data 1. [file elife-98649-fig8-data1.zip › Figure 8-source data1/Figure 8D_LC3B_soluble_raw.tif]

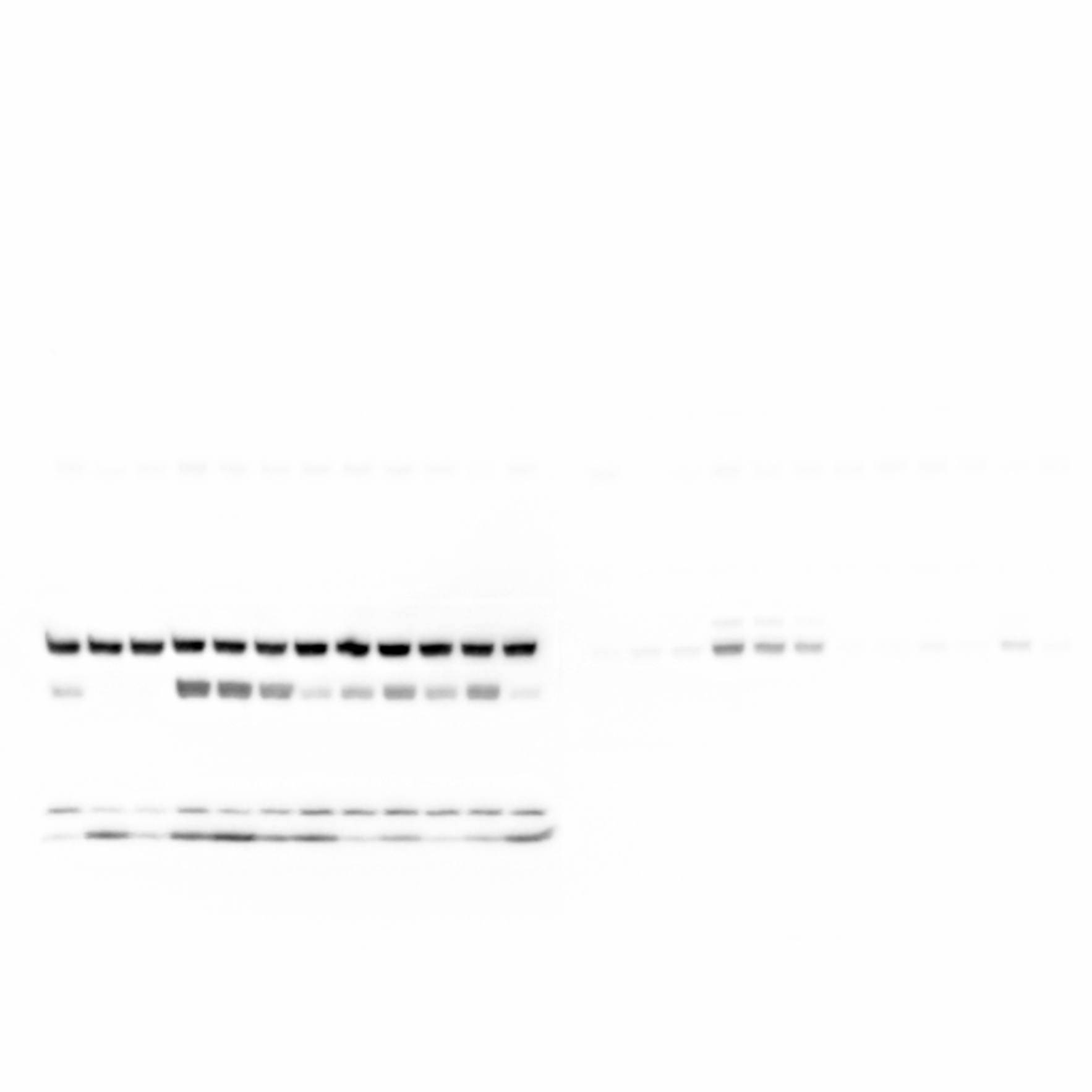

Supplement: Figure 9—source data 1. [file elife-98649-fig9-data1.zip › Figure 9-source data1/Figure 9C _p38_raw.tif]

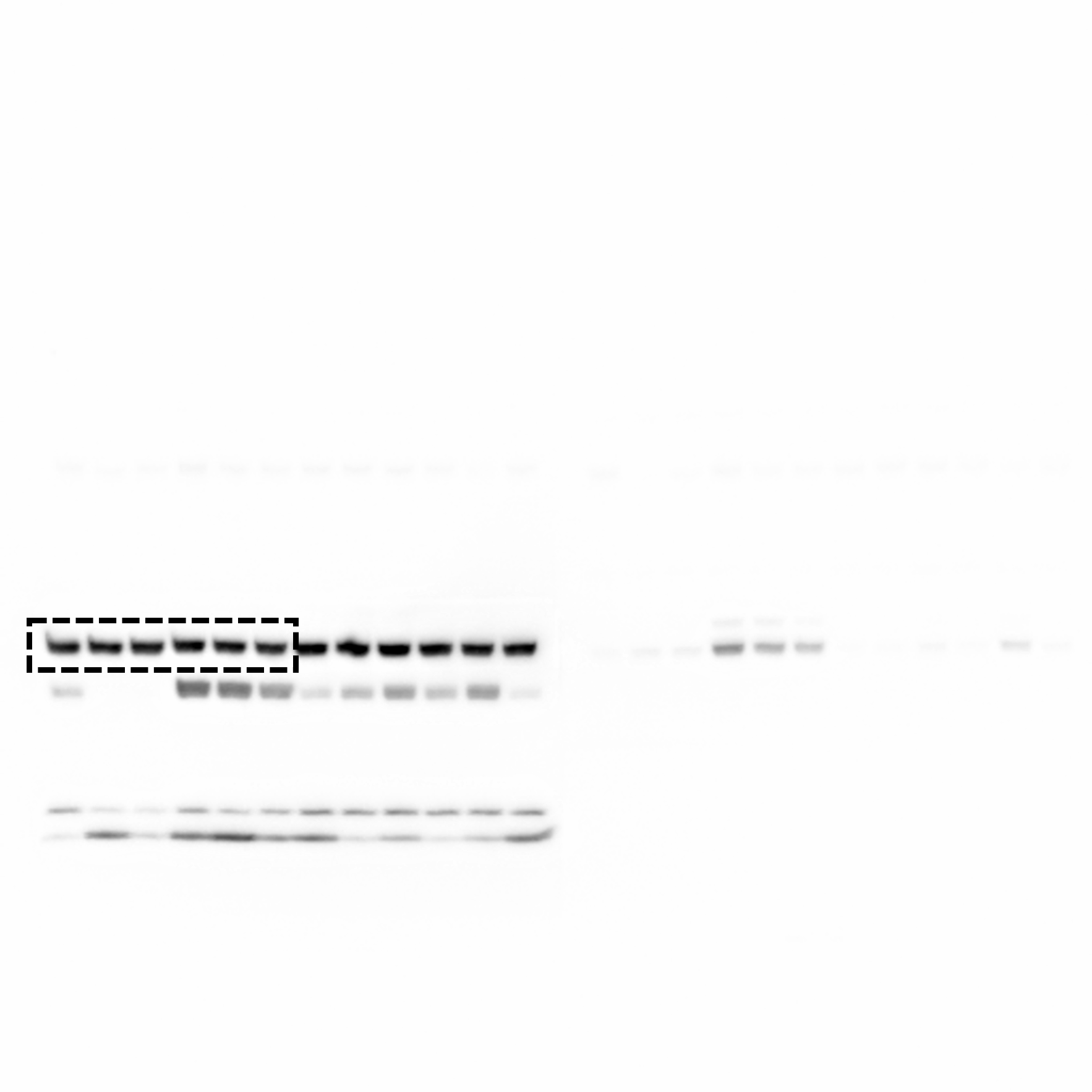

Supplement: Figure 9—source data 1. [file elife-98649-fig9-data1.zip › Figure 9-source data1/Figure 9C_p38_annotated.tif]

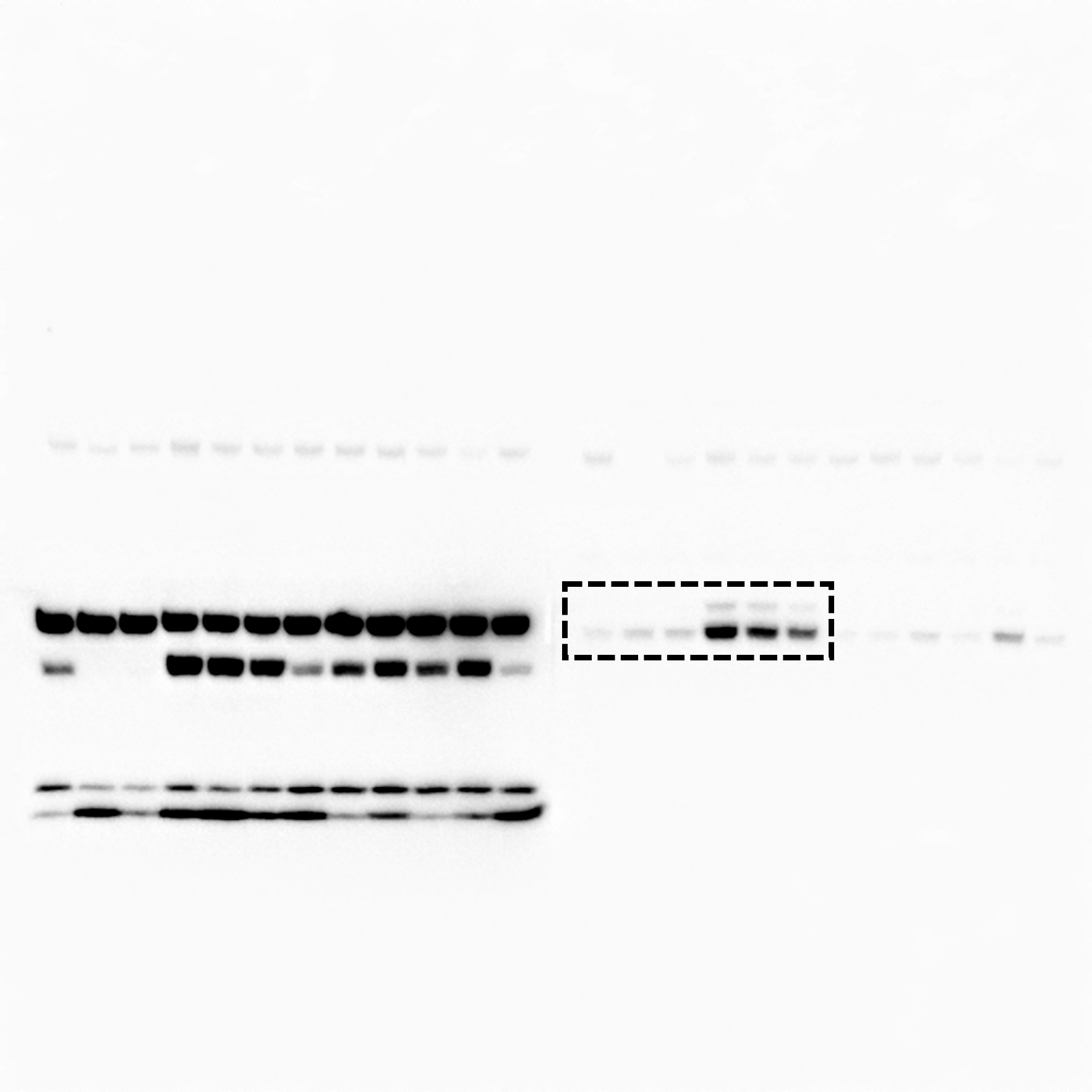

Supplement: Figure 9—source data 1. [file elife-98649-fig9-data1.zip › Figure 9-source data1/Figure 9C_p-p38_annotatedtif.tif]

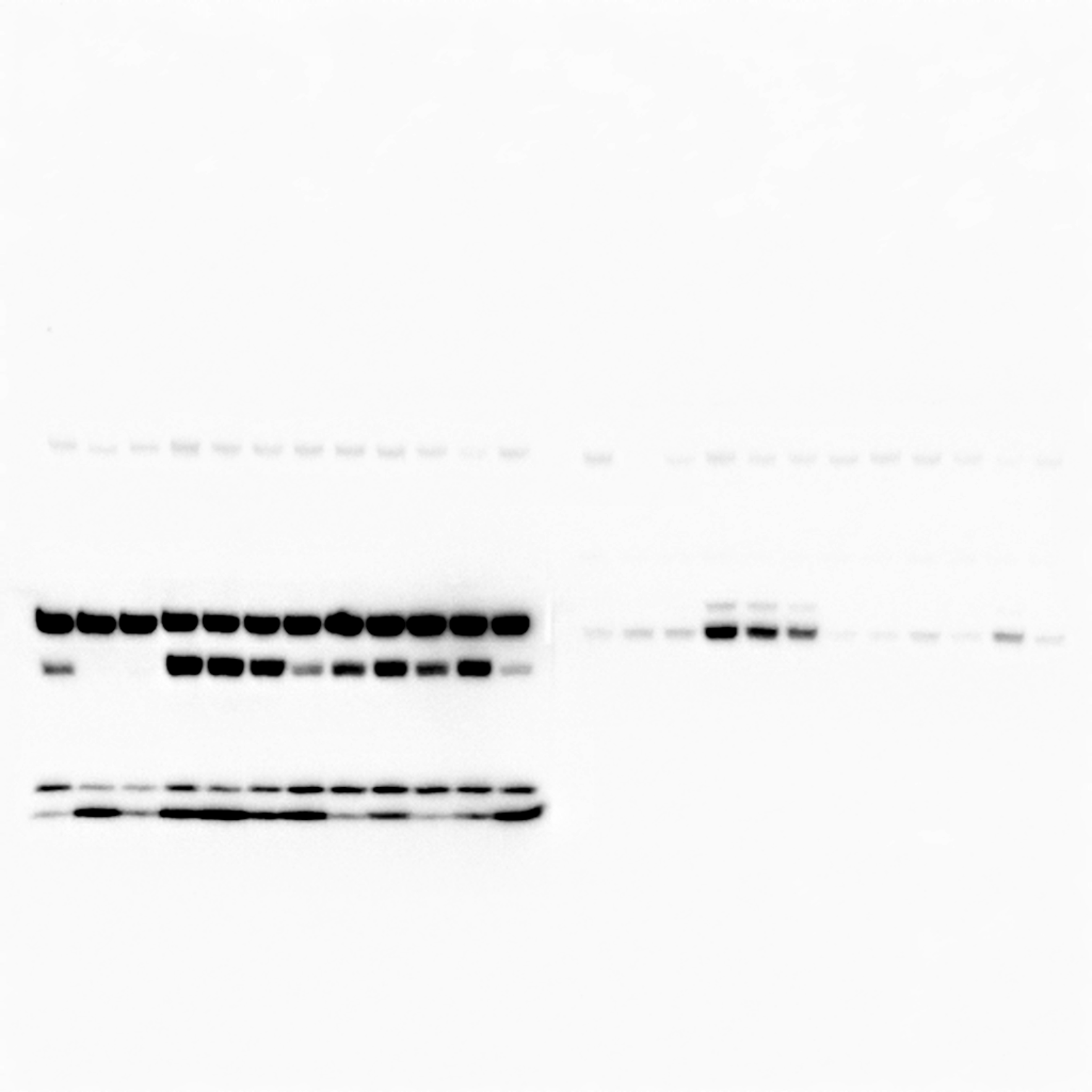

Supplement: Figure 9—source data 1. [file elife-98649-fig9-data1.zip › Figure 9-source data1/Figure 9C_p-p38_raw.tif]

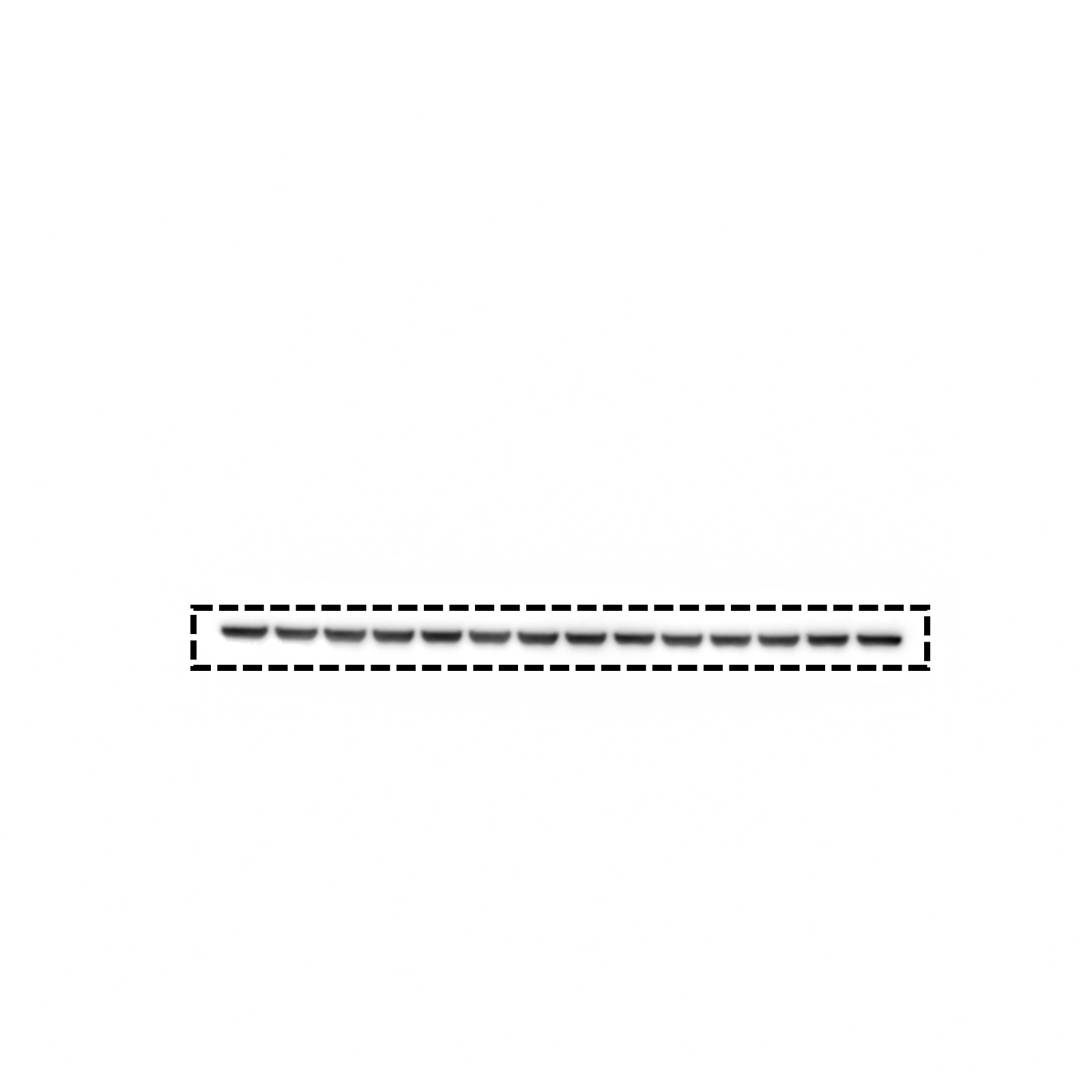

Supplement: Figure 10—source data 1. [file elife-98649-fig10-data1.zip › Figure 10-source data1/Figure 10B_actin_annotated.tif]

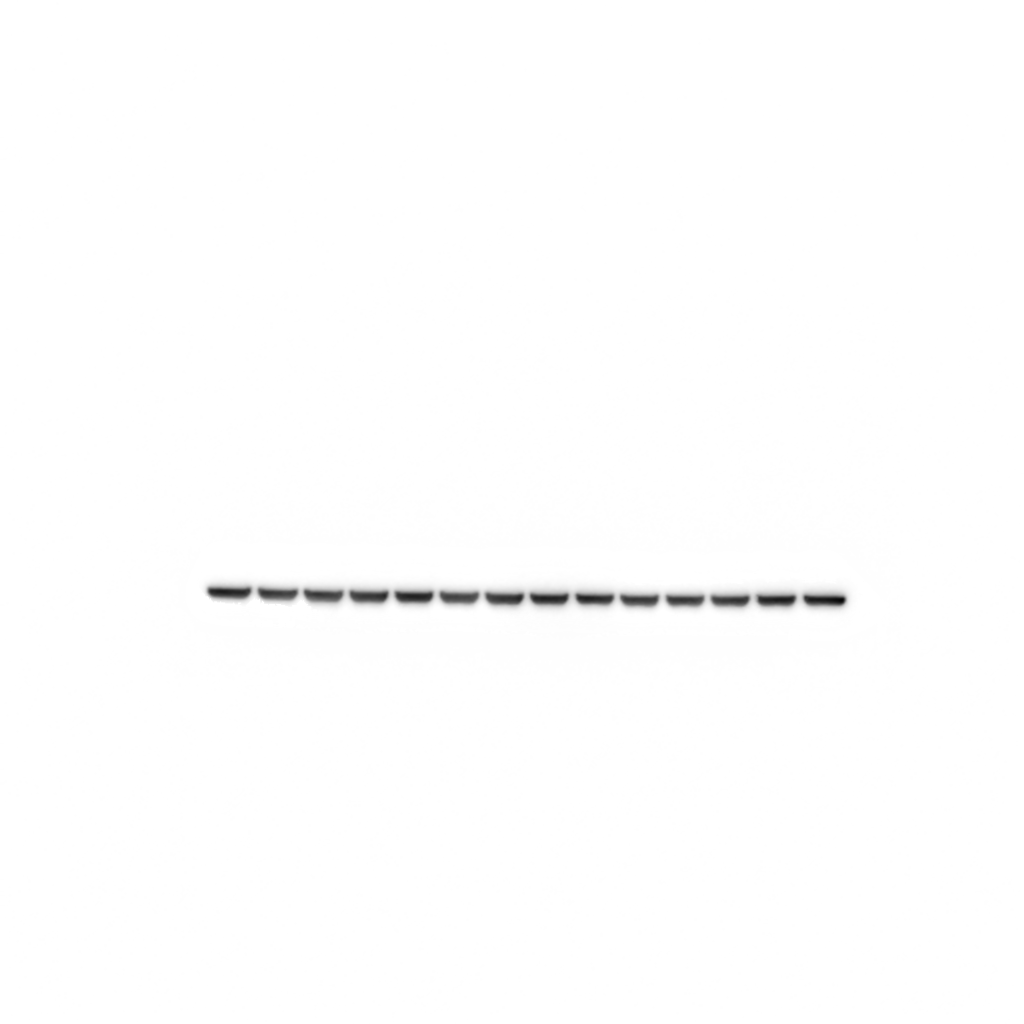

Supplement: Figure 10—source data 1. [file elife-98649-fig10-data1.zip › Figure 10-source data1/Figure 10B_actin_raw.tif]

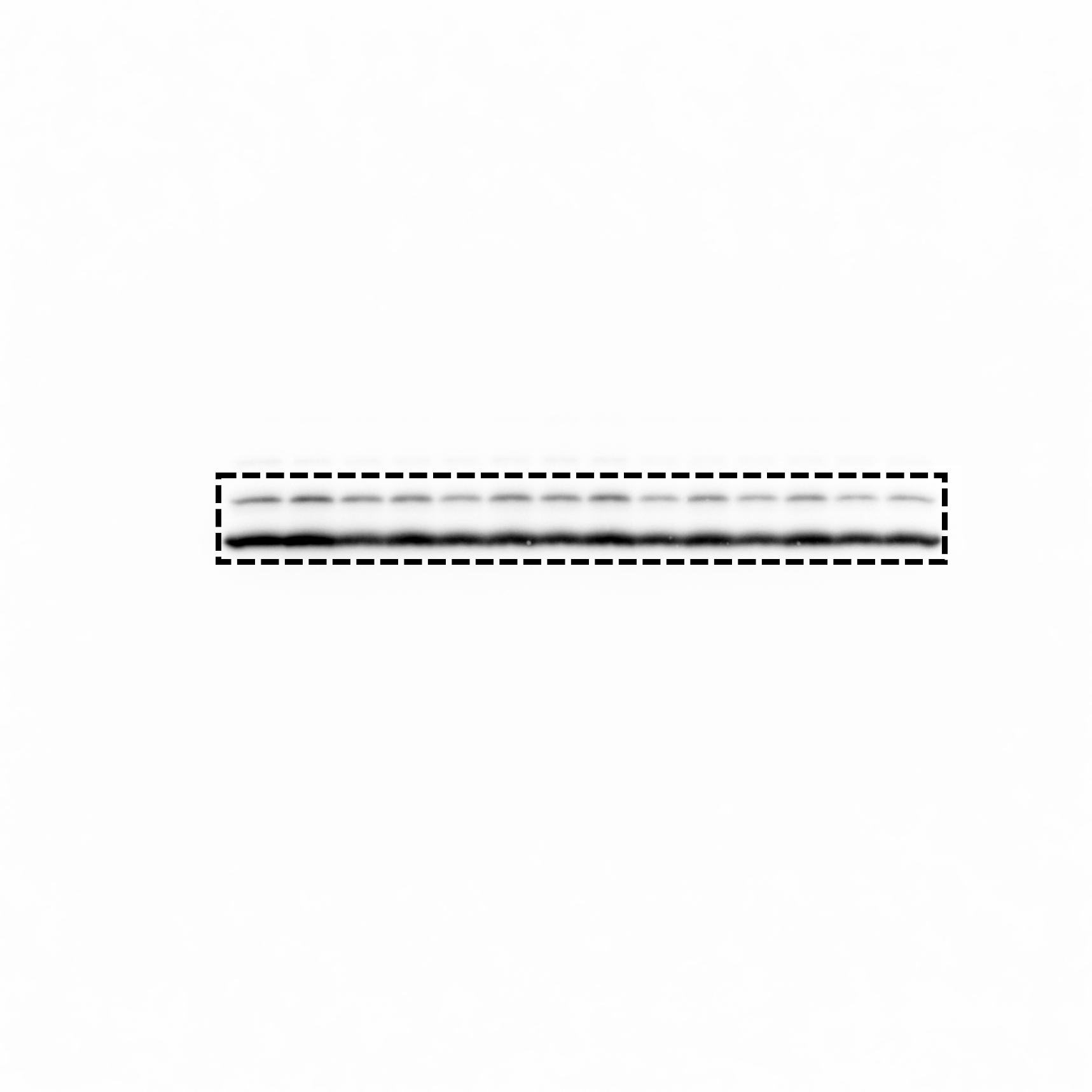

Supplement: Figure 10—source data 1. [file elife-98649-fig10-data1.zip › Figure 10-source data1/Figure 10B_LC3B_annotated.tif]

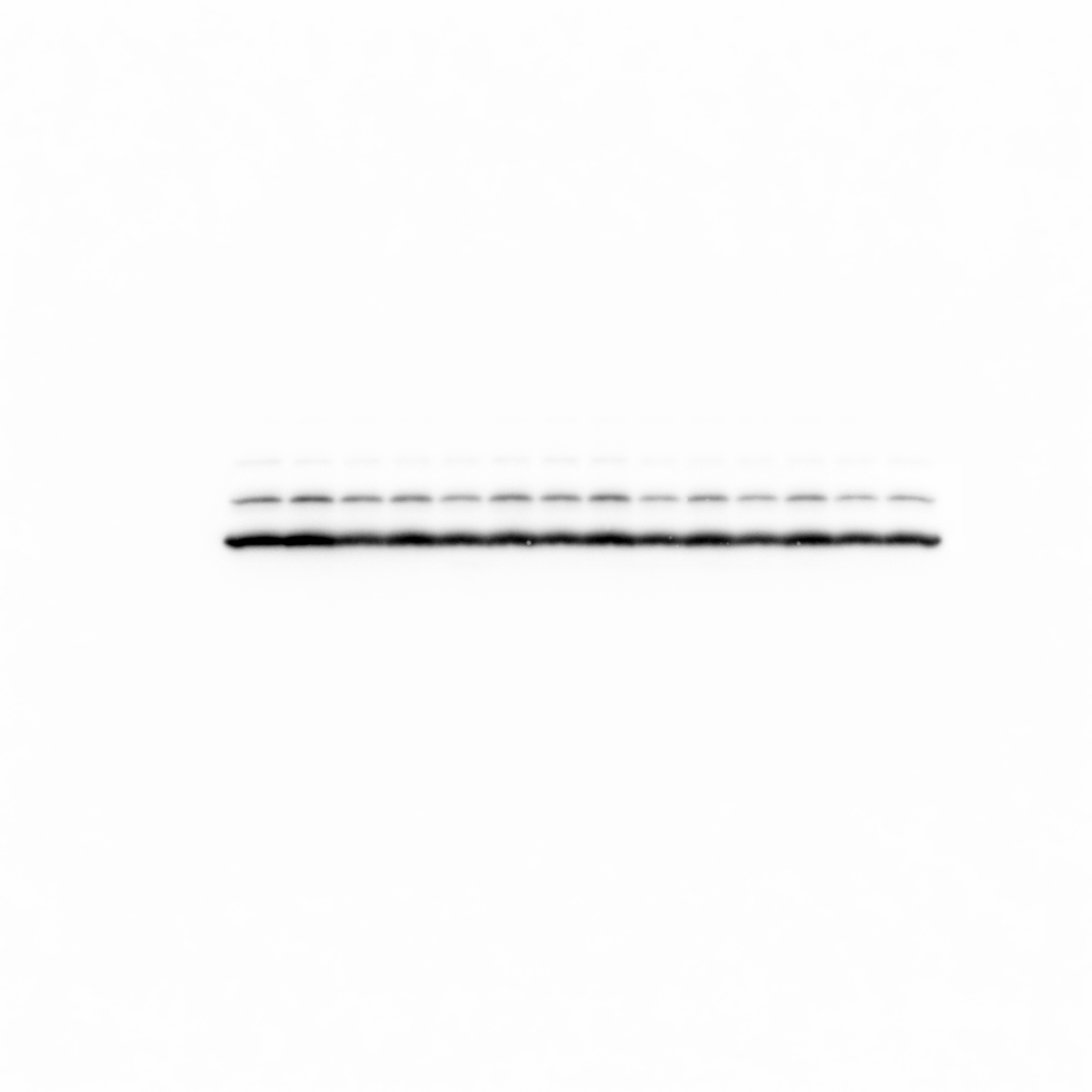

Supplement: Figure 10—source data 1. [file elife-98649-fig10-data1.zip › Figure 10-source data1/Figure 10B_LC3B_raw.tif]
